# Causal effects of COVID-19 on structural changes in specific brain regions: a Mendelian randomization study

**DOI:** 10.1186/s12916-023-02952-1

**Published:** 2023-07-19

**Authors:** Shaojiong Zhou, Tao Wei, Xiaoduo Liu, Yufei Liu, Weiyi Song, Xinwei Que, Yi Xing, Zhibin Wang, Yi Tang

**Affiliations:** 1 Department of Neurology & Innovation Center for Neurological Disorders, Xuanwu Hospital, Capital Medical University, National Center for Neurological Disorders, 45 Changchun Street, Beijing, 100053 China; 2 Neurodegenerative Laboratory of Ministry of Education of the Peoples Republic of China, Beijing, China

**Keywords:** COVID-19, Brain structure, Mendelian randomization, Causality

## Abstract

**Background:**

Previous studies have found a correlation between coronavirus disease 2019 (COVID-19) and changes in brain structure and cognitive function, but it remains unclear whether COVID-19 causes brain structural changes and which specific brain regions are affected. Herein, we conducted a Mendelian randomization (MR) study to investigate this causal relationship and to identify specific brain regions vulnerable to COVID-19.

**Methods:**

Genome-wide association study (GWAS) data for COVID-19 phenotypes (28,900 COVID-19 cases and 3,251,161 controls) were selected as exposures, and GWAS data for brain structural traits (cortical thickness and surface area from 51,665 participants and volume of subcortical structures from 30,717 participants) were selected as outcomes. Inverse-variance weighted method was used as the main estimate method. The weighted median, MR-Egger, MR-PRESSO global test, and Cochran’s *Q* statistic were used to detect heterogeneity and pleiotropy.

**Results:**

The genetically predicted COVID-19 infection phenotype was nominally associated with reduced cortical thickness in the caudal middle frontal gyrus (*β* = − 0.0044, *p* = 0.0412). The hospitalized COVID-19 phenotype was nominally associated with reduced cortical thickness in the lateral orbitofrontal gyrus (*β* = − 0.0049, *p* = 0.0328) and rostral middle frontal gyrus (*β* = − 0.0022, *p* = 0.0032) as well as with reduced cortical surface area of the middle temporal gyrus (*β* = − 10.8855, *p* = 0.0266). These causal relationships were also identified in the severe COVID-19 phenotype. Additionally, the severe COVID-19 phenotype was nominally associated with reduced cortical thickness in the cuneus (*β* = − 0.0024, *p* = 0.0168); reduced cortical surface area of the pericalcarine (*β* = − 2.6628, *p* = 0.0492), superior parietal gyrus (*β* = − 5.6310, *p* = 0.0408), and parahippocampal gyrus (*β* = − 0.1473, *p* = 0.0297); and reduced volume in the hippocampus (*β* = − 15.9130, *p* = 0.0024).

**Conclusions:**

Our study indicates a suggestively significant association between genetic predisposition to COVID-19 and atrophy in specific functional regions of the human brain. Patients with COVID-19 and cognitive impairment should be actively managed to alleviate neurocognitive symptoms and minimize long-term effects.

**Supplementary Information:**

The online version contains supplementary material available at 10.1186/s12916-023-02952-1.

## Background

Coronavirus disease 2019 (COVID-19) has imposed a large burden on public health. As of November 20, 2022, 634 million confirmed cases of COVID-19 worldwide, including 6.6 million deaths, have been reported to the World Health Organization. Almost 3 years into the pandemic, it has been recognized that some patients infected with COVID-19 suffer long-term symptoms, which are collectively referred to as “long COVID”; this discovery poses new clinical challenges. Although COVID-19 is predominantly a respiratory disease, studies have documented a broad spectrum of neuropsychiatric manifestations, such as hyposmia, cognitive impairment, and “brain fog,” during acute COVID-19 [1, 2]; more concerningly, 25.9% of patients with COVID-19 experience residual neuropsychiatric symptoms that persist up to 20 months post-infection [3, 4]. These findings suggest that COVID-19 may have adverse effects on brain structures.

A magnetic resonance imaging (MRI)-based longitudinal study investigating 401 COVID-19 cases from the UK Biobank (in patients aged 51–81 years) identified significantly reduced cortical thickness in the orbitofrontal cortex and parahippocampal gyrus as well as changes in markers of brain tissue damage in regions functionally connected to the primary olfactory cortex [5]. Two positron emission tomography (PET) cohort studies investigated correlates of cognitive impairment and found that the orbital gyrus rectus, right medial temporal lobe, and frontoparietal regions displayed hypometabolism in patients with COVID-19 at a subacute stage [6, 7]. At a histopathological level, inflammation, hypoxia, and coagulation disorder are the three most common abnormalities in the brain tissue of patients with severe COVID-19 at the acute stage [8], presumably as consequences of viral invasion [9, 10], viral-induced neuroinflammation or immune response in the brain [1, 11], hypoxemia [8], and blood-brain barrier dysfunction [12]. In addition, persistent hyposmia or anosmia after severe acute respiratory syndrome coronavirus 2 (SARS-CoV-2) infection in the absence of nasal symptoms is a relatively specific manifestation of COVID-19, suggesting damage to the olfactory pathway [13–16]. Further studies are needed to investigate the effects of COVID-19 on structures of specific brain functional regions.

Neuropsychiatric manifestations are not exclusive to patients with moderate and severe COVID-19. In fact, cognitive impairment is also prevalent in non-hospitalized patients with mild COVID-19, occurring in 1.67% of females and 3.81% of males [4]. Most patients infected with the omicron variant of SARS-CoV-2 were asymptomatic or mildly symptomatic, but the incidence of long COVID associated with these infections was as high as 4.5% [17]. However, neuroimaging data for assessing brain structural changes is lacking in most of these mild cases. In addition, studies on the effects of COVID-19 on brain structures or cognitive function could be confounded by situational factors that affected many people during the pandemic. For example, patients with COVID-19 were often segregated in restricted areas and confined in individual rooms, potentially exacerbating brain atrophy [18]. SARS-CoV-2 infects a substantial proportion of elderly individuals with age-related brain atrophy and cognitive decline, which can complicate efforts to attribute these phenotypes to COVID-19 [19, 20]. Therefore, there is an urgent need to determine whether COVID-19 can potentiate structural changes in specific brain functional regions.

Mendelian randomization (MR) is an analytical method that uses single-nucleotide polymorphisms (SNPs) as instrumental variables (IVs) to make causal inferences between exposures and outcomes. Random assortment during meiosis effectively divides a population of SNPs into effect and control groups for the risk factor based on the genetic profile of each individual, akin to a randomized controlled trial [21]. In this study, using large-scale genome-wide association study (GWAS) data, we performed a two-sample MR analysis to appraise the causal effects of COVID-19 (the exposure) on cortical and subcortical structures (the outcome), defined as MRI-derived morphometric indicators of cortical thickness, cortical surface area, and volume of subcortical structures. We found that COVID-19 potentially caused atrophy in specific brain functional regions, and more extensive brain atrophy may result from severe COVID-19. Our study provides new evidence for a causal relationship between COVID-19 and brain structural changes and suggests possible causes of cognitive impairment after COVID-19.

## Methods

**Fig. 1:**
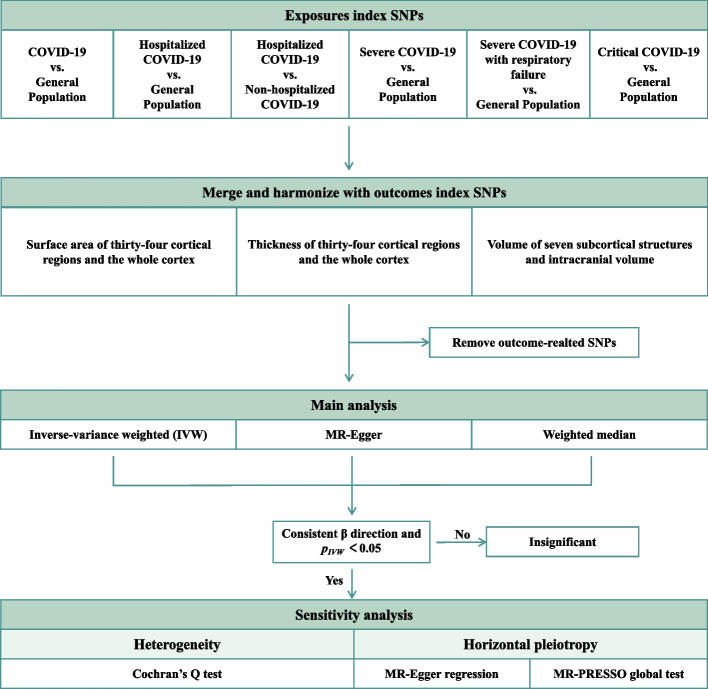
Overall design of the MR analysis in the present study. Abbreviations: COVID-19, coronavirus disease 2019; IVW, inverse-variance weighted; MR, Mendelian randomization; MR-PRESSO, Mendelian randomization-pleiotropy residual sum and outlier; nSNPs, number of single-nucleotide polymorphisms

Figure 1 displays a schematic of our study design. This study is reported in accordance with the Strengthening the Reporting of Observational Studies in Epidemiology (STROBE) reporting guidelines (Additional file 1: Table S1) [22].

### Study population

Data for COVID-19 phenotypes were obtained as exposures from GWAS datasets provided by the COVID-19 Host Genetics Initiative. The GWAS datasets were adjusted for age, age^2^, sex, age × sex, principal components, and study-specific covariates of each contributing cohort [23]. For the COVID-19 infection phenotype, we included 14,134 confirmed cases of COVID-19 with 1,284,876 population controls [23]. For the hospitalized COVID-19 phenotype, we included 6406 hospitalized COVID-19 cases with 902,088 population controls as well as 1776 hospitalized cases with 6443 non-hospitalized control cases [23]. For the severe COVID-19 phenotype, we included 4792 confirmed cases of very severe respiratory infections with 1,054,664 population controls [23], 1610 confirmed severe COVID-19 cases with respiratory failure and 2180 population controls [24], and 182 confirmed critical illness cases with 910 population controls [25].

Data for brain structural traits were selected as outcomes from a GWAS of MRI-derived brain morphometry conducted by the ENIGMA consortium [26, 27]. The covariates adjusted for volume, included age, sex, age^2^, four multidimensional scaling, intracranial volume, and site [27]. For cortical thickness and surface area, 51,665 individuals from 60 cohorts across the globe were included. Thirty-four brain regions and the whole cortex were defined using the Desikan-Killiany cortical atlas, and estimates were weighted by the entire brain. For the volume of subcortical structures, seven brain regions were measured in 30,717 participants, including thalamus volume, nucleus accumbens volume, putamen volume, caudate volume, amygdala volume, hippocampus volume, and pallidum volume, which were all adjusted by intracranial volume. Phenotypes were defined as the mean estimates of the left and right hemispheres (thickness was calculated in mm, surface area in mm^2^, and volume of subcortical structures in cm^3^).

### Genetic instruments

First, after determining that few SNPs met the significance threshold of 5 × 10^−8^ [28, 29], we set a relatively relaxed threshold of 1 × 10^−5^. Second, linkage disequilibrium (LD) clumping was performed to identify the independent SNPs (*r*^2^ threshold < 0.001 within a 10 Mb window) [30]. When no SNP in the outcome dataset met this criterion, proxy SNPs with LD set at *r*^2^ > 0.8 were used. To ensure the strength of the chosen SNPs, we also calculated the *F* statistic, and an *F* statistic of 10 was regarded as sufficiently robust to counteract weak instrument bias [31]. Finally, to determine whether SNPs were associated with potential risk factors, we searched all SNPs in PhenoScanner (Version 2, http://www.phenoscanner.medschl.cam.ac.uk/) [31, 32]. We removed SNPs associated with diseases or risk factors potentially associated with brain structural changes, including all neurological and psychiatric disorders, fluid intelligence, obesity, hypoxemia, and other potential confounders [33, 34]. The remaining SNPs were used in the MR analysis.

### Statistical analysis

To address variant heterogeneity and pleiotropy, we used three different MR methods: (1) inverse-variance weighted (IVW), which was the main analysis method and (2) the weighted median and MR-Egger methods in sensitivity analyses to improve the IVW model-based estimation. The IVW method yields high-power results but is based on the premise that all IVs were valid [35]. The weighted median approach provides consistent effect estimates when < 50% of the genetic variants are invalid [36], whereas the MR-Egger method provided estimates after correcting for pleiotropic effects, although at the cost of lower statistical power [37]. The effect estimates were considered significant only when *p*_*IVW*_ < 0.05, and all methods had consistent *β* directions [38]. For the significance estimates, the MR-PRESSO global test and MR-Egger regression test were used as the main methods to account for potential pleiotropy [39, 40]. Additionally, Cochran’s *Q* statistic was used to evaluate heterogeneity among genetic variants [41]. A *p* value less than 1.07 × 10^−4^ (0.05/468, Bonferroni method) was considered statistically significant, while a *p* value less than 0.05 was considered nominally significant evidence for a potential causal association [42, 43]. All statistical analyses were performed using RStudio (R version 4.1.1) with the packages “TwoSampleMR” [44] and “MR-PRESSO” [40].

## Results

**Fig. 2:**
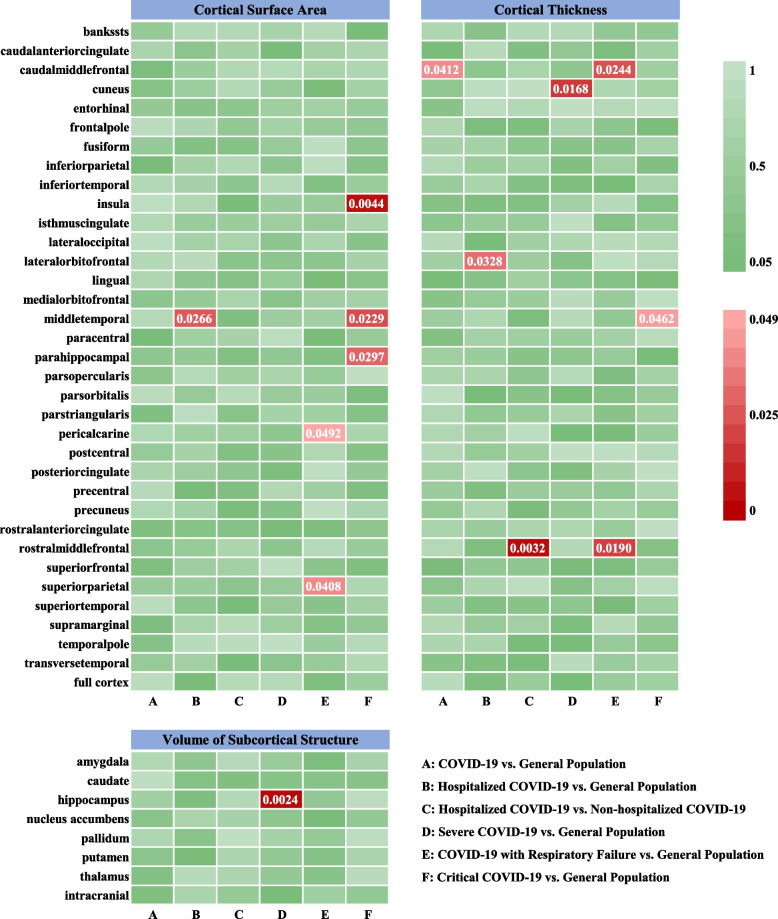
IVW estimates of the effect of COVID-19 on brain structures. The color of each block represents the IVW-derived *p* values of each MR analysis. *p* < 0.05 is shown in red and *p* ≥ 0.05 is shown in green. A *p* value < 1.07 × 10^−4^ was considered statistically significant. A *p* value < 0.05 was considered nominally significant

**Fig. 3:**
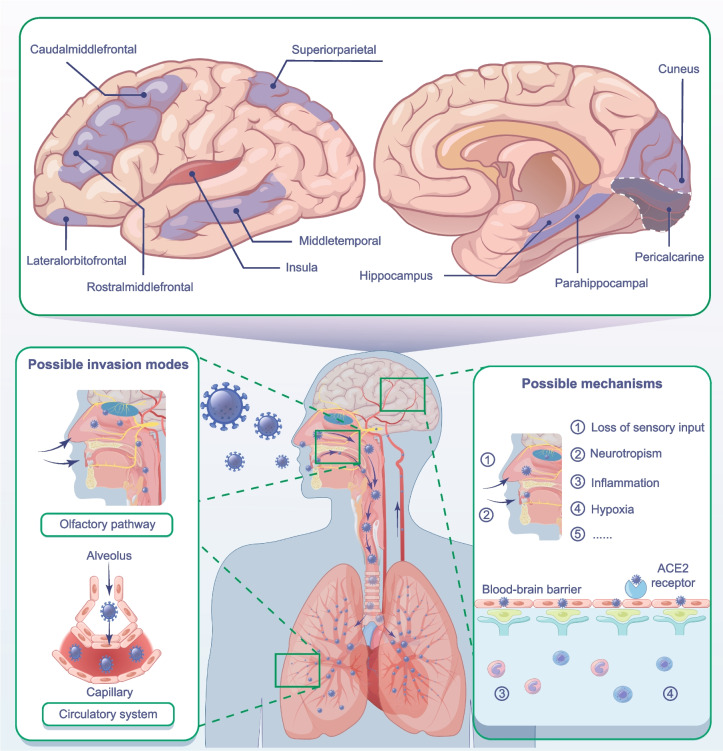
The two-sample MR framework showed that COVID-19 potentially causes structural changes in specific brain functional regions. Brain regions with positive IVW-derived *β* values are shown in red and brain regions with negative IVW-derived *β* values are shown in purple

MR analysis was performed to determine whether there were causal relationships of COVID-19 with cortical thickness, cortical surface area, or volume of subcortical structures (Fig. 2). Detailed results are presented in Additional file 1: Table S2-S7. We identified some nominally significant brain structures affected by COVID-19 (Table 1, Fig. 3). The characteristics of selected SNPs are presented in Additional file 1: Table S8. A low risk of substantial weak instrument bias was identified, as the *F* statistics for all the SNPs ranged from 28 to 50,488. No SNPs were associated with neurological diseases or hypoxemia according to the PhenoScanner. rs332040 was significantly associated with psychiatric disorders, including worry or anxiety (*p* = 5.28 × 10^−22^), neuroticism (*p* = 1.09 × 10^−14^), and miserableness (*p* = 6.27 × 10^−9^). rs17707300 was associated with fluid intelligence (*p* = 1.35 × 10^−10^) and body mass index (*p* = 3.28 × 10^−25^). The remaining SNPs were not directly associated with brain structures and related confounders.

**Table 1:** Main results of the MR analysis

**Exposures** Outcomes	**nSNPs**	**Method**	***β* (95%CI)**	**SE**	***p* value**
**COVID-19 vs. general population**					
Thickness of caudal middle frontal gyrus	28	IVW	− 0.0044 (− 0.0087, − 0.0002)	0.0022	0.0412
**Hospitalized COVID-19 vs. general population**					
Thickness of lateral orbitofrontal gyrus	26	IVW	− 0.0049 (− 0.0093, − 0.0004)	0.0023	0.0328
Surface area of middle temporal gyrus	26	IVW	− 10.8855 (− 20.5067, − 1.2642)	4.9088	0.0266
**Hospitalized COVID-19 vs. non-hospitalized COVID-19**					
Thickness of rostral middle frontal gyrus	20	IVW	− 0.0022 (− 0.0037, − 0.0008)	0.0008	0.0032
**Severe COVID-19 vs. general population**					
Thickness of cuneus	39	IVW	− 0.0024 (− 0.0043, − 0.0004)	0.0010	0.0168
Volume of hippocampus	36	IVW	− 15.9127 (− 26.2067, − 5.6188)	5.2520	0.0024
**Severe COVID-19 with respiratory failure vs. general population**					
Thickness of rostral middle frontal gyrus	14	IVW	− 0.0014 (− 0.0025, − 0.0002)	0.0006	0.0190
Thickness of caudal middle frontal gyrus	14	IVW	− 0.0017 (− 0.0032, − 0.0002)	0.0008	0.0244
Surface area of pericalcarine	14	IVW	− 2.6628 (− 5.3167, − 0.0089)	1.3540	0.0492
Surface area of superior parietal gyrus	14	IVW	− 5.6310 (− 11.0270, − 0.2348)	2.7532	0.0408
**Critical COVID-19 vs. general population**					
Thickness of middle temporal gyrus	92	IVW	− 0.0002 (− 0.0003, 0)	0.0001	0.0462
Surface area of middle temporal gyrus	92	IVW	− 0.5261 (− 0.9793, − 0.0729)	0.2312	0.0229
Surface area of parahippocampal gyrus	92	IVW	− 0.1473 (− 0.2802, − 0.0145)	0.0678	0.0297
Surface area of insula	92	IVW	0.3832 (0.1192, 0.6472)	0.1347	0.0044

*Abbreviations*: *COVID-19* Coronavirus disease 2019, *CI* Confidence interval, *IVW* inverse-variance weighted, *nSNPs* Number of single-nucleotide polymorphisms, *SE* Standard error. The *β* values were calculated in millimeters. A *p* value < 1.07×10^−4^ (Bonferroni-corrected significance threshold) was considered statistically significant. A *p* value < 0.05 was considered nominally significant

### Causal estimates of genetically predicted COVID-19 on cortical thickness

The genetically predicted COVID-19 infection phenotype was nominally associated with reduced cortical thickness in the caudal middle frontal gyrus (*β* = − 0.0044 mm, SE = 0.0022, *p* = 0.0412). A similar result was obtained when analyzing a population with a severe COVID-19 phenotype (*β* = − 0.0017 mm, SE = 0.0008, *p* = 0.0244). The hospitalized COVID-19 phenotype was nominally associated with reduced cortical thickness in the lateral orbitofrontal cortex (*β* = − 0.0049 mm, SE = 0.0023, *p* = 0.0328) and rostral middle frontal gyrus (*β* = − 0.0022 mm, SE = 0.0008, *p* = 0.0032). A similar association was found between reduced cortical thickness in the rostral middle frontal gyrus and the severe COVID-19 phenotype (*β* = − 0.0014 mm, SE = 0.0006, *p* = 0.0190). Genetic predisposition to the severe COVID-19 phenotype was also nominally associated with decreased cortical thickness in the cuneus (*β* = − 0.0024 mm, SE = 0.0010, *p* = 0.0168) and middle temporal gyrus (*β* = − 0.0002 mm, SE = 0.0001, *p* = 0.0462) (Table 1).

### Causal estimates of genetically predicted COVID-19 on cortical surface area

The genetically predicted COVID-19 infection phenotype had no causal effect on the cortical surface area. However, the hospitalized COVID-19 phenotype was nominally associated with reduced surface area of the middle temporal gyrus (*β* = − 10.8855 mm^2^, SE = 4.9088, *p* = 0.0266), and this causal effect was also confirmed in the severe COVID-19 phenotype (*β* = − 0.5261 mm^2^, SE = 0.2312, *p* = 0.0229). In addition, genetic predisposition to severe COVID-19 was nominally associated with decreased surface area in several cortices, including the parahippocampal gyrus (*β* = − 0.1473 mm^2^, SE = 0.0678, *p* = 0.0297), pericalcarine (*β* = − 2.6628 mm^2^, SE = 1.3540, *p* = 0.0492), and superior parietal cortex (*β* = − 5.6310 mm^2^, SE = 2.7532, *p* = 0.0408). Notably, the severe COVID-19 phenotype was nominally associated with an increased cortical surface area of the insula (*β* = 0.3832 mm^2^, SE = 0.1347, *p* = 0.0044) (Table 1).

### Causal estimates of genetically predicted COVID-19 on the volume of subcortical structures

There was nominally significant evidence that the severe COVID-19 phenotype was associated with reduced volume of the hippocampus (*β* = − 15.9127 mm^3^, SE = 5.2520, *p* = 0.0024). The weighted median approach showed a similar effect size (*β* = − 15.8282 mm^3^, SE = 7.8174) and significance level (*p* = 0.0429). However, no statistically significant associations of subcortical structures with the COVID-19 infection phenotype or the hospitalized COVID-19 phenotype were identified (Table 1).

### Sensitivity analysis

Sensitivity analyses were performed using MR-Egger and weighted median analyses. All of these results were directionally consistent with the IVW analyses. For the aforementioned effect estimates, we also used Cochran’s *Q* statistic to evaluate the degree of heterogeneity, as well as the MR-PRESSO global test and MR-Egger intercept test to detect horizontal pleiotropy. Heterogeneity was detected regarding the surface area of the middle temporal gyrus (*p*_*Q*_ = 0.0010) and parahippocampal gyrus (*p*_*Q*_ = 0.0199). MR-PRESSO global tests showed horizontal pleiotropy in the surface area of the middle temporal gyrus and parahippocampal gyrus as well as in cortical thickness of the caudal middle frontal gyrus, but no horizontal pleiotropy was identified by the MR-Egger intercept test. The results of sensitivity analyses are shown in Table 2.

**Table 2:** Heterogeneity and pleiotropy tests of the causal effects of COVID-19 on brain structures

**Exposures**	**Cochrane’s *Q* test**		**MR-Egger intercept test**		**MRPRESSO global test *p* value**
Outcomes	***Q*-value**	***p***_***Q***_	**Intercept**	***p***_**intercept**_	
**COVID-19 vs. general population**					
Thickness of caudal middle frontal gyrus	36.5379	0.1040	− 0.0003	0.5605	0.0470
**Hospitalized COVID-19 vs. general population**					
Thickness of lateral orbitofrontal gyrus	22.6627	0.5973	− 0.0002	0.7648	0.5780
Surface area of middle temporal gyrus	25.8789	0.4141	− 0.8634	0.5589	0.4400
**Hospitalized COVID-19 vs. non-hospitalized COVID-19**					
Thickness of rostral middle frontal gyrus	15.6886	0.6779	− 0.0002	0.7609	0.6830
**Severe COVID-19 vs. general population**					
Thickness of cuneus	43.8548	0.2371	0.0006	0.2634	0.2640
Volume of hippocampus	34.0941	0.5117	− 3.1943	0.2435	0.5160
**Severe COVID-19 with respiratory failure vs. general population**					
Thickness of rostral middle frontal gyrus	6.7957	0.9124	0	0.9628	0.8080
Thickness of caudal middle frontal gyrus	16.9137	0.2033	− 0.0005	0.5768	0.2750
Surface area of pericalcarine	7.5854	0.8695	1.6803	0.2526	0.9240
Surface area of superior parietal gyrus	10.6660	0.6393	1.6369	0.5755	0.7390
**Critical COVID-19 vs. general population**					
Thickness of middle temporal gyrus	76.1485	0.8680	0.0001	0.6839	0.8910
Surface area of middle temporal gyrus	138.3960	0.0010	1.1069	0.2578	0.0010
Surface area of parahippocampal gyrus	120.8511	0.0199	0.2390	0.4051	0.0130
Surface area of insula	96.8868	0.3169	0.0544	0.9242	0.3400

*Abbreviations*: *COVID-19* Coronavirus disease 2019, *MR-PRESSO* Mendelian randomization-pleiotropy residual sum and outlier

## Discussion

Using two-sample MR analysis, our study identified the potential causal effects of COVID-19 on changes in cortical and subcortical structures, providing new evidence for a causal relationship between COVID-19 and brain structural changes. Moreover, we identified specific brain functional regions vulnerable to COVID-19 among 34 cortical regions and seven subcortical structures. According to our results, genetically predicted COVID-19 phenotypes are nominally associated with atrophy in specific brain functional regions, including the lateral orbitofrontal cortex, parahippocampal gyrus, hippocampus, pericalcarine, cuneus, middle frontal gyrus, middle temporal gyrus, and superior parietal cortex. More severe COVID-19 might be associated with more extensive brain atrophy.

A study published recently reported that an increased cortical thickness in the left inferior temporal gyrus may increase susceptibility to and hospitalization with COVID-19 [45]. In contrast, our study aimed to investigate the effects of COVID-19 on brain structures and identify specific brain functional regions that are vulnerable to COVID-19. Our findings shed light on the patterns and mechanisms of brain damage caused by COVID-19. Based on large-scale GWAS data, our study confirmed the results of previous studies [5, 46]. Douai et al. found that COVID-19 was related to atrophy in brain regions functionally connected to the primary olfactory cortex, including the orbitofrontal cortex, hippocampus, parahippocampal gyrus, and insula [5]. Consistent with these findings, our study identified this causal relationship using MR analysis. Additionally, a recent follow-up study showed that patients with severe COVID-19 have a greater variety and a higher incidence of neuropsychiatric symptoms [3], which may indicate more extensive brain damage. According to our results, brain atrophy might be confined to some regions in the frontotemporal cortex in population with relatively mild COVID-19, whereas severe COVID-19 could affect more brain regions, including the hippocampus and par hippocampal gyrus, as well as some parietal and occipital cortices, indicating that the range of brain damage may be related to the severity of COVID-19.

COVID-19 potentially causes atrophy in the orbitofrontal cortex, hippocampus, parahippocampal gyrus, and insula, which are olfactory-related brain regions. At the level of brain networks and neurocognitive functions, these olfactory-related brain regions are closely associated with olfactory perception, memory, and emotion regulation. The orbitofrontal cortex, as the secondary olfactory cortex, plays an important role in the olfactory pathway [47]. COVID-19 might affect olfactory perception through the orbitofrontal cortex [16, 48, 49]. As this brain region is the core of the olfaction-emotion neural circuit, abnormal activation could lead to cacosmia and might further cause anxiety and depression in patients with COVID-19 [50, 51]. The orbitofrontal cortex also plays a critical role in emotion regulation and cognitive functions, such as depression, decision-making, attention, and executive function [52–55], which are also impaired in patients with COVID-19 [56–59]. At the level of histopathology and cell biology, SARS-CoV-2 was shown to infect astrocytes and lead to neuronal death in the orbitofrontal cortex in individuals with COVID-19 [60]. Additionally, a large body of evidence has shown that SARS-CoV-2 targets cells expressing angiotensin-converting enzyme 2 (ACE2) receptors via spike glycoprotein with the assistance of transmembrane protein serine protease 2 (TMPRSS2) [8]. Although ACE2 and TMPRSS2 are expressed in the brain tissue at low levels, they are highly expressed in supporting cells in the olfactory epithelium, where SARS-CoV-2 invasion or virus-induced neuroinflammation may serve as a port of entry to the central nervous system [8–10]. Additionally, the hippocampus is the hub of brain regions dealing with memory [61]; connectivity between the hippocampus and insula links the hippocampus to the salience network and default mode network, which is crucial for memory processing and consolidation [62].

Memory encoding, storage, and retrieval depend on the integrity and coordination of the hippocampus, parahippocampal gyrus, and prefrontal cortex [61]. Previous research has shown that SARS-CoV-2 infection could induce neuroinflammation, cause neuronal degeneration, and inhibit neurogenesis in the hippocampus of humans and hamsters without SARS-CoV-2 invasion [63, 64]. The causal effects of COVID-19 on atrophy in the hippocampus and parahippocampal gyrus might be related to the impairment of episodic memory and working memory after COVID-19 [7, 65, 66]. The insula is an anatomical integration hub with tight connectivity to extensive networks that can compensatively enhance functional connectivity with other brain regions, especially the hippocampus, in normal elderly patients and in patients with mild cognitive impairment [67, 68]. Based on neural adaptation [69], the positive *β* value of the insula in our results might indicate the existence of a compensatory mechanism to mitigate the negative effects of COVID-19 on other brain functional regions. Our findings, combined with previous neuroimaging, cognitive neuroscience, histopathological, cellular, and molecular evidence, further indicate that olfactory-related brain regions are the brain structures predominantly affected by COVID-19, which suggests possible causes of cognitive impairment after COVID-19. The underlying mechanisms may include SARS-CoV-2 invasion via the olfactory pathway, loss of olfactory sensory input, and virus-induced neuroinflammation.

According to previous studies, visual impairment in patients with COVID-19 is usually considered a consequence of conjunctivitis, retinitis, central retinal artery/venous occlusion, or retinal bleeding [70]. The unexplained retinal microstructural changes may result from retinal vascular disease or virus-induced inflammation [70–72]. However, our study is the first to find that COVID-19 might cause atrophy in the pericalcarine and cuneus, suggesting vulnerability of the visual-related cortex to COVID-19. The pericalcarine is the primary visual cortex, whereas the processing of visual signals requires the involvement of the cuneus, which is activated almost simultaneously with the primary visual cortex in response to visual stimuli [73]. The higher level of ACE2 expressed in the visual-related cortex compared with that in other brain regions probably potentiates SARS-CoV-2 infection [74, 75]. The high expression levels of ACE2 in vascular endothelial cells suggest that SARS-CoV-2 might cross the blood-brain barrier and invade brain tissue [12], especially the visual-related cortex. The mechanisms leading to impairment of the visual-related cortex and visual perception in patients with COVID-19 remain unclear. We hypothesize that the invasion of SARS-CoV-2 across the blood-brain barrier into the visual-related cortex or the attenuation of visual sensory input due to visual impairment may explain these phenomena.

Our study also indicates a suggestively significant association between severe COVID-19 and atrophy in the superior parietal cortex, middle frontal gyrus, and middle temporal gyrus. Structural abnormalities in the frontal, parietal, and temporal lobes have been reported in previous studies of patients with COVID-19, especially in severe cases [76, 77]. The superior parietal cortex is an important component of the dorsal attentional network, and abnormality in this region might be associated with impaired attention and visuospatial processing in patients with COVID-19 [78, 79]. In addition, previous studies have shown reduced gray matter volume in the middle frontal gyrus of patients with COVID-19 requiring oxygen therapy and in the middle temporal gyrus of patients with COVID-19 who are febrile [80]. A recent randomized controlled trial showed that at least 3 months of hyperbaric oxygen therapy after COVID-19 improved patients’ neurocognitive symptoms and the microstructure of some gyrus in the frontal, parietal, and temporal cortices. Therefore, hypoxemia may mediate the causal effects of COVID-19 on brain structural changes [8]. However, the precise mechanism requires further study.

Our study found that different COVID-19 phenotypes result in structural changes in different brain regions. Pathological processes secondary to severe COVID-19, such as hypoxemia and shock, may mediate the causal effects of COVID-19 on brain structural changes, thereby complicating the mechanisms by which COVID-19 damages brain structures. Different secondary pathological processes may impact different brain regions [8]. Additionally, different COVID-19 phenotypes are associated with different genetic predispositions. Patients with a genetic predisposition to severe COVID-19 may exhibit different patterns of altered brain regions than patients with mild COVID-19. Based on our findings, we suggest that severe COVID-19 tends to affect a broader range of brain regions, causing damage to evolutionarily older brain regions, such as the hippocampus and parahippocampal gyrus [81]. Nonetheless, further research is necessary to understand the underlying mechanisms.

The deleterious effects of COVID-19 on brain structures suggest that the residual cognitive impairments and mental disorders experienced by patients may be irreversible, which would diminish the quality of life, especially in the elderly population. The increased risk of Alzheimer’s disease and related dementias is considered a long-term consequence of SARS-CoV-2 infection [82], particularly in patients with severe COVID-19 [83]. Thus, patients with COVID-19 and cognitive impairment should be actively managed to alleviate neurocognitive symptoms and minimize long-term effects.

## Strengths and limitations

The primary strength of our study is the MR design, which can overcome misinterpretation of a causal relationship resulting from reverse causality or potential confounders [84]. We removed SNPs associated with psychiatric disorders, fluid intelligence, and body mass index, which reduces concerns that brain structural changes were caused by other health conditions. By analyzing large-scale GWAS data related to COVID-19 phenotypes, 34 cortical regions, and seven subcortical structures, we provide more detailed information regarding specific brain functional regions vulnerable to COVID-19. Our study also has some limitations. First, it is important to note that the severity of brain structural changes in our framework was not investigated. More specific analyses are needed to determine the causal relationship between the severity of brain structural changes and different COVID-19 phenotypes. Second, although brain morphological indicators in our study characterized structural changes, they are not the optimal indicators for investigating neurological function and underlying mechanisms. We also cannot fully determine whether pathological processes secondary to COVID-19 mediate the causal effects of COVID-19 on brain structural changes. Thus, future studies should investigate the mechanisms by which COVID-19 affects neurological function and brain structures. Third, SNPs were selected using a relatively relaxed threshold (*p* < 1 × 10^−5^), and the *p* values of estimates were only nominally significant, which may reduce the credibility of the results to some extent. Fourth, selection bias would exist due to the binary exposures used in this MR analysis. The collider bias “COVID-19 infection” may be present when analyzing the phenotype “hospitalized COVID-19 versus non-hospitalized COVID-19”. Fifth, a relatively relaxed threshold (*r*^2^ > 0.8) was used when SNPs were not available in the outcome dataset, which may cause pleiotropy bias. Sixth, the nominally significant findings in our study need to be confirmed by further research. Moreover, some effect values in our study are very small, which may limit their practical application and require further research. Finally, the proportion of cases included in this study was small and the participants were of European ancestry who were diagnosed with COVID-19 in 2020, when the predominant variant of SARS-CoV-2 was the wild type. Hence, our exploratory findings should be interpreted with caution in populations with non-European ancestry or regarding other SARS-CoV-2 variants.

## Conclusions

In conclusion, we found a suggestively significant association between genetic predisposition to COVID-19 and atrophy in specific functional regions of the human brain by MR analysis of large-scale GWAS data. More extensive brain atrophy may result from severe COVID-19. Irrespective of the exact mechanism of the associations via a genetic background, our findings provide new evidence for a causal relationship between COVID-19 and brain structural changes and suggest possible causes of cognitive impairment after COVID-19.

## Supplementary Information

**Additional file 1:** **TableS1.** STROBE-MR reporting guidelines. **Table S2.** MR analysis of the causal relationship between COVID-19and brain structure. **Table S3.** MRanalysis of the causal relationship between hospitalized COVID-19 and brainstructure. **Table S4.** MR analysis ofthe causal relationship between hospitalized COVID-19and brain structure. **Table S5.**MR analysis of the causal relationship between severe COVID-19 and brainstructure. **Table S6.** MR analysis ofthe causal relationship between severe COVID-19 with respiratory failure andbrain structure. **Table S7.** MRanalysis of the causal relationship between critical COVID-19 and brainstructure. **Table S8.** Characteristicsof selected SNPs for COVID-19 phenotypes.

## Data Availability

The data used in this study is publicly available for download in the ENIGMA Consortium (https://enigma.ini.usc.edu/) and COVID-19 Host Genetics Initiative (https://www.covid19hg.org/).

## Abbreviations

ACE2: Angiotensin-converting enzyme 2
COVID-19: Coronavirus disease 2019
GWAS: Genome-wide association study
IVW: Inverse-variance weighted
IVs: Instrumental variables
LD: Linkage disequilibrium
MRI: Magnetic resonance imaging
MR: Mendelian randomization
PET: Positron emission tomography
SARS-CoV-2: Severe acute respiratory syndrome coronavirus 2
SNPs: Single-nucleotide polymorphisms
STROBE: Strengthening the Reporting of Observational Studies in Epidemiology
TMPRSS2: Transmembrane protein serine protease 2
