# Supplementary material for: Causal effects of COVID-19 on structural changes in specific brain regions: a Mendelian randomization study
Source: BMC Med. 2023 Jul 19;21:261. doi: 10.1186/s12916-023-02952-1 (PMC10354891; doi:10.1186/s12916-023-02952-1)
Supplement: Supplementary file 1 — Additional file 1: TableS1. STROBE-MR reporting guidelines. Table S2. MR analysis of the causal relationship between COVID-19and brain structure. Table S3. MRanalysis of the causal relationship between hospitalized COVID-19 and brainstructure. Table S4. MR analysis ofthe causal relationship between hospitalized COVID-19and brain structure. Table S5.MR analysis of the causal relationship between severe COVID-19 and brainstructure. Table S6. MR analysis ofthe causal relationship between severe COVID-19 with respiratory failure andbrain structure. Table S7. MRanalysis of the causal relationship between critical COVID-19 and brainstructure. Table S8. Characteristicsof selected SNPs for COVID-19 phenotypes. [file 12916_2023_2952_MOESM1_ESM.pdf]

Additional File 1

Table S1. STROBE-MR reporting guidelines....1

Table S2. MR analysis of the causal relationship between COVID-19 and brain structure.....3

Table S3. MR analysis of the causal relationship between hospitalized COVID-19 and brain structure....11

Table S4. MR analysis of the causal relationship between hospitalized COVID-19 (vs. non-hospitalized COVID-19) and brain structure....19

Table S5. MR analysis of the causal relationship between severe COVID-19 and brain structure....27

Table S6. MR analysis of the causal relationship between severe COVID-19 with respiratory failure and brain structure....35

Table S7. MR analysis of the causal relationship between critical COVID-19 and brain structure....43

Table S8. Characteristics of selected SNPs for COVID-19 phenotypes....51

Table S1. STROBE-MR reporting guidelines

Relevant section	Item	Instruction	Location addressed
TITLE and ABSTRACT	TITLE and ABSTRACT	Indicate Mendelian randomization as the study's design in the title and/or the abstract.	Title and abstract
INTRODUCTION	Background	Explain the scientific background and rationale for the reported study. Is causality between exposure and outcome plausible? Justify why MR is a helpful method to address the study question.	Introduction
	Objectives	State specific objectives clearly, including pre-specified causal hypotheses (if any).	Introduction
METHODS	Study design and data sources	Present key elements of study design early in the paper.<br>Consider including a table listing sources of data for all phases of the study. For each data source contributing to the analysis, describe the following:<br>a) Describe the study design and the underlying population from which it was drawn. Describe also the setting, locations, and relevant dates, including periods of recruitment, exposure, follow-up, and data collection, if available.<br>b) Give the eligibility criteria, and the sources and methods of selection of participants.<br>c) Explain how the analyzed sample size was arrived at.<br>d) Describe measurement, quality and selection of genetic variants.<br>e) For each exposure, outcome and other relevant variables, describe methods of assessment and, in the case of diseases, the diagnostic criteria used.<br>f) Provide details of ethics committee approval and participant informed consent, if relevant.	a) Abstract, Introduction, Methods, Figure 1, Supplementary Tables<br>b) Methods<br>c) Methods<br>d) Methods<br>e) Methods<br>f) Methods
	Assumptions	Explicitly state assumptions for the main analysis (e.g. relevance, exclusion, independence, homogeneity) as well assumptions for any additional or sensitivity analysis.	Methods, Figure 1
	Statistical methods: main analysis	Describe statistical methods and statistics used.<br>a) Describe how quantitative variables were handled in the analyses (i.e., scale, units, model).<br>b) Describe the process for identifying genetic variants and weights to be included in the analyses (i.e, independence and model). Consider a flow diagram.<br>c) Describe the MR estimator, e.g. two-stage least squares, Wald ratio, and related statistics.Detail the included covariates and, in case of two-sample MR, whether the same covariate set was used for adjustment in the two samples.<br>d) Explain how missing data were addressed.<br>e) If applicable, say how multiple testing was dealt with	a) Methods<br>b) Methods<br>c) Methods<br>d) Methods<br>e) Methods
	Assessment of assumptions	Describe any methods used to assess the assumptions or justify their validity	Methods
	Sensitivity analyses	Describe any sensitivity analyses or additional analyses performed.	Methods
	Software and pre-registration	a) Name statistical software and package(s), including version and settings used;<br>b) State whether the study protocol and details were pre-registered (as well as when and where).	Methods
RESULTS	Descriptive data	a) Report the numbers of individuals at each stage of included studies and reasons for exclusion. Consider use of a flowdiagram.<br>b) Report summary statistics for phenotypic exposure(s), outcome(s) and other relevant variables (e.g. means, standard deviations, proportions).<br>c) If the data sources include meta-analyses of previous studies, provide the number of studies, their reported ancestry, if available, and assessments of heterogeneity across these studies. Consider using a supplementary table for each data source.<br>d) For two-sample Mendelian randomization:<br>i. Provide information on the similarity of the genetic variant exposure associations between the exposure and outcome samples.<br>ii. Provide information on extent of sample overlap between the exposure and outcome data sources	a) Methods, Figure 1, Supplementary Tables<br>b) Supplementary Tables<br>c) N/A<br>d) Methods, eMethods<br>i. N/A<br>ii. N/A

continued:

Relevant section	Item	Instruction	Location addressed
	Main results	a) Report the associations between genetic variant and exposure, and between genetic variant and outcome, preferably on an interpretable scale (e.g. comparing 25th and 75th percentile of allele count or genetic risk score, if individual-level data available).<br>b) Report causal effect estimate between exposure and outcome, and the measures of uncertainty from the MR analysis. Use an intuitive scale, such as odds ratio, or relative risk, per standard deviation difference.<br>c) If relevant, consider translating estimates of relative risk into absolute risk for a meaningful time-period.<br>d) Consider any plots to visualize results (e.g. forest plot, scatterplot of associations between genetic variants and outcome versus between genetic variants and exposure)	a) N/A, no individual level data available<br>b) Results, Supplementary Tables<br>c) N/A<br>d) N/A
	Assessment of assumptions	a) Assess the validity of the assumptions.<br>b) Report any additional statistics (e.g., assessments of heterogeneity, such as I2, Q statistic)	a) Methods, Results, Supplementary Tables<br>b) Results, Table 2.
	Sensitivity and additional analyses	a) Use sensitivity analyses to assess the robustness of the main results to violations of the assumptions.<br>b) Report results from other sensitivity analyses (e.g., replication study with different dataset, analyses of subgroups, validation of instrument(s), simulations, etc.).<br>c) Report any assessment of direction of causality (e.g., bidirectional MR).<br>d) When relevant, report and compare with estimates from nonMR analyses.<br>e) Consider any additional plots to visualize results (e.g., leaveone-out analyses).	a) Results<br>b) Results<br>c) Results, Tables 2.<br>d) Introduction, Discussion<br>e) N/A
DISCUSSION	Key results	Summarize key results with reference to study objectives.	Discussion
	Limitations	Discuss limitations of the study, taking into account the validity of the MR assumptions, other sources of potential bias, and imprecision. Discuss both direction and magnitude of any potential bias, and any efforts to address them.	Methods, Discussion
	Interpretation	a) Give a cautious overall interpretation of results considering objectives and limitations. Compare with results from other relevant studies.<br>b) Discuss underlying biological mechanisms that could be modelled by using the genetic variants to assess the relationship between the exposure and the outcome.<br>c) Discuss whether the results have clinical or policy relevance, and whether interventions could have the same size effect.	a) Discussion<br>b) Discussion<br>c) Abstract, Discussion
	Generalizability	Discuss the generalizability of the study results (a) to other populations (i.e. external validity), (b) across other exposure periods/timings, and (c) across other levels of exposure.	a) Discussion<br>b) Discussion<br>c) Discussion
OTHER INFORMATION	Funding	Give the source of funding and the role of the funders for the present study and, if applicable, for the original study or studies on which the present article is based.	Acknowledgements
	Data and data sharing	Present data used to perform all analyses or report where and how the data can be accessed. State whether statistical code is publicly accessible and if so, where.	Methods, Supplementary Tables, References, Data Sharing Statement
	Conflicts of Interest	All authors should declare all potential conflicts of interest.	Declaration of Competing Interest

Table S2. MR analysis of the causal relationship between COVID-19 and brain structure

COVID-19 phenotype	Brain structure	Method	nsnp	β	se	p value	β_low95%CI	β_up95%CI
COVID-19 vs. general population	Mean_bankssts_surface_area	MR Egger	28	0.6297	5.2218	0.9049	-9.6050	10.8644
COVID-19 vs. general population	Mean_bankssts_surface_area	Weighted median	28	3.8220	3.0430	0.2091	-2.1423	9.7862
COVID-19 vs. general population	Mean_bankssts_surface_area	Inverse variance weighted	28	1.7253	2.1458	0.4214	-2.4805	5.9312
COVID-19 vs. general population	Mean_bankssts_thickness	MR Egger	28	-0.0002	0.0067	0.9772	-0.0133	0.0129
COVID-19 vs. general population	Mean_bankssts_thickness	Weighted median	28	-0.0042	0.0035	0.2298	-0.0112	0.0027
COVID-19 vs. general population	Mean_bankssts_thickness	Inverse variance weighted	28	0.0008	0.0027	0.7641	-0.0045	0.0061
COVID-19 vs. general population	Mean_caudalanteriorcingulate_surface_area	MR Egger	28	-1.9256	5.7930	0.7422	-13.2799	9.4286
COVID-19 vs. general population	Mean_caudalanteriorcingulate_surface_area	Weighted median	28	-0.5770	2.9006	0.8423	-6.2622	5.1083
COVID-19 vs. general population	Mean_caudalanteriorcingulate_surface_area	Inverse variance weighted	28	-0.8158	2.3393	0.7273	-5.4009	3.7693
COVID-19 vs. general population	Mean_caudalanteriorcingulate_thickness	MR Egger	28	0.0039	0.0095	0.6840	-0.0147	0.0226
COVID-19 vs. general population	Mean_caudalanteriorcingulate_thickness	Weighted median	28	-0.0005	0.0056	0.9260	-0.0115	0.0105
COVID-19 vs. general population	Mean_caudalanteriorcingulate_thickness	Inverse variance weighted	28	-0.0079	0.0039	0.0426	-0.0156	-0.0003
COVID-19 vs. general population	Mean_caudalmiddlefrontal_surface_area	MR Egger	28	-2.8150	12.2660	0.8203	-26.8563	21.2263
COVID-19 vs. general population	Mean_caudalmiddlefrontal_surface_area	Weighted median	28	-12.4453	7.3872	0.0920	-26.9242	2.0336
COVID-19 vs. general population	Mean_caudalmiddlefrontal_surface_area	Inverse variance weighted	28	-8.4720	5.0438	0.0930	-18.3579	1.4139
COVID-19 vs. general population	Mean_caudalmiddlefrontal_thickness	MR Egger	28	-0.0015	0.0054	0.7769	-0.0121	0.0090
COVID-19 vs. general population	Mean_caudalmiddlefrontal_thickness	Weighted median	28	-0.0053	0.0029	0.0656	-0.0110	0.0003
COVID-19 vs. general population	Mean_caudalmiddlefrontal_thickness	Inverse variance weighted	28	-0.0044	0.0022	0.0412	-0.0087	-0.0002
COVID-19 vs. general population	Mean_cuneus_surface_area	MR Egger	28	3.7272	7.0577	0.6019	-10.1059	17.5602
COVID-19 vs. general population	Mean_cuneus_surface_area	Weighted median	28	3.3155	4.4360	0.4548	-5.3790	12.0099
COVID-19 vs. general population	Mean_cuneus_surface_area	Inverse variance weighted	28	3.4994	2.9024	0.2279	-2.1894	9.1882
COVID-19 vs. general population	Mean_cuneus_thickness	MR Egger	28	-0.0024	0.0052	0.6541	-0.0125	0.0078
COVID-19 vs. general population	Mean_cuneus_thickness	Weighted median	28	-0.0004	0.0032	0.9101	-0.0066	0.0059
COVID-19 vs. general population	Mean_cuneus_thickness	Inverse variance weighted	28	0.0020	0.0021	0.3541	-0.0022	0.0062
COVID-19 vs. general population	Mean_entorhinal_surface_area	MR Egger	28	-0.7332	3.1180	0.8159	-6.8445	5.3781
COVID-19 vs. general population	Mean_entorhinal_surface_area	Weighted median	28	1.3030	1.7700	0.4616	-2.1661	4.7721
COVID-19 vs. general population	Mean_entorhinal_surface_area	Inverse variance weighted	28	1.0386	1.2689	0.4131	-1.4485	3.5257
COVID-19 vs. general population	Mean_entorhinal_thickness	MR Egger	28	0.0153	0.0147	0.3070	-0.0135	0.0442
COVID-19 vs. general population	Mean_entorhinal_thickness	Weighted median	28	0.0056	0.0088	0.5264	-0.0117	0.0228
COVID-19 vs. general population	Mean_entorhinal_thickness	Inverse variance weighted	28	0.0070	0.0061	0.2446	-0.0048	0.0189
COVID-19 vs. general population	Mean_frontalpole_surface_area	MR Egger	28	-1.6188	1.3857	0.2533	-4.3347	1.0972
COVID-19 vs. general population	Mean_frontalpole_surface_area	Weighted median	28	-0.5727	0.8316	0.4910	-2.2026	1.0572

continued:

COVID-19 phenotype	Brain structure	Method	nsnp	β	se	p value	β_low95%CI	β_up95%CI
COVID-19 vs. general population	Mean_frontalpole_surface_area	Inverse variance weighted	28	-0.0489	0.5695	0.9315	-1.1651	1.0672
COVID-19 vs. general population	Mean_frontalpole_thickness	MR Egger	28	0.0066	0.0115	0.5698	-0.0159	0.0291
COVID-19 vs. general population	Mean_frontalpole_thickness	Weighted median	28	0.0053	0.0064	0.4069	-0.0073	0.0180
COVID-19 vs. general population	Mean_frontalpole_thickness	Inverse variance weighted	28	0.0013	0.0047	0.7747	-0.0078	0.0105
COVID-19 vs. general population	Mean_full_surface_area	MR Egger	4	839.3944	2870.4244	0.7975	-4786.6375	6465.4262
COVID-19 vs. general population	Mean_full_surface_area	Weighted median	4	112.8904	980.8444	0.9084	-1809.5645	2035.3454
COVID-19 vs. general population	Mean_full_surface_area	Inverse variance weighted	4	-74.5330	814.0176	0.9270	-1670.0075	1520.9414
COVID-19 vs. general population	Mean_full_thickness	MR Egger	8	0.0082	0.0093	0.4108	-0.0100	0.0263
COVID-19 vs. general population	Mean_full_thickness	Weighted median	8	0.0029	0.0049	0.5535	-0.0067	0.0125
COVID-19 vs. general population	Mean_full_thickness	Inverse variance weighted	8	-0.0006	0.0048	0.9028	-0.0100	0.0089
COVID-19 vs. general population	Mean_fusiform_surface_area	MR Egger	28	7.2426	13.5552	0.5977	-19.3256	33.8109
COVID-19 vs. general population	Mean_fusiform_surface_area	Weighted median	28	9.7472	7.0917	0.1693	-4.1525	23.6469
COVID-19 vs. general population	Mean_fusiform_surface_area	Inverse variance weighted	28	4.3220	5.4734	0.4297	-6.4059	15.0500
COVID-19 vs. general population	Mean_fusiform_thickness	MR Egger	28	-0.0053	0.0049	0.2931	-0.0148	0.0043
COVID-19 vs. general population	Mean_fusiform_thickness	Weighted median	28	0.0000	0.0031	0.9970	-0.0060	0.0060
COVID-19 vs. general population	Mean_fusiform_thickness	Inverse variance weighted	28	0.0009	0.0020	0.6671	-0.0031	0.0048
COVID-19 vs. general population	Mean_inferiorparietal_surface_area	MR Egger	28	11.0395	18.9827	0.6030	-26.1667	48.2457
COVID-19 vs. general population	Mean_inferiorparietal_surface_area	Weighted median	28	15.2466	12.1938	0.2092	-8.6533	39.1465
COVID-19 vs. general population	Mean_inferiorparietal_surface_area	Inverse variance weighted	28	16.9400	8.2049	0.0511	0.8585	33.0216
COVID-19 vs. general population	Mean_inferiorparietal_thickness	MR Egger	28	0.0018	0.0036	0.6240	-0.0052	0.0087
COVID-19 vs. general population	Mean_inferiorparietal_thickness	Weighted median	28	0.0012	0.0021	0.5469	-0.0028	0.0053
COVID-19 vs. general population	Mean_inferiorparietal_thickness	Inverse variance weighted	28	-0.0003	0.0015	0.8276	-0.0032	0.0025
COVID-19 vs. general population	Mean_inferiortemporal_surface_area	MR Egger	28	-27.3127	13.2569	0.0495	-53.2962	-1.3292
COVID-19 vs. general population	Mean_inferiortemporal_surface_area	Weighted median	28	-9.7069	7.6675	0.2055	-24.7352	5.3215
COVID-19 vs. general population	Mean_inferiortemporal_surface_area	Inverse variance weighted	28	1.2115	5.6846	0.8312	-9.9304	12.3534
COVID-19 vs. general population	Mean_inferiortemporal_thickness	MR Egger	28	-0.0075	0.0055	0.1814	-0.0183	0.0032
COVID-19 vs. general population	Mean_inferiortemporal_thickness	Weighted median	28	0.0000	0.0031	1.0000	-0.0062	0.0062
COVID-19 vs. general population	Mean_inferiortemporal_thickness	Inverse variance weighted	28	-0.0015	0.0022	0.4924	-0.0059	0.0028
COVID-19 vs. general population	Mean_insula_surface_area	MR Egger	28	6.1769	9.7632	0.5325	-12.9590	25.3129
COVID-19 vs. general population	Mean_insula_surface_area	Weighted median	28	3.6171	4.8179	0.4528	-5.8259	13.0601
COVID-19 vs. general population	Mean_insula_surface_area	Inverse variance weighted	28	0.0989	3.9772	0.9802	-7.6964	7.8941
COVID-19 vs. general population	Mean_insula_thickness	MR Egger	28	-0.0026	0.0058	0.6593	-0.0139	0.0087

continued:

COVID-19 phenotype	Brain structure	Method	nsnp	β	se	p value	β_low95%CI	β_up95%CI
COVID-19 vs. general population	Mean_insula_thickness	Weighted median	28	-0.0042	0.0034	0.2158	-0.0109	0.0025
COVID-19 vs. general population	Mean_insula_thickness	Inverse variance weighted	28	-0.0029	0.0024	0.2136	-0.0075	0.0017
COVID-19 vs. general population	Mean_isthmuscingulate_surface_area	MR Egger	28	0.7882	5.4062	0.8852	-9.8080	11.3844
COVID-19 vs. general population	Mean_isthmuscingulate_surface_area	Weighted median	28	2.1814	3.1706	0.4915	-4.0331	8.3958
COVID-19 vs. general population	Mean_isthmuscingulate_surface_area	Inverse variance weighted	28	0.5017	2.1964	0.8193	-3.8032	4.8066
COVID-19 vs. general population	Mean_isthmuscingulate_thickness	MR Egger	28	0.0003	0.0095	0.9767	-0.0184	0.0189
COVID-19 vs. general population	Mean_isthmuscingulate_thickness	Weighted median	28	-0.0054	0.0048	0.2647	-0.0148	0.0041
COVID-19 vs. general population	Mean_isthmuscingulate_thickness	Inverse variance weighted	28	-0.0038	0.0038	0.3206	-0.0113	0.0037
COVID-19 vs. general population	Mean_lateraloccipital_surface_area	MR Egger	28	24.2703	19.4307	0.2228	-13.8138	62.3545
COVID-19 vs. general population	Mean_lateraloccipital_surface_area	Weighted median	28	0.9109	10.6674	0.9319	-19.9972	21.8191
COVID-19 vs. general population	Mean_lateraloccipital_surface_area	Inverse variance weighted	28	0.3555	8.1128	0.9650	-15.5456	16.2566
COVID-19 vs. general population	Mean_lateraloccipital_thickness	MR Egger	28	-0.0054	0.0053	0.3132	-0.0157	0.0049
COVID-19 vs. general population	Mean_lateraloccipital_thickness	Weighted median	28	0.0011	0.0028	0.6952	-0.0044	0.0065
COVID-19 vs. general population	Mean_lateraloccipital_thickness	Inverse variance weighted	28	0.0003	0.0022	0.8757	-0.0039	0.0046
COVID-19 vs. general population	Mean_lateralorbitofrontal_surface_area	MR Egger	28	10.8829	9.7485	0.2745	-8.2241	29.9900
COVID-19 vs. general population	Mean_lateralorbitofrontal_surface_area	Weighted median	28	1.6253	5.3265	0.7603	-8.8146	12.0652
COVID-19 vs. general population	Mean_lateralorbitofrontal_surface_area	Inverse variance weighted	28	-0.9002	4.0653	0.8247	-8.8682	7.0677
COVID-19 vs. general population	Mean_lateralorbitofrontal_thickness	MR Egger	28	-0.0056	0.0060	0.3579	-0.0172	0.0061
COVID-19 vs. general population	Mean_lateralorbitofrontal_thickness	Weighted median	28	-0.0004	0.0032	0.9054	-0.0066	0.0058
COVID-19 vs. general population	Mean_lateralorbitofrontal_thickness	Inverse variance weighted	28	-0.0013	0.0024	0.5858	-0.0060	0.0034
COVID-19 vs. general population	Mean_lingual_surface_area	MR Egger	28	-6.2133	19.1514	0.7482	-43.7501	31.3235
COVID-19 vs. general population	Mean_lingual_surface_area	Weighted median	28	-5.5158	9.3455	0.5551	-23.8330	12.8014
COVID-19 vs. general population	Mean_lingual_surface_area	Inverse variance weighted	28	2.1885	7.7635	0.7780	-13.0280	17.4049
COVID-19 vs. general population	Mean_lingual_thickness	MR Egger	28	0.0004	0.0050	0.9085	-0.0094	0.0103
COVID-19 vs. general population	Mean_lingual_thickness	Weighted median	28	0.0010	0.0027	0.7148	-0.0043	0.0063
COVID-19 vs. general population	Mean_lingual_thickness	Inverse variance weighted	28	0.0041	0.0021	0.0595	0.0000	0.0081
COVID-19 vs. general population	Mean_medialorbitofrontal_surface_area	MR Egger	28	4.1336	6.3692	0.5220	-8.3500	16.6172
COVID-19 vs. general population	Mean_medialorbitofrontal_surface_area	Weighted median	28	-3.0742	3.8317	0.4224	-10.5844	4.4360
COVID-19 vs. general population	Mean_medialorbitofrontal_surface_area	Inverse variance weighted	28	-2.6749	2.6170	0.3067	-7.8041	2.4544
COVID-19 vs. general population	Mean_medialorbitofrontal_thickness	MR Egger	28	0.0099	0.0066	0.1481	-0.0031	0.0229
COVID-19 vs. general population	Mean_medialorbitofrontal_thickness	Weighted median	28	0.0070	0.0038	0.0669	-0.0005	0.0145
COVID-19 vs. general population	Mean_medialorbitofrontal_thickness	Inverse variance weighted	28	0.0031	0.0028	0.2589	-0.0023	0.0085

continued:

COVID-19 phenotype	Brain structure	Method	nsnp	β	se	p value	β_low95%CI	β_up95%CI
COVID-19 vs. general population	Mean_middletemporal_surface_area	MR Egger	28	-27.2570	11.4211	0.0246	-49.6424	-4.8715
COVID-19 vs. general population	Mean_middletemporal_surface_area	Weighted median	28	0.8246	6.9230	0.9052	-12.7444	14.3937
COVID-19 vs. general population	Mean_middletemporal_surface_area	Inverse variance weighted	28	1.0185	5.2015	0.8448	-9.1765	11.2135
COVID-19 vs. general population	Mean_middletemporal_thickness	MR Egger	28	0.0027	0.0051	0.6069	-0.0074	0.0128
COVID-19 vs. general population	Mean_middletemporal_thickness	Weighted median	28	-0.0039	0.0031	0.2119	-0.0100	0.0022
COVID-19 vs. general population	Mean_middletemporal_thickness	Inverse variance weighted	28	-0.0013	0.0021	0.5365	-0.0054	0.0028
COVID-19 vs. general population	Mean_paracentral_surface_area	MR Egger	28	-6.9314	6.9258	0.3261	-20.5059	6.6431
COVID-19 vs. general population	Mean_paracentral_surface_area	Weighted median	28	-4.8447	4.2081	0.2496	-13.0926	3.4031
COVID-19 vs. general population	Mean_paracentral_surface_area	Inverse variance weighted	28	-5.4368	2.8471	0.0562	-11.0172	0.1436
COVID-19 vs. general population	Mean_paracentral_thickness	MR Egger	28	-0.0021	0.0052	0.6865	-0.0124	0.0081
COVID-19 vs. general population	Mean_paracentral_thickness	Weighted median	28	-0.0029	0.0031	0.3501	-0.0090	0.0032
COVID-19 vs. general population	Mean_paracentral_thickness	Inverse variance weighted	28	-0.0029	0.0021	0.1782	-0.0071	0.0013
COVID-19 vs. general population	Mean_parahippocampal_surface_area	MR Egger	28	-1.6431	4.1006	0.6919	-9.6802	6.3941
COVID-19 vs. general population	Mean_parahippocampal_surface_area	Weighted median	28	-1.2012	2.1599	0.5781	-5.4346	3.0322
COVID-19 vs. general population	Mean_parahippocampal_surface_area	Inverse variance weighted	28	-1.5647	1.6559	0.3447	-4.8103	1.6808
COVID-19 vs. general population	Mean_parahippocampal_thickness	MR Egger	28	-0.0065	0.0128	0.6163	-0.0315	0.0186
COVID-19 vs. general population	Mean_parahippocampal_thickness	Weighted median	28	-0.0056	0.0076	0.4667	-0.0205	0.0094
COVID-19 vs. general population	Mean_parahippocampal_thickness	Inverse variance weighted	28	-0.0029	0.0052	0.5815	-0.0131	0.0074
COVID-19 vs. general population	Mean_parsopercularis_surface_area	MR Egger	28	6.5833	10.1129	0.5208	-13.2379	26.4046
COVID-19 vs. general population	Mean_parsopercularis_surface_area	Weighted median	28	-5.1767	5.1026	0.3103	-15.1778	4.8244
COVID-19 vs. general population	Mean_parsopercularis_surface_area	Inverse variance weighted	28	-4.2506	4.1874	0.3101	-12.4580	3.9567
COVID-19 vs. general population	Mean_parsopercularis_thickness	MR Egger	28	-0.0011	0.0050	0.8250	-0.0109	0.0087
COVID-19 vs. general population	Mean_parsopercularis_thickness	Weighted median	28	-0.0019	0.0027	0.4841	-0.0072	0.0034
COVID-19 vs. general population	Mean_parsopercularis_thickness	Inverse variance weighted	28	-0.0007	0.0020	0.7397	-0.0046	0.0033
COVID-19 vs. general population	Mean_parsorbitalis_surface_area	MR Egger	28	4.5895	2.8805	0.1232	-1.0562	10.2353
COVID-19 vs. general population	Mean_parsorbitalis_surface_area	Weighted median	28	1.2013	1.7825	0.5004	-2.2924	4.6951
COVID-19 vs. general population	Mean_parsorbitalis_surface_area	Inverse variance weighted	28	0.1245	1.2219	0.9188	-2.2705	2.5195
COVID-19 vs. general population	Mean_parsorbitalis_thickness	MR Egger	28	-0.0018	0.0070	0.8002	-0.0156	0.0120
COVID-19 vs. general population	Mean_parsorbitalis_thickness	Weighted median	28	0.0022	0.0044	0.6220	-0.0064	0.0108
COVID-19 vs. general population	Mean_parsorbitalis_thickness	Inverse variance weighted	28	-0.0001	0.0029	0.9756	-0.0057	0.0055
COVID-19 vs. general population	Mean_parstriangularis_surface_area	MR Egger	28	-4.9142	7.5999	0.5236	-19.8099	9.9816
COVID-19 vs. general population	Mean_parstriangularis_surface_area	Weighted median	28	-4.1851	4.3736	0.3386	-12.7575	4.3872

continued:

COVID-19 phenotype	Brain structure	Method	nsnp	β	se	p value	β_low95%CI	β_up95%CI
COVID-19 vs. general population	Mean_parstriangularis_surface_area	Inverse variance weighted	28	-4.4146	3.1250	0.1577	-10.5396	1.7103
COVID-19 vs. general population	Mean_parstriangularis_thickness	MR Egger	28	0.0016	0.0048	0.7494	-0.0079	0.0110
COVID-19 vs. general population	Mean_parstriangularis_thickness	Weighted median	28	-0.0005	0.0029	0.8722	-0.0061	0.0052
COVID-19 vs. general population	Mean_parstriangularis_thickness	Inverse variance weighted	28	-0.0005	0.0020	0.7955	-0.0044	0.0033
COVID-19 vs. general population	Mean_pericalcarine_surface_area	MR Egger	28	-4.2870	9.3612	0.6508	-22.6350	14.0610
COVID-19 vs. general population	Mean_pericalcarine_surface_area	Weighted median	28	-1.7695	5.8226	0.7612	-13.1817	9.6428
COVID-19 vs. general population	Mean_pericalcarine_surface_area	Inverse variance weighted	28	1.0003	3.8499	0.7950	-6.5455	8.5461
COVID-19 vs. general population	Mean_pericalcarine_thickness	MR Egger	28	0.0027	0.0056	0.6331	-0.0083	0.0137
COVID-19 vs. general population	Mean_pericalcarine_thickness	Weighted median	28	0.0000	0.0033	0.9951	-0.0064	0.0064
COVID-19 vs. general population	Mean_pericalcarine_thickness	Inverse variance weighted	28	0.0006	0.0023	0.7868	-0.0038	0.0050
COVID-19 vs. general population	Mean_postcentral_surface_area	MR Egger	28	-5.5867	13.2669	0.6771	-31.5899	20.4165
COVID-19 vs. general population	Mean_postcentral_surface_area	Weighted median	28	0.2188	8.1331	0.9785	-15.7220	16.1596
COVID-19 vs. general population	Mean_postcentral_surface_area	Inverse variance weighted	28	4.1985	5.4548	0.4415	-6.4929	14.8899
COVID-19 vs. general population	Mean_postcentral_thickness	MR Egger	28	-0.0046	0.0052	0.3855	-0.0147	0.0056
COVID-19 vs. general population	Mean_postcentral_thickness	Weighted median	28	0.0009	0.0025	0.7220	-0.0041	0.0059
COVID-19 vs. general population	Mean_postcentral_thickness	Inverse variance weighted	28	0.0005	0.0021	0.8266	-0.0037	0.0046
COVID-19 vs. general population	Mean_posteriorcingulate_surface_area	MR Egger	28	-10.0341	5.3472	0.0719	-20.5146	0.4464
COVID-19 vs. general population	Mean_posteriorcingulate_surface_area	Weighted median	28	-1.5070	3.1273	0.6299	-7.6365	4.6225
COVID-19 vs. general population	Mean_posteriorcingulate_surface_area	Inverse variance weighted	28	-0.8269	2.1980	0.7067	-5.1350	3.4811
COVID-19 vs. general population	Mean_posteriorcingulate_thickness	MR Egger	28	0.0084	0.0057	0.1525	-0.0028	0.0197
COVID-19 vs. general population	Mean_posteriorcingulate_thickness	Weighted median	28	0.0016	0.0034	0.6468	-0.0051	0.0082
COVID-19 vs. general population	Mean_posteriorcingulate_thickness	Inverse variance weighted	28	-0.0012	0.0025	0.6228	-0.0060	0.0036
COVID-19 vs. general population	Mean_precentral_surface_area	MR Egger	28	9.1218	15.5967	0.5637	-21.4478	39.6913
COVID-19 vs. general population	Mean_precentral_surface_area	Weighted median	28	5.5206	9.3999	0.5570	-12.9033	23.9444
COVID-19 vs. general population	Mean_precentral_surface_area	Inverse variance weighted	28	1.0064	6.4126	0.8753	-11.5623	13.5750
COVID-19 vs. general population	Mean_precentral_thickness	MR Egger	28	0.0016	0.0046	0.7327	-0.0073	0.0105
COVID-19 vs. general population	Mean_precentral_thickness	Weighted median	28	-0.0004	0.0026	0.8842	-0.0055	0.0048
COVID-19 vs. general population	Mean_precentral_thickness	Inverse variance weighted	28	-0.0012	0.0019	0.5075	-0.0049	0.0024
COVID-19 vs. general population	Mean_precuneus_surface_area	MR Egger	28	0.1546	13.3871	0.9909	-26.0842	26.3934
COVID-19 vs. general population	Mean_precuneus_surface_area	Weighted median	28	9.7449	8.2370	0.2368	-6.3996	25.8894
COVID-19 vs. general population	Mean_precuneus_surface_area	Inverse variance weighted	28	1.4301	5.4772	0.7940	-9.3052	12.1653
COVID-19 vs. general population	Mean_precuneus_thickness	MR Egger	28	-0.0003	0.0046	0.9506	-0.0092	0.0086

continued:

COVID-19 phenotype	Brain structure	Method	nsnp	β	se	p value	β_low95%CI	β_up95%CI
COVID-19 vs. general population	Mean_precuneus_thickness	Weighted median	28	0.0013	0.0025	0.6140	-0.0036	0.0062
COVID-19 vs. general population	Mean_precuneus_thickness	Inverse variance weighted	28	0.0015	0.0018	0.4242	-0.0021	0.0050
COVID-19 vs. general population	Mean_rostralanteriorcingulate_surface_area	MR Egger	28	-2.0035	4.8579	0.6834	-11.5251	7.5180
COVID-19 vs. general population	Mean_rostralanteriorcingulate_surface_area	Weighted median	28	-4.6924	2.8458	0.0992	-10.2703	0.8854
COVID-19 vs. general population	Mean_rostralanteriorcingulate_surface_area	Inverse variance weighted	28	-2.7231	1.9589	0.1645	-6.5624	1.1163
COVID-19 vs. general population	Mean_rostralanteriorcingulate_thickness	MR Egger	28	0.0061	0.0085	0.4806	-0.0105	0.0227
COVID-19 vs. general population	Mean_rostralanteriorcingulate_thickness	Weighted median	28	0.0040	0.0048	0.4028	-0.0054	0.0134
COVID-19 vs. general population	Mean_rostralanteriorcingulate_thickness	Inverse variance weighted	28	0.0013	0.0035	0.7133	-0.0055	0.0081
COVID-19 vs. general population	Mean_rostralmiddlefrontal_surface_area	MR Egger	28	-30.6049	22.1207	0.1783	-73.9615	12.7517
COVID-19 vs. general population	Mean_rostralmiddlefrontal_surface_area	Weighted median	28	-1.3303	11.8433	0.9106	-24.5432	21.8825
COVID-19 vs. general population	Mean_rostralmiddlefrontal_surface_area	Inverse variance weighted	28	-9.3374	9.1102	0.3054	-27.1934	8.5185
COVID-19 vs. general population	Mean_rostralmiddlefrontal_thickness	MR Egger	28	0.0012	0.0040	0.7584	-0.0065	0.0090
COVID-19 vs. general population	Mean_rostralmiddlefrontal_thickness	Weighted median	28	-0.0015	0.0024	0.5455	-0.0062	0.0033
COVID-19 vs. general population	Mean_rostralmiddlefrontal_thickness	Inverse variance weighted	28	0.0003	0.0016	0.8533	-0.0029	0.0035
COVID-19 vs. general population	Mean_superiorfrontal_surface_area	MR Egger	28	0.4083	22.1700	0.9854	-43.0450	43.8616
COVID-19 vs. general population	Mean_superiorfrontal_surface_area	Weighted median	28	-1.6463	12.1393	0.8921	-25.4394	22.1468
COVID-19 vs. general population	Mean_superiorfrontal_surface_area	Inverse variance weighted	28	-12.9865	9.0156	0.1497	-30.6570	4.6840
COVID-19 vs. general population	Mean_superiorfrontal_thickness	MR Egger	28	0.0029	0.0048	0.5538	-0.0065	0.0123
COVID-19 vs. general population	Mean_superiorfrontal_thickness	Weighted median	28	-0.0020	0.0026	0.4505	-0.0071	0.0032
COVID-19 vs. general population	Mean_superiorfrontal_thickness	Inverse variance weighted	28	-0.0041	0.0020	0.0389	-0.0081	-0.0002
COVID-19 vs. general population	Mean_superiorparietal_surface_area	MR Egger	28	10.4453	19.0528	0.5882	-26.8981	47.7888
COVID-19 vs. general population	Mean_superiorparietal_surface_area	Weighted median	28	-7.6812	10.8980	0.4809	-29.0413	13.6788
COVID-19 vs. general population	Mean_superiorparietal_surface_area	Inverse variance weighted	28	5.5976	7.8331	0.4749	-9.7554	20.9505
COVID-19 vs. general population	Mean_superiorparietal_thickness	MR Egger	28	-0.0048	0.0038	0.2142	-0.0123	0.0026
COVID-19 vs. general population	Mean_superiorparietal_thickness	Weighted median	28	-0.0001	0.0022	0.9551	-0.0045	0.0042
COVID-19 vs. general population	Mean_superiorparietal_thickness	Inverse variance weighted	28	0.0016	0.0015	0.2941	-0.0014	0.0046
COVID-19 vs. general population	Mean_superiortemporal_surface_area	MR Egger	28	16.7238	12.2026	0.1822	-7.1933	40.6409
COVID-19 vs. general population	Mean_superiortemporal_surface_area	Weighted median	28	-2.6837	6.9452	0.6992	-16.2963	10.9289
COVID-19 vs. general population	Mean_superiortemporal_surface_area	Inverse variance weighted	28	0.4942	5.1199	0.9231	-9.5408	10.5293
COVID-19 vs. general population	Mean_superiortemporal_thickness	MR Egger	28	0.0018	0.0054	0.7463	-0.0088	0.0123
COVID-19 vs. general population	Mean_superiortemporal_thickness	Weighted median	28	0.0030	0.0030	0.3129	-0.0028	0.0088
COVID-19 vs. general population	Mean_superiortemporal_thickness	Inverse variance weighted	28	0.0014	0.0022	0.5252	-0.0029	0.0057

continued:

COVID-19 phenotype	Brain structure	Method	nsnp	β	se	p value	β_low95%CI	β_up95%CI
COVID-19 vs. general population	Mean_supramarginal_surface_area	MR Egger	28	1.8761	15.2783	0.9032	-28.0694	31.8216
COVID-19 vs. general population	Mean_supramarginal_surface_area	Weighted median	28	7.8249	8.5784	0.3617	-8.9886	24.6385
COVID-19 vs. general population	Mean_supramarginal_surface_area	Inverse variance weighted	28	9.5464	6.2796	0.1285	-2.7617	21.8544
COVID-19 vs. general population	Mean_supramarginal_thickness	MR Egger	28	0.0016	0.0036	0.6582	-0.0054	0.0086
COVID-19 vs. general population	Mean_supramarginal_thickness	Weighted median	28	-0.0002	0.0022	0.9343	-0.0045	0.0041
COVID-19 vs. general population	Mean_supramarginal_thickness	Inverse variance weighted	28	-0.0004	0.0015	0.7977	-0.0033	0.0025
COVID-19 vs. general population	Mean_temporalpole_surface_area	MR Egger	28	2.6769	2.3781	0.2706	-1.9841	7.3380
COVID-19 vs. general population	Mean_temporalpole_surface_area	Weighted median	28	2.1164	1.4197	0.1360	-0.6662	4.8990
COVID-19 vs. general population	Mean_temporalpole_surface_area	Inverse variance weighted	28	1.1837	0.9681	0.2214	-0.7137	3.0812
COVID-19 vs. general population	Mean_temporalpole_thickness	MR Egger	28	0.0062	0.0136	0.6498	-0.0204	0.0329
COVID-19 vs. general population	Mean_temporalpole_thickness	Weighted median	28	0.0000	0.0076	0.9959	-0.0149	0.0148
COVID-19 vs. general population	Mean_temporalpole_thickness	Inverse variance weighted	28	0.0018	0.0055	0.7470	-0.0090	0.0125
COVID-19 vs. general population	Mean_transversetemporal_surface_area	MR Egger	28	-0.7524	2.3958	0.7560	-5.4482	3.9434
COVID-19 vs. general population	Mean_transversetemporal_surface_area	Weighted median	28	-0.5244	1.3714	0.7022	-3.2124	2.1636
COVID-19 vs. general population	Mean_transversetemporal_surface_area	Inverse variance weighted	28	-0.7471	0.9754	0.4437	-2.6589	1.1646
COVID-19 vs. general population	Mean_transversetemporal_thickness	MR Egger	28	0.0094	0.0091	0.3103	-0.0084	0.0273
COVID-19 vs. general population	Mean_transversetemporal_thickness	Weighted median	28	0.0005	0.0050	0.9131	-0.0093	0.0104
COVID-19 vs. general population	Mean_transversetemporal_thickness	Inverse variance weighted	28	-0.0046	0.0039	0.2409	-0.0122	0.0031
COVID-19 vs. general population	Mean_Amygdala_volume	MR Egger	31	4.8553	15.4024	0.7548	-25.3334	35.0440
COVID-19 vs. general population	Mean_Amygdala_volume	Weighted median	31	0.5469	8.9599	0.9513	-17.0146	18.1084
COVID-19 vs. general population	Mean_Amygdala_volume	Inverse variance weighted	31	-1.6977	6.1842	0.7837	-13.8187	10.4233
COVID-19 vs. general population	Mean_Caudate_volume	MR Egger	30	-1.3196	29.2654	0.9644	-58.6798	56.0405
COVID-19 vs. general population	Mean_Caudate_volume	Weighted median	30	12.6569	17.4441	0.4681	-21.5334	46.8473
COVID-19 vs. general population	Mean_Caudate_volume	Inverse variance weighted	30	-0.5613	11.9429	0.9625	-23.9694	22.8467
COVID-19 vs. general population	Mean_Hippocampus_volume	MR Egger	31	18.4409	30.6635	0.5522	-41.6596	78.5414
COVID-19 vs. general population	Mean_Hippocampus_volume	Weighted median	31	3.3197	17.0652	0.8458	-30.1280	36.7674
COVID-19 vs. general population	Mean_Hippocampus_volume	Inverse variance weighted	31	-6.9811	12.4478	0.5749	-31.3788	17.4167
COVID-19 vs. general population	Mean_Intracranial_volume	MR Egger	30	1065.2237	11512.9949	0.9269	-21500.2464	23630.6938
COVID-19 vs. general population	Mean_Intracranial_volume	Weighted median	30	-6988.7103	6915.4412	0.3122	-20542.9751	6565.5544
COVID-19 vs. general population	Mean_Intracranial_volume	Inverse variance weighted	30	-7016.4873	4714.1142	0.1366	-16256.1511	2223.1765
COVID-19 vs. general population	Mean_Nucleus_accumbens_volume	MR Egger	31	5.3589	6.5558	0.4203	-7.4905	18.2083
COVID-19 vs. general population	Mean_Nucleus_accumbens_volume	Weighted median	31	1.4594	3.6794	0.6916	-5.7523	8.6710

continued:

COVID-19 phenotype	Brain structure	Method	nsnp	β	se	p value	β_low95%CI	β_up95%CI
COVID-19 vs. general population	Mean_Nucleus_accumbens_volume	Inverse variance weighted	31	2.9304	2.6679	0.2720	-2.2987	8.1596
COVID-19 vs. general population	Mean_Pallidum_volume	MR Egger	30	3.4079	11.5220	0.7696	-19.1752	25.9911
COVID-19 vs. general population	Mean_Pallidum_volume	Weighted median	30	-0.0266	6.8688	0.9969	-13.4895	13.4363
COVID-19 vs. general population	Mean_Pallidum_volume	Inverse variance weighted	30	1.4082	4.6914	0.7640	-7.7870	10.6034
COVID-19 vs. general population	Mean_Putamen_volume	MR Egger	31	36.3964	37.4651	0.3393	-37.0351	109.8280
COVID-19 vs. general population	Mean_Putamen_volume	Weighted median	31	31.3324	23.1738	0.1764	-14.0882	76.7531
COVID-19 vs. general population	Mean_Putamen_volume	Inverse variance weighted	31	15.9520	15.1276	0.2917	-13.6981	45.6021
COVID-19 vs. general population	Mean_Thalamus_volume	MR Egger	30	17.3397	37.4528	0.6470	-56.0677	90.7471
COVID-19 vs. general population	Mean_Thalamus_volume	Weighted median	30	23.0262	22.9781	0.3163	-22.0109	68.0633
COVID-19 vs. general population	Mean_Thalamus_volume	Inverse variance weighted	30	20.3817	15.2860	0.1824	-9.5789	50.3423

Abbreviations: COVID-19, coronavirus disease 2019; CI, confidence interval; nSNPs, number of single-nucleotide polymorphisms; se, standard error

Table S3. MR analysis of the causal relationship between hospitalized COVID-19 and brain structure

COVID-19 phenotype	Brain structure	Method	nsnp	β	se	p value	β_low95%CI	β_up95%CI
Hospitalized COVID-19 vs. general population	Mean_bankssts_surface_area	MR Egger	24	-0.2502	6.0635	0.9675	-12.1346	11.6343
Hospitalized COVID-19 vs. general population	Mean_bankssts_surface_area	Weighted median	24	1.7908	3.2217	0.5783	-4.5237	8.1053
Hospitalized COVID-19 vs. general population	Mean_bankssts_surface_area	Inverse variance weighted	24	0.4807	2.2939	0.8340	-4.0153	4.9767
Hospitalized COVID-19 vs. general population	Mean_bankssts_thickness	MR Egger	24	0.0085	0.0073	0.2574	-0.0059	0.0229
Hospitalized COVID-19 vs. general population	Mean_bankssts_thickness	Weighted median	24	0.0022	0.0038	0.5582	-0.0053	0.0097
Hospitalized COVID-19 vs. general population	Mean_bankssts_thickness	Inverse variance weighted	24	0.0031	0.0027	0.2595	-0.0023	0.0085
Hospitalized COVID-19 vs. general population	Mean_caudalanteriorcingulate_surface_area	MR Egger	24	-2.3458	5.5313	0.6756	-13.1871	8.4955
Hospitalized COVID-19 vs. general population	Mean_caudalanteriorcingulate_surface_area	Weighted median	24	-2.7573	2.9105	0.3434	-8.4618	2.9472
Hospitalized COVID-19 vs. general population	Mean_caudalanteriorcingulate_surface_area	Inverse variance weighted	24	-2.1591	2.0481	0.2918	-6.1734	1.8552
Hospitalized COVID-19 vs. general population	Mean_caudalanteriorcingulate_thickness	MR Egger	24	0.0038	0.0153	0.8084	-0.0263	0.0338
Hospitalized COVID-19 vs. general population	Mean_caudalanteriorcingulate_thickness	Weighted median	24	-0.0007	0.0065	0.9105	-0.0135	0.0121
Hospitalized COVID-19 vs. general population	Mean_caudalanteriorcingulate_thickness	Inverse variance weighted	24	0.0010	0.0057	0.8663	-0.0102	0.0121
Hospitalized COVID-19 vs. general population	Mean_caudalmiddlefrontal_surface_area	MR Egger	24	1.9048	16.3637	0.9084	-30.1681	33.9778
Hospitalized COVID-19 vs. general population	Mean_caudalmiddlefrontal_surface_area	Weighted median	24	2.7549	7.5544	0.7154	-12.0517	17.5615
Hospitalized COVID-19 vs. general population	Mean_caudalmiddlefrontal_surface_area	Inverse variance weighted	24	4.0974	6.0611	0.4990	-7.7824	15.9771
Hospitalized COVID-19 vs. general population	Mean_caudalmiddlefrontal_thickness	MR Egger	24	-0.0026	0.0062	0.6780	-0.0148	0.0096
Hospitalized COVID-19 vs. general population	Mean_caudalmiddlefrontal_thickness	Weighted median	24	-0.0010	0.0030	0.7346	-0.0068	0.0048
Hospitalized COVID-19 vs. general population	Mean_caudalmiddlefrontal_thickness	Inverse variance weighted	24	-0.0022	0.0023	0.3301	-0.0068	0.0023
Hospitalized COVID-19 vs. general population	Mean_cuneus_surface_area	MR Egger	24	4.9666	8.2009	0.5510	-11.1071	21.0403
Hospitalized COVID-19 vs. general population	Mean_cuneus_surface_area	Weighted median	24	-0.9961	4.2579	0.8150	-9.3415	7.3494
Hospitalized COVID-19 vs. general population	Mean_cuneus_surface_area	Inverse variance weighted	24	-1.9845	3.1055	0.5228	-8.0713	4.1023
Hospitalized COVID-19 vs. general population	Mean_cuneus_thickness	MR Egger	24	0.0038	0.0060	0.5328	-0.0079	0.0155
Hospitalized COVID-19 vs. general population	Mean_cuneus_thickness	Weighted median	24	0.0011	0.0032	0.7429	-0.0052	0.0073
Hospitalized COVID-19 vs. general population	Mean_cuneus_thickness	Inverse variance weighted	24	-0.0001	0.0023	0.9641	-0.0045	0.0043
Hospitalized COVID-19 vs. general population	Mean_entorhinal_surface_area	MR Egger	24	-3.2746	3.5577	0.3673	-10.2478	3.6985
Hospitalized COVID-19 vs. general population	Mean_entorhinal_surface_area	Weighted median	24	0.6585	1.8278	0.7187	-2.9240	4.2409
Hospitalized COVID-19 vs. general population	Mean_entorhinal_surface_area	Inverse variance weighted	24	1.5484	1.3781	0.2612	-1.1528	4.2495
Hospitalized COVID-19 vs. general population	Mean_entorhinal_thickness	MR Egger	24	0.0021	0.0171	0.9052	-0.0315	0.0356
Hospitalized COVID-19 vs. general population	Mean_entorhinal_thickness	Weighted median	24	0.0010	0.0090	0.9148	-0.0167	0.0186
Hospitalized COVID-19 vs. general population	Mean_entorhinal_thickness	Inverse variance weighted	24	-0.0003	0.0065	0.9627	-0.0130	0.0124

continued:

COVID-19 phenotype	Brain structure	Method	nsnp	β	se	p value	β_low95%CI	β_up95%CI
Hospitalized COVID-19 vs. general population	Mean_frontalpole_surface_area	MR Egger	24	-2.1365	1.6118	0.1986	-5.2957	1.0226
Hospitalized COVID-19 vs. general population	Mean_frontalpole_surface_area	Weighted median	24	0.5060	0.8593	0.5560	-1.1782	2.1902
Hospitalized COVID-19 vs. general population	Mean_frontalpole_surface_area	Inverse variance weighted	24	-0.1897	0.6097	0.7557	-1.3847	1.0052
Hospitalized COVID-19 vs. general population	Mean_frontalpole_thickness	MR Egger	24	0.0054	0.0118	0.6498	-0.0177	0.0286
Hospitalized COVID-19 vs. general population	Mean_frontalpole_thickness	Weighted median	24	0.0040	0.0062	0.5122	-0.0080	0.0161
Hospitalized COVID-19 vs. general population	Mean_frontalpole_thickness	Inverse variance weighted	24	0.0075	0.0045	0.0917	-0.0012	0.0163
Hospitalized COVID-19 vs. general population	Mean_full_surface_area	Inverse variance weighted	1	-3230.7204	1185.0766	0.0064	-5553.4705	-907.9702
Hospitalized COVID-19 vs. general population	Mean_full_thickness	MR Egger	4	-0.0146	0.0260	0.6306	-0.0656	0.0364
Hospitalized COVID-19 vs. general population	Mean_full_thickness	Weighted median	4	-0.0075	0.0061	0.2138	-0.0194	0.0043
Hospitalized COVID-19 vs. general population	Mean_full_thickness	Inverse variance weighted	4	-0.0078	0.0051	0.1229	-0.0178	0.0021
Hospitalized COVID-19 vs. general population	Mean_fusiform_surface_area	MR Egger	24	-4.1116	13.7397	0.7676	-31.0413	22.8182
Hospitalized COVID-19 vs. general population	Mean_fusiform_surface_area	Weighted median	24	-6.5721	7.3902	0.3738	-21.0568	7.9127
Hospitalized COVID-19 vs. general population	Mean_fusiform_surface_area	Inverse variance weighted	24	-6.8004	5.1294	0.1849	-16.8540	3.2533
Hospitalized COVID-19 vs. general population	Mean_fusiform_thickness	MR Egger	24	0.0019	0.0056	0.7424	-0.0091	0.0129
Hospitalized COVID-19 vs. general population	Mean_fusiform_thickness	Weighted median	24	-0.0040	0.0030	0.1739	-0.0099	0.0018
Hospitalized COVID-19 vs. general population	Mean_fusiform_thickness	Inverse variance weighted	24	-0.0010	0.0021	0.6320	-0.0052	0.0032
Hospitalized COVID-19 vs. general population	Mean_inferiorparietal_surface_area	MR Egger	24	-11.2333	30.7952	0.7188	-71.5920	49.1253
Hospitalized COVID-19 vs. general population	Mean_inferiorparietal_surface_area	Weighted median	24	-8.4867	13.6119	0.5330	-35.1660	18.1926
Hospitalized COVID-19 vs. general population	Mean_inferiorparietal_surface_area	Inverse variance weighted	24	5.5924	11.4912	0.6265	-16.9304	28.1151
Hospitalized COVID-19 vs. general population	Mean_inferiorparietal_thickness	MR Egger	24	0.0039	0.0041	0.3606	-0.0042	0.0120
Hospitalized COVID-19 vs. general population	Mean_inferiorparietal_thickness	Weighted median	24	0.0016	0.0022	0.4536	-0.0026	0.0059
Hospitalized COVID-19 vs. general population	Mean_inferiorparietal_thickness	Inverse variance weighted	24	0.0010	0.0016	0.5131	-0.0020	0.0041
Hospitalized COVID-19 vs. general population	Mean_inferiortemporal_surface_area	MR Egger	24	5.1894	17.7608	0.7729	-29.6217	40.0005
Hospitalized COVID-19 vs. general population	Mean_inferiortemporal_surface_area	Weighted median	24	-2.1233	8.2901	0.7979	-18.3718	14.1253
Hospitalized COVID-19 vs. general population	Mean_inferiortemporal_surface_area	Inverse variance weighted	24	-2.8455	6.6122	0.6670	-15.8054	10.1145
Hospitalized COVID-19 vs. general population	Mean_inferiortemporal_thickness	MR Egger	24	0.0007	0.0063	0.9175	-0.0117	0.0130
Hospitalized COVID-19 vs. general population	Mean_inferiortemporal_thickness	Weighted median	24	0.0005	0.0032	0.8776	-0.0058	0.0068
Hospitalized COVID-19 vs. general population	Mean_inferiortemporal_thickness	Inverse variance weighted	24	-0.0009	0.0024	0.7036	-0.0056	0.0038
Hospitalized COVID-19 vs. general population	Mean_insula_surface_area	MR Egger	24	3.6007	9.2085	0.6995	-14.4480	21.6494

continued:

COVID-19 phenotype	Brain structure	Method	nsnp	β	se	p value	β_low95%CI	β_up95%CI
Hospitalized COVID-19 vs. general population	Mean_insula_surface_area	Weighted median	24	1.4483	4.7410	0.7600	-7.8441	10.7407
Hospitalized COVID-19 vs. general population	Mean_insula_surface_area	Inverse variance weighted	24	0.9518	3.4829	0.7846	-5.8747	7.7782
Hospitalized COVID-19 vs. general population	Mean_insula_thickness	MR Egger	24	-0.0163	0.0069	0.0271	-0.0297	-0.0028
Hospitalized COVID-19 vs. general population	Mean_insula_thickness	Weighted median	24	-0.0068	0.0036	0.0585	-0.0139	0.0002
Hospitalized COVID-19 vs. general population	Mean_insula_thickness	Inverse variance weighted	24	-0.0040	0.0027	0.1466	-0.0094	0.0014
Hospitalized COVID-19 vs. general population	Mean_isthmuscingulate_surface_area	MR Egger	24	-0.8233	6.6453	0.9025	-13.8481	12.2014
Hospitalized COVID-19 vs. general population	Mean_isthmuscingulate_surface_area	Weighted median	24	2.7840	3.4239	0.4162	-3.9270	9.4949
Hospitalized COVID-19 vs. general population	Mean_isthmuscingulate_surface_area	Inverse variance weighted	24	1.7303	2.4686	0.4834	-3.1083	6.5688
Hospitalized COVID-19 vs. general population	Mean_isthmuscingulate_thickness	MR Egger	24	0.0089	0.0095	0.3575	-0.0097	0.0275
Hospitalized COVID-19 vs. general population	Mean_isthmuscingulate_thickness	Weighted median	24	0.0029	0.0051	0.5745	-0.0071	0.0128
Hospitalized COVID-19 vs. general population	Mean_isthmuscingulate_thickness	Inverse variance weighted	24	0.0026	0.0036	0.4626	-0.0044	0.0096
Hospitalized COVID-19 vs. general population	Mean_lateraloccipital_surface_area	MR Egger	24	27.5187	23.5308	0.2547	-18.6018	73.6392
Hospitalized COVID-19 vs. general population	Mean_lateraloccipital_surface_area	Weighted median	24	8.9897	11.4013	0.4304	-13.3569	31.3362
Hospitalized COVID-19 vs. general population	Mean_lateraloccipital_surface_area	Inverse variance weighted	24	4.2891	8.9387	0.6313	-13.2307	21.8088
Hospitalized COVID-19 vs. general population	Mean_lateraloccipital_thickness	MR Egger	24	0.0052	0.0050	0.3025	-0.0045	0.0149
Hospitalized COVID-19 vs. general population	Mean_lateraloccipital_thickness	Weighted median	24	0.0028	0.0026	0.2824	-0.0023	0.0079
Hospitalized COVID-19 vs. general population	Mean_lateraloccipital_thickness	Inverse variance weighted	24	0.0036	0.0019	0.0540	-0.0001	0.0073
Hospitalized COVID-19 vs. general population	Mean_lateralorbitofrontal_surface_area	MR Egger	24	-4.0856	10.2425	0.6938	-24.1609	15.9897
Hospitalized COVID-19 vs. general population	Mean_lateralorbitofrontal_surface_area	Weighted median	24	0.0410	5.4021	0.9939	-10.5471	10.6290
Hospitalized COVID-19 vs. general population	Mean_lateralorbitofrontal_surface_area	Inverse variance weighted	24	-0.3909	3.8078	0.9182	-7.8541	7.0724
Hospitalized COVID-19 vs. general population	Mean_lateralorbitofrontal_thickness	MR Egger	26	-0.0033	0.0059	0.5838	-0.0147	0.0082
Hospitalized COVID-19 vs. general population	Mean_lateralorbitofrontal_thickness	Weighted median	26	-0.0034	0.0032	0.2949	-0.0096	0.0029
Hospitalized COVID-19 vs. general population	Mean_lateralorbitofrontal_thickness	Inverse variance weighted	26	-0.0049	0.0023	0.0328	-0.0093	-0.0004
Hospitalized COVID-19 vs. general population	Mean_lingual_surface_area	MR Egger	24	14.3404	16.4035	0.3914	-17.8105	46.4912
Hospitalized COVID-19 vs. general population	Mean_lingual_surface_area	Weighted median	24	4.1179	8.5335	0.6294	-12.6078	20.8437
Hospitalized COVID-19 vs. general population	Mean_lingual_surface_area	Inverse variance weighted	24	5.9115	6.2117	0.3413	-6.2634	18.0865
Hospitalized COVID-19 vs. general population	Mean_lingual_thickness	MR Egger	24	0.0014	0.0062	0.8242	-0.0108	0.0136
Hospitalized COVID-19 vs. general population	Mean_lingual_thickness	Weighted median	24	0.0006	0.0029	0.8426	-0.0051	0.0062
Hospitalized COVID-19 vs. general population	Mean_lingual_thickness	Inverse variance weighted	24	0.0028	0.0023	0.2197	-0.0017	0.0073

continued:

COVID-19 phenotype	Brain structure	Method	nsnp	β	se	p value	β_low95%CI	β_up95%CI
Hospitalized COVID-19 vs. general population	Mean_medialorbitofrontal_surface_area	MR Egger	24	0.5231	7.4463	0.9446	-14.0716	15.1178
Hospitalized COVID-19 vs. general population	Mean_medialorbitofrontal_surface_area	Weighted median	24	-4.1229	3.9055	0.2911	-11.7777	3.5320
Hospitalized COVID-19 vs. general population	Mean_medialorbitofrontal_surface_area	Inverse variance weighted	24	-2.2476	2.7950	0.4213	-7.7258	3.2307
Hospitalized COVID-19 vs. general population	Mean_medialorbitofrontal_thickness	MR Egger	24	-0.0117	0.0073	0.1226	-0.0260	0.0026
Hospitalized COVID-19 vs. general population	Mean_medialorbitofrontal_thickness	Weighted median	24	-0.0054	0.0038	0.1535	-0.0129	0.0020
Hospitalized COVID-19 vs. general population	Mean_medialorbitofrontal_thickness	Inverse variance weighted	24	-0.0021	0.0028	0.4413	-0.0075	0.0033
Hospitalized COVID-19 vs. general population	Mean_middletemporal_surface_area	MR Egger	26	-4.0122	12.6176	0.7532	-28.7428	20.7184
Hospitalized COVID-19 vs. general population	Mean_middletemporal_surface_area	Weighted median	26	-15.0362	6.9142	0.0297	-28.5881	-1.4844
Hospitalized COVID-19 vs. general population	Mean_middletemporal_surface_area	Inverse variance weighted	26	-10.8855	4.9088	0.0266	-20.5067	-1.2642
Hospitalized COVID-19 vs. general population	Mean_middletemporal_thickness	MR Egger	24	-0.0015	0.0059	0.8012	-0.0130	0.0100
Hospitalized COVID-19 vs. general population	Mean_middletemporal_thickness	Weighted median	24	0.0004	0.0031	0.8978	-0.0057	0.0065
Hospitalized COVID-19 vs. general population	Mean_middletemporal_thickness	Inverse variance weighted	24	0.0007	0.0022	0.7641	-0.0037	0.0051
Hospitalized COVID-19 vs. general population	Mean_paracentral_surface_area	MR Egger	24	-9.7585	8.0420	0.2378	-25.5209	6.0038
Hospitalized COVID-19 vs. general population	Mean_paracentral_surface_area	Weighted median	24	1.3896	4.3966	0.7520	-7.2277	10.0068
Hospitalized COVID-19 vs. general population	Mean_paracentral_surface_area	Inverse variance weighted	24	1.7151	3.0456	0.5733	-4.2543	7.6845
Hospitalized COVID-19 vs. general population	Mean_paracentral_thickness	MR Egger	24	0.0091	0.0060	0.1451	-0.0027	0.0210
Hospitalized COVID-19 vs. general population	Mean_paracentral_thickness	Weighted median	24	0.0004	0.0032	0.9060	-0.0060	0.0067
Hospitalized COVID-19 vs. general population	Mean_paracentral_thickness	Inverse variance weighted	24	-0.0011	0.0023	0.6416	-0.0056	0.0034
Hospitalized COVID-19 vs. general population	Mean_parahippocampal_surface_area	MR Egger	24	-0.9094	4.7171	0.8489	-10.1550	8.3363
Hospitalized COVID-19 vs. general population	Mean_parahippocampal_surface_area	Weighted median	24	-0.7260	2.3118	0.7535	-5.2571	3.8052
Hospitalized COVID-19 vs. general population	Mean_parahippocampal_surface_area	Inverse variance weighted	24	-1.4330	1.7345	0.4087	-4.8327	1.9666
Hospitalized COVID-19 vs. general population	Mean_parahippocampal_thickness	MR Egger	24	0.0027	0.0151	0.8618	-0.0269	0.0322
Hospitalized COVID-19 vs. general population	Mean_parahippocampal_thickness	Weighted median	24	0.0091	0.0079	0.2519	-0.0064	0.0246
Hospitalized COVID-19 vs. general population	Mean_parahippocampal_thickness	Inverse variance weighted	24	0.0038	0.0056	0.4981	-0.0072	0.0148
Hospitalized COVID-19 vs. general population	Mean_parsopercularis_surface_area	MR Egger	24	-18.7048	9.5227	0.0623	-37.3693	-0.0402
Hospitalized COVID-19 vs. general population	Mean_parsopercularis_surface_area	Weighted median	24	0.2764	5.0736	0.9566	-9.6678	10.2205
Hospitalized COVID-19 vs. general population	Mean_parsopercularis_surface_area	Inverse variance weighted	24	0.7515	3.7989	0.8432	-6.6944	8.1973
Hospitalized COVID-19 vs. general population	Mean_parsopercularis_thickness	MR Egger	24	-0.0037	0.0062	0.5505	-0.0158	0.0083
Hospitalized COVID-19 vs. general population	Mean_parsopercularis_thickness	Weighted median	24	-0.0006	0.0029	0.8252	-0.0062	0.0050

continued:

COVID-19 phenotype	Brain structure	Method	nsnp	β	se	p value	β_low95%CI	β_up95%CI
Hospitalized COVID-19 vs. general population	Mean_parsopercularis_thickness	Inverse variance weighted	24	-0.0008	0.0023	0.7187	-0.0053	0.0036
Hospitalized COVID-19 vs. general population	Mean_parsorbitalis_surface_area	MR Egger	24	1.1483	3.3410	0.7343	-5.4001	7.6968
Hospitalized COVID-19 vs. general population	Mean_parsorbitalis_surface_area	Weighted median	24	1.0052	1.7730	0.5708	-2.4700	4.4803
Hospitalized COVID-19 vs. general population	Mean_parsorbitalis_surface_area	Inverse variance weighted	24	0.9630	1.2656	0.4467	-1.5175	3.4434
Hospitalized COVID-19 vs. general population	Mean_parsorbitalis_thickness	MR Egger	24	0.0035	0.0088	0.6972	-0.0138	0.0207
Hospitalized COVID-19 vs. general population	Mean_parsorbitalis_thickness	Weighted median	24	-0.0083	0.0045	0.0688	-0.0171	0.0006
Hospitalized COVID-19 vs. general population	Mean_parsorbitalis_thickness	Inverse variance weighted	24	-0.0071	0.0034	0.0342	-0.0137	-0.0005
Hospitalized COVID-19 vs. general population	Mean_parstriangularis_surface_area	MR Egger	24	-15.1946	8.8271	0.0992	-32.4958	2.1066
Hospitalized COVID-19 vs. general population	Mean_parstriangularis_surface_area	Weighted median	24	1.5366	4.7780	0.7477	-7.8282	10.9015
Hospitalized COVID-19 vs. general population	Mean_parstriangularis_surface_area	Inverse variance weighted	24	-0.2710	3.3411	0.9354	-6.8195	6.2775
Hospitalized COVID-19 vs. general population	Mean_parstriangularis_thickness	MR Egger	24	-0.0158	0.0057	0.0108	-0.0270	-0.0047
Hospitalized COVID-19 vs. general population	Mean_parstriangularis_thickness	Weighted median	24	-0.0021	0.0030	0.4875	-0.0079	0.0038
Hospitalized COVID-19 vs. general population	Mean_parstriangularis_thickness	Inverse variance weighted	24	-0.0021	0.0024	0.3769	-0.0068	0.0026
Hospitalized COVID-19 vs. general population	Mean_pericalcarine_surface_area	MR Egger	24	3.2623	10.8790	0.7671	-18.0606	24.5851
Hospitalized COVID-19 vs. general population	Mean_pericalcarine_surface_area	Weighted median	24	-0.2987	5.6536	0.9579	-11.3797	10.7824
Hospitalized COVID-19 vs. general population	Mean_pericalcarine_surface_area	Inverse variance weighted	24	-2.3592	4.1160	0.5665	-10.4266	5.7081
Hospitalized COVID-19 vs. general population	Mean_pericalcarine_thickness	MR Egger	24	0.0061	0.0058	0.3094	-0.0054	0.0175
Hospitalized COVID-19 vs. general population	Mean_pericalcarine_thickness	Weighted median	24	0.0015	0.0031	0.6327	-0.0046	0.0075
Hospitalized COVID-19 vs. general population	Mean_pericalcarine_thickness	Inverse variance weighted	24	0.0011	0.0022	0.6046	-0.0032	0.0054
Hospitalized COVID-19 vs. general population	Mean_postcentral_surface_area	MR Egger	24	-13.2893	15.4106	0.3978	-43.4941	16.9154
Hospitalized COVID-19 vs. general population	Mean_postcentral_surface_area	Weighted median	24	-2.6700	7.5616	0.7240	-17.4908	12.1508
Hospitalized COVID-19 vs. general population	Mean_postcentral_surface_area	Inverse variance weighted	24	-2.5810	5.8394	0.6585	-14.0262	8.8642
Hospitalized COVID-19 vs. general population	Mean_postcentral_thickness	MR Egger	24	0.0092	0.0048	0.0701	-0.0003	0.0187
Hospitalized COVID-19 vs. general population	Mean_postcentral_thickness	Weighted median	24	0.0029	0.0026	0.2600	-0.0021	0.0079
Hospitalized COVID-19 vs. general population	Mean_postcentral_thickness	Inverse variance weighted	24	0.0015	0.0019	0.4398	-0.0023	0.0052
Hospitalized COVID-19 vs. general population	Mean_posteriorcingulate_surface_area	MR Egger	24	-1.9829	6.2071	0.7524	-14.1487	10.1829
Hospitalized COVID-19 vs. general population	Mean_posteriorcingulate_surface_area	Weighted median	24	-0.1662	3.3283	0.9602	-6.6897	6.3573
Hospitalized COVID-19 vs. general population	Mean_posteriorcingulate_surface_area	Inverse variance weighted	24	1.4370	2.3503	0.5409	-3.1696	6.0437
Hospitalized COVID-19 vs. general population	Mean_posteriorcingulate_thickness	MR Egger	24	-0.0011	0.0098	0.9109	-0.0203	0.0181

continued:

COVID-19 phenotype	Brain structure	Method	nsnp	β	se	p value	β_low95%CI	β_up95%CI
Hospitalized COVID-19 vs. general population	Mean_posteriorcingulate_thickness	Weighted median	24	0.0020	0.0039	0.6103	-0.0056	0.0096
Hospitalized COVID-19 vs. general population	Mean_posteriorcingulate_thickness	Inverse variance weighted	24	-0.0001	0.0036	0.9769	-0.0072	0.0070
Hospitalized COVID-19 vs. general population	Mean_precentral_surface_area	MR Egger	24	-5.9646	18.1111	0.7450	-41.4623	29.5331
Hospitalized COVID-19 vs. general population	Mean_precentral_surface_area	Weighted median	24	19.0300	9.6352	0.0483	0.1449	37.9150
Hospitalized COVID-19 vs. general population	Mean_precentral_surface_area	Inverse variance weighted	24	14.3612	6.8580	0.0363	0.9196	27.8029
Hospitalized COVID-19 vs. general population	Mean_precentral_thickness	MR Egger	24	0.0073	0.0055	0.1929	-0.0034	0.0180
Hospitalized COVID-19 vs. general population	Mean_precentral_thickness	Weighted median	24	-0.0040	0.0030	0.1869	-0.0098	0.0019
Hospitalized COVID-19 vs. general population	Mean_precentral_thickness	Inverse variance weighted	24	-0.0033	0.0022	0.1387	-0.0076	0.0011
Hospitalized COVID-19 vs. general population	Mean_precuneus_surface_area	MR Egger	24	20.9403	17.1098	0.2339	-12.5949	54.4755
Hospitalized COVID-19 vs. general population	Mean_precuneus_surface_area	Weighted median	24	9.6114	8.6205	0.2649	-7.2847	26.5076
Hospitalized COVID-19 vs. general population	Mean_precuneus_surface_area	Inverse variance weighted	24	3.3415	6.5121	0.6079	-9.4222	16.1053
Hospitalized COVID-19 vs. general population	Mean_precuneus_thickness	MR Egger	24	0.0006	0.0049	0.9082	-0.0090	0.0102
Hospitalized COVID-19 vs. general population	Mean_precuneus_thickness	Weighted median	24	-0.0003	0.0025	0.9191	-0.0052	0.0047
Hospitalized COVID-19 vs. general population	Mean_precuneus_thickness	Inverse variance weighted	24	0.0005	0.0018	0.7708	-0.0030	0.0041
Hospitalized COVID-19 vs. general population	Mean_rostralanteriorcingulate_surface_area	MR Egger	24	2.3968	5.2414	0.6520	-7.8764	12.6700
Hospitalized COVID-19 vs. general population	Mean_rostralanteriorcingulate_surface_area	Weighted median	24	-3.1911	2.6745	0.2328	-8.4330	2.0509
Hospitalized COVID-19 vs. general population	Mean_rostralanteriorcingulate_surface_area	Inverse variance weighted	24	-2.3740	1.9837	0.2314	-6.2620	1.5140
Hospitalized COVID-19 vs. general population	Mean_rostralanteriorcingulate_thickness	MR Egger	24	-0.0076	0.0098	0.4464	-0.0269	0.0117
Hospitalized COVID-19 vs. general population	Mean_rostralanteriorcingulate_thickness	Weighted median	24	-0.0030	0.0051	0.5622	-0.0131	0.0071
Hospitalized COVID-19 vs. general population	Mean_rostralanteriorcingulate_thickness	Inverse variance weighted	24	-0.0025	0.0037	0.5063	-0.0097	0.0048
Hospitalized COVID-19 vs. general population	Mean_rostralmiddlefrontal_surface_area	MR Egger	24	17.8689	21.6900	0.4189	-24.6435	60.3813
Hospitalized COVID-19 vs. general population	Mean_rostralmiddlefrontal_surface_area	Weighted median	24	-5.4488	11.2918	0.6294	-27.5807	16.6832
Hospitalized COVID-19 vs. general population	Mean_rostralmiddlefrontal_surface_area	Inverse variance weighted	24	-6.4587	8.2110	0.4315	-22.5523	9.6349
Hospitalized COVID-19 vs. general population	Mean_rostralmiddlefrontal_thickness	MR Egger	24	-0.0088	0.0049	0.0845	-0.0184	0.0007
Hospitalized COVID-19 vs. general population	Mean_rostralmiddlefrontal_thickness	Weighted median	24	-0.0046	0.0025	0.0610	-0.0095	0.0002
Hospitalized COVID-19 vs. general population	Mean_rostralmiddlefrontal_thickness	Inverse variance weighted	24	-0.0027	0.0019	0.1540	-0.0064	0.0010
Hospitalized COVID-19 vs. general population	Mean_superiorfrontal_surface_area	MR Egger	24	8.1961	25.6449	0.7523	-42.0679	58.4600
Hospitalized COVID-19 vs. general population	Mean_superiorfrontal_surface_area	Weighted median	24	-9.9174	12.3024	0.4202	-34.0300	14.1953
Hospitalized COVID-19 vs. general population	Mean_superiorfrontal_surface_area	Inverse variance weighted	24	-5.7204	9.5742	0.5502	-24.4859	13.0451

continued:

COVID-19 phenotype	Brain structure	Method	nsnp	β	se	p value	β_low95%CI	β_up95%CI
Hospitalized COVID-19 vs. general population	Mean_superiorfrontal_thickness	MR Egger	24	-0.0059	0.0052	0.2711	-0.0161	0.0043
Hospitalized COVID-19 vs. general population	Mean_superiorfrontal_thickness	Weighted median	24	0.0009	0.0027	0.7416	-0.0044	0.0062
Hospitalized COVID-19 vs. general population	Mean_superiorfrontal_thickness	Inverse variance weighted	24	-0.0018	0.0020	0.3685	-0.0057	0.0021
Hospitalized COVID-19 vs. general population	Mean_superiorparietal_surface_area	MR Egger	24	4.8414	22.1714	0.8292	-38.6145	48.2973
Hospitalized COVID-19 vs. general population	Mean_superiorparietal_surface_area	Weighted median	24	-6.6746	11.6601	0.5670	-29.5284	16.1791
Hospitalized COVID-19 vs. general population	Mean_superiorparietal_surface_area	Inverse variance weighted	24	-5.8524	8.3872	0.4853	-22.2913	10.5866
Hospitalized COVID-19 vs. general population	Mean_superiorparietal_thickness	MR Egger	24	0.0000	0.0044	0.9979	-0.0085	0.0086
Hospitalized COVID-19 vs. general population	Mean_superiorparietal_thickness	Weighted median	24	0.0014	0.0023	0.5386	-0.0031	0.0059
Hospitalized COVID-19 vs. general population	Mean_superiorparietal_thickness	Inverse variance weighted	24	0.0005	0.0016	0.7483	-0.0027	0.0038
Hospitalized COVID-19 vs. general population	Mean_superiortemporal_surface_area	MR Egger	24	7.1204	12.8113	0.5840	-17.9897	32.2306
Hospitalized COVID-19 vs. general population	Mean_superiortemporal_surface_area	Weighted median	24	-3.6057	7.2368	0.6183	-17.7898	10.5784
Hospitalized COVID-19 vs. general population	Mean_superiortemporal_surface_area	Inverse variance weighted	24	-4.9112	4.8476	0.3110	-14.4125	4.5902
Hospitalized COVID-19 vs. general population	Mean_superiortemporal_thickness	MR Egger	24	0.0008	0.0062	0.9011	-0.0114	0.0130
Hospitalized COVID-19 vs. general population	Mean_superiortemporal_thickness	Weighted median	24	0.0009	0.0031	0.7634	-0.0052	0.0070
Hospitalized COVID-19 vs. general population	Mean_superiortemporal_thickness	Inverse variance weighted	24	0.0031	0.0023	0.1799	-0.0014	0.0076
Hospitalized COVID-19 vs. general population	Mean_supramarginal_surface_area	MR Egger	24	-24.9308	17.7247	0.1735	-59.6712	9.8095
Hospitalized COVID-19 vs. general population	Mean_supramarginal_surface_area	Weighted median	24	4.1844	9.2416	0.6507	-13.9291	22.2979
Hospitalized COVID-19 vs. general population	Mean_supramarginal_surface_area	Inverse variance weighted	24	2.6636	6.7115	0.6915	-10.4910	15.8183
Hospitalized COVID-19 vs. general population	Mean_supramarginal_thickness	MR Egger	24	0.0045	0.0043	0.3075	-0.0040	0.0130
Hospitalized COVID-19 vs. general population	Mean_supramarginal_thickness	Weighted median	24	0.0000	0.0022	1.0000	-0.0044	0.0044
Hospitalized COVID-19 vs. general population	Mean_supramarginal_thickness	Inverse variance weighted	24	-0.0012	0.0017	0.4764	-0.0045	0.0021
Hospitalized COVID-19 vs. general population	Mean_temporalpole_surface_area	MR Egger	24	-0.7995	2.9958	0.7920	-6.6713	5.0722
Hospitalized COVID-19 vs. general population	Mean_temporalpole_surface_area	Weighted median	24	0.9364	1.4879	0.5291	-1.9798	3.8527
Hospitalized COVID-19 vs. general population	Mean_temporalpole_surface_area	Inverse variance weighted	24	0.1364	1.1121	0.9024	-2.0433	2.3161
Hospitalized COVID-19 vs. general population	Mean_temporalpole_thickness	MR Egger	24	0.0045	0.0185	0.8091	-0.0318	0.0408
Hospitalized COVID-19 vs. general population	Mean_temporalpole_thickness	Weighted median	24	-0.0014	0.0084	0.8680	-0.0179	0.0151
Hospitalized COVID-19 vs. general population	Mean_temporalpole_thickness	Inverse variance weighted	24	0.0025	0.0069	0.7153	-0.0110	0.0160
Hospitalized COVID-19 vs. general population	Mean_transversetemporal_surface_area	MR Egger	24	0.7311	2.7562	0.7933	-4.6711	6.1332
Hospitalized COVID-19 vs. general population	Mean_transversetemporal_surface_area	Weighted median	24	0.3029	1.4884	0.8387	-2.6143	3.2202

continued:

COVID-19 phenotype	Brain structure	Method	nsnp	β	se	p value	β_low95%CI	β_up95%CI
Hospitalized COVID-19 vs. general population	Mean_transversetemporal_surface_area	Inverse variance weighted	24	0.5562	1.0437	0.5941	-1.4895	2.6018
Hospitalized COVID-19 vs. general population	Mean_transversetemporal_thickness	MR Egger	24	0.0082	0.0100	0.4187	-0.0113	0.0278
Hospitalized COVID-19 vs. general population	Mean_transversetemporal_thickness	Weighted median	24	-0.0063	0.0054	0.2468	-0.0169	0.0043
Hospitalized COVID-19 vs. general population	Mean_transversetemporal_thickness	Inverse variance weighted	24	-0.0057	0.0039	0.1395	-0.0133	0.0019
Hospitalized COVID-19 vs. general population	Mean_Amygdala_volume	MR Egger	23	8.5809	16.4043	0.6064	-23.5716	40.7334
Hospitalized COVID-19 vs. general population	Mean_Amygdala_volume	Weighted median	23	8.9233	9.0744	0.3254	-8.8626	26.7092
Hospitalized COVID-19 vs. general population	Mean_Amygdala_volume	Inverse variance weighted	23	5.9998	6.3946	0.3481	-6.5336	18.5332
Hospitalized COVID-19 vs. general population	Mean_Caudate_volume	MR Egger	23	-24.2666	33.7815	0.4805	-90.4782	41.9451
Hospitalized COVID-19 vs. general population	Mean_Caudate_volume	Weighted median	23	28.1494	19.0691	0.1399	-9.2261	65.5248
Hospitalized COVID-19 vs. general population	Mean_Caudate_volume	Inverse variance weighted	23	17.7378	13.5404	0.1902	-8.8013	44.2770
Hospitalized COVID-19 vs. general population	Mean_Hippocampus_volume	MR Egger	23	-10.2273	32.3448	0.7550	-73.6230	53.1685
Hospitalized COVID-19 vs. general population	Mean_Hippocampus_volume	Weighted median	23	21.0884	17.3336	0.2237	-12.8854	55.0623
Hospitalized COVID-19 vs. general population	Mean_Hippocampus_volume	Inverse variance weighted	23	20.5599	12.7017	0.1055	-4.3355	45.4553
Hospitalized COVID-19 vs. general population	Mean_Intracranial_volume	MR Egger	24	12648.3723	12533.6427	0.3239	-11917.5674	37214.3120
Hospitalized COVID-19 vs. general population	Mean_Intracranial_volume	Weighted median	24	1436.2967	7067.2928	0.8390	-12415.5973	15288.1907
Hospitalized COVID-19 vs. general population	Mean_Intracranial_volume	Inverse variance weighted	24	-1921.3180	5121.0021	0.7075	-11958.4822	8115.8462
Hospitalized COVID-19 vs. general population	Mean_Nucleus_accumbens_volume	MR Egger	23	5.3256	7.6246	0.4925	-9.6187	20.2699
Hospitalized COVID-19 vs. general population	Mean_Nucleus_accumbens_volume	Weighted median	23	1.8418	4.3061	0.6688	-6.5981	10.2818
Hospitalized COVID-19 vs. general population	Mean_Nucleus_accumbens_volume	Inverse variance weighted	23	1.0824	2.9813	0.7166	-4.7610	6.9257
Hospitalized COVID-19 vs. general population	Mean_Pallidum_volume	MR Egger	23	-5.0134	13.1466	0.7068	-30.7807	20.7540
Hospitalized COVID-19 vs. general population	Mean_Pallidum_volume	Weighted median	23	2.5635	7.0983	0.7180	-11.3491	16.4761
Hospitalized COVID-19 vs. general population	Mean_Pallidum_volume	Inverse variance weighted	23	5.7277	5.1725	0.2681	-4.4104	15.8659
Hospitalized COVID-19 vs. general population	Mean_Putamen_volume	MR Egger	23	-3.4642	40.9433	0.9334	-83.7132	76.7847
Hospitalized COVID-19 vs. general population	Mean_Putamen_volume	Weighted median	23	40.5939	22.5985	0.0724	-3.6990	84.8869
Hospitalized COVID-19 vs. general population	Mean_Putamen_volume	Inverse variance weighted	23	39.7260	16.1271	0.0138	8.1168	71.3352
Hospitalized COVID-19 vs. general population	Mean_Thalamus_volume	MR Egger	23	31.2308	43.0569	0.4763	-53.1607	115.6222
Hospitalized COVID-19 vs. general population	Mean_Thalamus_volume	Weighted median	23	-4.3422	25.3443	0.8640	-54.0170	45.3327
Hospitalized COVID-19 vs. general population	Mean_Thalamus_volume	Inverse variance weighted	23	2.7417	16.8867	0.8710	-30.3562	35.8395

Abbreviations: COVID-19, coronavirus disease 2019; CI, confidence interval; nSNPs, number of single-nucleotide polymorphisms; se, standard error

Table S4. MR analysis of the causal relationship between hospitalized COVID-19 (vs. non-hospitalized COVID-19) and brain structure

COVID-19 phenotype	Brain structure	Method	nsnp	β	se	p value	β_low95%CI	β_up95%CI
Hospitalized COVID-19 vs. non-hospitalized COVID-19	Mean_bankssts_surface_area	MR Egger	19	0.2393	2.3158	0.9189	-4.2996	4.7782
Hospitalized COVID-19 vs. non-hospitalized COVID-19	Mean_bankssts_surface_area	Weighted median	19	-0.3606	1.4400	0.8023	-3.1831	2.4619
Hospitalized COVID-19 vs. non-hospitalized COVID-19	Mean_bankssts_surface_area	Inverse variance weighted	19	-0.2174	1.0338	0.8334	-2.2436	1.8087
Hospitalized COVID-19 vs. non-hospitalized COVID-19	Mean_bankssts_thickness	MR Egger	19	-0.0015	0.0030	0.6129	-0.0073	0.0043
Hospitalized COVID-19 vs. non-hospitalized COVID-19	Mean_bankssts_thickness	Weighted median	19	0.0010	0.0017	0.5664	-0.0024	0.0044
Hospitalized COVID-19 vs. non-hospitalized COVID-19	Mean_bankssts_thickness	Inverse variance weighted	19	-0.0003	0.0013	0.8286	-0.0028	0.0022
Hospitalized COVID-19 vs. non-hospitalized COVID-19	Mean_caudalanteriorcingulate_surface_area	MR Egger	19	5.7099	2.2384	0.0207	1.3226	10.0971
Hospitalized COVID-19 vs. non-hospitalized COVID-19	Mean_caudalanteriorcingulate_surface_area	Weighted median	19	1.4638	1.3329	0.2721	-1.1487	4.0763
Hospitalized COVID-19 vs. non-hospitalized COVID-19	Mean_caudalanteriorcingulate_surface_area	Inverse variance weighted	19	0.5835	1.1407	0.6090	-1.6523	2.8192
Hospitalized COVID-19 vs. non-hospitalized COVID-19	Mean_caudalanteriorcingulate_thickness	MR Egger	19	0.0056	0.0043	0.2080	-0.0028	0.0140
Hospitalized COVID-19 vs. non-hospitalized COVID-19	Mean_caudalanteriorcingulate_thickness	Weighted median	19	0.0019	0.0027	0.4839	-0.0034	0.0072
Hospitalized COVID-19 vs. non-hospitalized COVID-19	Mean_caudalanteriorcingulate_thickness	Inverse variance weighted	19	0.0027	0.0019	0.1582	-0.0010	0.0064
Hospitalized COVID-19 vs. non-hospitalized COVID-19	Mean_caudalmiddlefrontal_surface_area	MR Egger	19	1.8737	5.4387	0.7347	-8.7861	12.5335
Hospitalized COVID-19 vs. non-hospitalized COVID-19	Mean_caudalmiddlefrontal_surface_area	Weighted median	19	-1.2945	3.5512	0.7155	-8.2548	5.6658
Hospitalized COVID-19 vs. non-hospitalized COVID-19	Mean_caudalmiddlefrontal_surface_area	Inverse variance weighted	19	0.6239	2.4279	0.7972	-4.1348	5.3827
Hospitalized COVID-19 vs. non-hospitalized COVID-19	Mean_caudalmiddlefrontal_thickness	MR Egger	19	0.0021	0.0025	0.4168	-0.0028	0.0069
Hospitalized COVID-19 vs. non-hospitalized COVID-19	Mean_caudalmiddlefrontal_thickness	Weighted median	19	0.0002	0.0013	0.8857	-0.0024	0.0028
Hospitalized COVID-19 vs. non-hospitalized COVID-19	Mean_caudalmiddlefrontal_thickness	Inverse variance weighted	19	-0.0004	0.0011	0.7111	-0.0026	0.0017
Hospitalized COVID-19 vs. non-hospitalized COVID-19	Mean_cuneus_surface_area	MR Egger	19	1.8352	3.7013	0.6264	-5.4195	9.0898
Hospitalized COVID-19 vs. non-hospitalized COVID-19	Mean_cuneus_surface_area	Weighted median	19	0.2218	2.0886	0.9154	-3.8718	4.3155
Hospitalized COVID-19 vs. non-hospitalized COVID-19	Mean_cuneus_surface_area	Inverse variance weighted	19	-0.3460	1.6237	0.8313	-3.5285	2.8365
Hospitalized COVID-19 vs. non-hospitalized COVID-19	Mean_cuneus_thickness	MR Egger	19	-0.0010	0.0031	0.7486	-0.0071	0.0051
Hospitalized COVID-19 vs. non-hospitalized COVID-19	Mean_cuneus_thickness	Weighted median	19	0.0010	0.0014	0.4811	-0.0018	0.0038
Hospitalized COVID-19 vs. non-hospitalized COVID-19	Mean_cuneus_thickness	Inverse variance weighted	19	0.0000	0.0013	0.9928	-0.0026	0.0026
Hospitalized COVID-19 vs. non-hospitalized COVID-19	Mean_entorhinal_surface_area	MR Egger	19	0.3837	1.3240	0.7755	-2.2114	2.9788
Hospitalized COVID-19 vs. non-hospitalized COVID-19	Mean_entorhinal_surface_area	Weighted median	19	0.4130	0.8137	0.6118	-1.1819	2.0078
Hospitalized COVID-19 vs. non-hospitalized COVID-19	Mean_entorhinal_surface_area	Inverse variance weighted	19	0.6010	0.5794	0.2996	-0.5347	1.7366
Hospitalized COVID-19 vs. non-hospitalized COVID-19	Mean_entorhinal_thickness	MR Egger	19	-0.0084	0.0066	0.2194	-0.0213	0.0045
Hospitalized COVID-19 vs. non-hospitalized COVID-19	Mean_entorhinal_thickness	Weighted median	19	-0.0001	0.0041	0.9743	-0.0082	0.0080
Hospitalized COVID-19 vs. non-hospitalized COVID-19	Mean_entorhinal_thickness	Inverse variance weighted	19	0.0007	0.0031	0.8283	-0.0053	0.0066

continued:

COVID-19 phenotype	Brain structure	Method	nsnp	β	se	p value	β_low95%CI	β_up95%CI
Hospitalized COVID-19 vs. non-hospitalized COVID-19	Mean_frontalpole_surface_area	MR Egger	19	-0.9560	0.6581	0.1645	-2.2459	0.3338
Hospitalized COVID-19 vs. non-hospitalized COVID-19	Mean_frontalpole_surface_area	Weighted median	19	0.0845	0.3927	0.8297	-0.6853	0.8542
Hospitalized COVID-19 vs. non-hospitalized COVID-19	Mean_frontalpole_surface_area	Inverse variance weighted	19	-0.2492	0.2973	0.4020	-0.8320	0.3336
Hospitalized COVID-19 vs. non-hospitalized COVID-19	Mean_frontalpole_thickness	MR Egger	20	-0.0072	0.0048	0.1537	-0.0167	0.0023
Hospitalized COVID-19 vs. non-hospitalized COVID-19	Mean_frontalpole_thickness	Weighted median	20	-0.0039	0.0028	0.1595	-0.0094	0.0016
Hospitalized COVID-19 vs. non-hospitalized COVID-19	Mean_frontalpole_thickness	Inverse variance weighted	20	-0.0031	0.0020	0.1170	-0.0070	0.0008
Hospitalized COVID-19 vs. non-hospitalized COVID-19	Mean_full_surface_area	MR Egger	3	121.4124	922.2372	0.9167	-1686.1725	1928.9973
Hospitalized COVID-19 vs. non-hospitalized COVID-19	Mean_full_surface_area	Weighted median	3	-112.0069	391.8327	0.7750	-879.9991	655.9853
Hospitalized COVID-19 vs. non-hospitalized COVID-19	Mean_full_surface_area	Inverse variance weighted	3	-56.0671	334.0620	0.8667	-710.8287	598.6944
Hospitalized COVID-19 vs. non-hospitalized COVID-19	Mean_full_thickness	MR Egger	7	-0.0028	0.0055	0.6357	-0.0135	0.0080
Hospitalized COVID-19 vs. non-hospitalized COVID-19	Mean_full_thickness	Weighted median	7	-0.0016	0.0020	0.4388	-0.0056	0.0024
Hospitalized COVID-19 vs. non-hospitalized COVID-19	Mean_full_thickness	Inverse variance weighted	7	-0.0012	0.0017	0.4855	-0.0045	0.0022
Hospitalized COVID-19 vs. non-hospitalized COVID-19	Mean_fusiform_surface_area	MR Egger	19	-1.4866	5.1890	0.7780	-11.6571	8.6838
Hospitalized COVID-19 vs. non-hospitalized COVID-19	Mean_fusiform_surface_area	Weighted median	19	-5.7250	3.1271	0.0671	-11.8541	0.4041
Hospitalized COVID-19 vs. non-hospitalized COVID-19	Mean_fusiform_surface_area	Inverse variance weighted	19	-2.8457	2.3120	0.2184	-7.3773	1.6858
Hospitalized COVID-19 vs. non-hospitalized COVID-19	Mean_fusiform_thickness	MR Egger	19	0.0016	0.0024	0.5011	-0.0030	0.0063
Hospitalized COVID-19 vs. non-hospitalized COVID-19	Mean_fusiform_thickness	Weighted median	19	0.0012	0.0014	0.3924	-0.0015	0.0039
Hospitalized COVID-19 vs. non-hospitalized COVID-19	Mean_fusiform_thickness	Inverse variance weighted	19	0.0010	0.0010	0.3104	-0.0010	0.0030
Hospitalized COVID-19 vs. non-hospitalized COVID-19	Mean_inferiorparietal_surface_area	MR Egger	19	-15.1490	11.1466	0.1919	-36.9963	6.6984
Hospitalized COVID-19 vs. non-hospitalized COVID-19	Mean_inferiorparietal_surface_area	Weighted median	19	2.5215	6.0007	0.6743	-9.2399	14.2829
Hospitalized COVID-19 vs. non-hospitalized COVID-19	Mean_inferiorparietal_surface_area	Inverse variance weighted	19	1.2633	5.2051	0.8082	-8.9388	11.4654
Hospitalized COVID-19 vs. non-hospitalized COVID-19	Mean_inferiorparietal_thickness	MR Egger	19	-0.0028	0.0016	0.0927	-0.0059	0.0003
Hospitalized COVID-19 vs. non-hospitalized COVID-19	Mean_inferiorparietal_thickness	Weighted median	19	-0.0001	0.0010	0.8903	-0.0021	0.0018
Hospitalized COVID-19 vs. non-hospitalized COVID-19	Mean_inferiorparietal_thickness	Inverse variance weighted	19	-0.0003	0.0007	0.6207	-0.0017	0.0010
Hospitalized COVID-19 vs. non-hospitalized COVID-19	Mean_inferiortemporal_surface_area	MR Egger	19	6.7391	5.8766	0.2674	-4.7791	18.2573
Hospitalized COVID-19 vs. non-hospitalized COVID-19	Mean_inferiortemporal_surface_area	Weighted median	19	1.2330	3.5184	0.7260	-5.6631	8.1291
Hospitalized COVID-19 vs. non-hospitalized COVID-19	Mean_inferiortemporal_surface_area	Inverse variance weighted	19	2.6388	2.6221	0.3142	-2.5005	7.7782
Hospitalized COVID-19 vs. non-hospitalized COVID-19	Mean_inferiortemporal_thickness	MR Egger	19	0.0028	0.0027	0.3211	-0.0025	0.0081
Hospitalized COVID-19 vs. non-hospitalized COVID-19	Mean_inferiortemporal_thickness	Weighted median	19	-0.0015	0.0016	0.3459	-0.0045	0.0016
Hospitalized COVID-19 vs. non-hospitalized COVID-19	Mean_inferiortemporal_thickness	Inverse variance weighted	19	-0.0017	0.0013	0.1798	-0.0042	0.0008

continued:

COVID-19 phenotype	Brain structure	Method	nsnp	β	se	p value	β_low95%CI	β_up95%CI
Hospitalized COVID-19 vs. non-hospitalized COVID-19	Mean_insula_surface_area	MR Egger	19	-6.3013	3.5055	0.0900	-13.1720	0.5694
Hospitalized COVID-19 vs. non-hospitalized COVID-19	Mean_insula_surface_area	Weighted median	19	-2.2926	2.1010	0.2752	-6.4107	1.8254
Hospitalized COVID-19 vs. non-hospitalized COVID-19	Mean_insula_surface_area	Inverse variance weighted	19	-3.0180	1.5660	0.0540	-6.0874	0.0514
Hospitalized COVID-19 vs. non-hospitalized COVID-19	Mean_insula_thickness	MR Egger	19	-0.0022	0.0025	0.3958	-0.0072	0.0028
Hospitalized COVID-19 vs. non-hospitalized COVID-19	Mean_insula_thickness	Weighted median	19	0.0004	0.0017	0.8100	-0.0029	0.0037
Hospitalized COVID-19 vs. non-hospitalized COVID-19	Mean_insula_thickness	Inverse variance weighted	19	0.0016	0.0012	0.1785	-0.0007	0.0039
Hospitalized COVID-19 vs. non-hospitalized COVID-19	Mean_isthmuscingulate_surface_area	MR Egger	19	-6.7516	2.3723	0.0112	-11.4014	-2.1019
Hospitalized COVID-19 vs. non-hospitalized COVID-19	Mean_isthmuscingulate_surface_area	Weighted median	19	0.7844	1.6010	0.6242	-2.3535	3.9223
Hospitalized COVID-19 vs. non-hospitalized COVID-19	Mean_isthmuscingulate_surface_area	Inverse variance weighted	19	0.8851	1.3047	0.4975	-1.6721	3.4423
Hospitalized COVID-19 vs. non-hospitalized COVID-19	Mean_isthmuscingulate_thickness	MR Egger	19	0.0041	0.0036	0.2719	-0.0030	0.0112
Hospitalized COVID-19 vs. non-hospitalized COVID-19	Mean_isthmuscingulate_thickness	Weighted median	19	-0.0022	0.0022	0.3143	-0.0066	0.0021
Hospitalized COVID-19 vs. non-hospitalized COVID-19	Mean_isthmuscingulate_thickness	Inverse variance weighted	19	-0.0012	0.0016	0.4641	-0.0043	0.0020
Hospitalized COVID-19 vs. non-hospitalized COVID-19	Mean_lateraloccipital_surface_area	MR Egger	19	4.1259	7.7914	0.6033	-11.1453	19.3970
Hospitalized COVID-19 vs. non-hospitalized COVID-19	Mean_lateraloccipital_surface_area	Weighted median	19	-1.4575	4.5849	0.7506	-10.4439	7.5289
Hospitalized COVID-19 vs. non-hospitalized COVID-19	Mean_lateraloccipital_surface_area	Inverse variance weighted	19	-1.3937	3.4756	0.6884	-8.2059	5.4185
Hospitalized COVID-19 vs. non-hospitalized COVID-19	Mean_lateraloccipital_thickness	MR Egger	19	0.0033	0.0019	0.0956	-0.0004	0.0071
Hospitalized COVID-19 vs. non-hospitalized COVID-19	Mean_lateraloccipital_thickness	Weighted median	19	0.0010	0.0011	0.3720	-0.0012	0.0033
Hospitalized COVID-19 vs. non-hospitalized COVID-19	Mean_lateraloccipital_thickness	Inverse variance weighted	19	0.0005	0.0008	0.5907	-0.0012	0.0021
Hospitalized COVID-19 vs. non-hospitalized COVID-19	Mean_lateralorbitofrontal_surface_area	MR Egger	19	1.0773	4.9058	0.8288	-8.5381	10.6926
Hospitalized COVID-19 vs. non-hospitalized COVID-19	Mean_lateralorbitofrontal_surface_area	Weighted median	19	2.2807	2.3363	0.3290	-2.2984	6.8598
Hospitalized COVID-19 vs. non-hospitalized COVID-19	Mean_lateralorbitofrontal_surface_area	Inverse variance weighted	19	2.3358	2.1338	0.2737	-1.8465	6.5181
Hospitalized COVID-19 vs. non-hospitalized COVID-19	Mean_lateralorbitofrontal_thickness	MR Egger	19	0.0013	0.0024	0.5871	-0.0034	0.0061
Hospitalized COVID-19 vs. non-hospitalized COVID-19	Mean_lateralorbitofrontal_thickness	Weighted median	19	0.0000	0.0015	0.9853	-0.0029	0.0029
Hospitalized COVID-19 vs. non-hospitalized COVID-19	Mean_lateralorbitofrontal_thickness	Inverse variance weighted	19	-0.0007	0.0011	0.5315	-0.0028	0.0014
Hospitalized COVID-19 vs. non-hospitalized COVID-19	Mean_lingual_surface_area	MR Egger	19	8.2918	7.7544	0.2999	-6.9069	23.4905
Hospitalized COVID-19 vs. non-hospitalized COVID-19	Mean_lingual_surface_area	Weighted median	19	3.7590	4.0422	0.3524	-4.1638	11.6817
Hospitalized COVID-19 vs. non-hospitalized COVID-19	Mean_lingual_surface_area	Inverse variance weighted	19	4.2509	3.3990	0.2111	-2.4110	10.9129
Hospitalized COVID-19 vs. non-hospitalized COVID-19	Mean_lingual_thickness	MR Egger	19	0.0014	0.0020	0.4963	-0.0025	0.0053
Hospitalized COVID-19 vs. non-hospitalized COVID-19	Mean_lingual_thickness	Weighted median	19	0.0001	0.0012	0.9160	-0.0022	0.0024

continued:

COVID-19 phenotype	Brain structure	Method	nsnp	β	se	p value	β_low95%CI	β_up95%CI
Hospitalized COVID-19 vs. non-hospitalized COVID-19	Mean_lingual_thickness	Inverse variance weighted	19	0.0005	0.0009	0.5801	-0.0012	0.0022
Hospitalized COVID-19 vs. non-hospitalized COVID-19	Mean_medialorbitofrontal_surface_area	MR Egger	19	-0.9636	3.2471	0.7703	-7.3279	5.4008
Hospitalized COVID-19 vs. non-hospitalized COVID-19	Mean_medialorbitofrontal_surface_area	Weighted median	19	-0.4742	1.8945	0.8024	-4.1874	3.2391
Hospitalized COVID-19 vs. non-hospitalized COVID-19	Mean_medialorbitofrontal_surface_area	Inverse variance weighted	19	-0.5573	1.4081	0.6923	-3.3171	2.2026
Hospitalized COVID-19 vs. non-hospitalized COVID-19	Mean_medialorbitofrontal_thickness	MR Egger	19	-0.0010	0.0028	0.7156	-0.0065	0.0045
Hospitalized COVID-19 vs. non-hospitalized COVID-19	Mean_medialorbitofrontal_thickness	Weighted median	19	-0.0012	0.0017	0.4894	-0.0044	0.0021
Hospitalized COVID-19 vs. non-hospitalized COVID-19	Mean_medialorbitofrontal_thickness	Inverse variance weighted	19	-0.0009	0.0012	0.4927	-0.0033	0.0016
Hospitalized COVID-19 vs. non-hospitalized COVID-19	Mean_middletemporal_surface_area	MR Egger	19	3.5741	5.0658	0.4900	-6.3549	13.5031
Hospitalized COVID-19 vs. non-hospitalized COVID-19	Mean_middletemporal_surface_area	Weighted median	19	3.0393	3.0845	0.3245	-3.0063	9.0849
Hospitalized COVID-19 vs. non-hospitalized COVID-19	Mean_middletemporal_surface_area	Inverse variance weighted	19	3.4057	2.2586	0.1316	-1.0211	7.8325
Hospitalized COVID-19 vs. non-hospitalized COVID-19	Mean_middletemporal_thickness	MR Egger	19	-0.0005	0.0028	0.8748	-0.0060	0.0051
Hospitalized COVID-19 vs. non-hospitalized COVID-19	Mean_middletemporal_thickness	Weighted median	19	-0.0019	0.0015	0.1954	-0.0048	0.0010
Hospitalized COVID-19 vs. non-hospitalized COVID-19	Mean_middletemporal_thickness	Inverse variance weighted	19	-0.0020	0.0012	0.1003	-0.0044	0.0004
Hospitalized COVID-19 vs. non-hospitalized COVID-19	Mean_paracentral_surface_area	MR Egger	19	1.1245	3.0904	0.7204	-4.9328	7.1817
Hospitalized COVID-19 vs. non-hospitalized COVID-19	Mean_paracentral_surface_area	Weighted median	19	-0.2798	1.7988	0.8764	-3.8055	3.2460
Hospitalized COVID-19 vs. non-hospitalized COVID-19	Mean_paracentral_surface_area	Inverse variance weighted	19	0.5877	1.3756	0.6692	-2.1084	3.2839
Hospitalized COVID-19 vs. non-hospitalized COVID-19	Mean_paracentral_thickness	MR Egger	19	-0.0045	0.0023	0.0721	-0.0091	0.0001
Hospitalized COVID-19 vs. non-hospitalized COVID-19	Mean_paracentral_thickness	Weighted median	19	0.0013	0.0015	0.3798	-0.0016	0.0041
Hospitalized COVID-19 vs. non-hospitalized COVID-19	Mean_paracentral_thickness	Inverse variance weighted	19	0.0006	0.0011	0.5652	-0.0015	0.0028
Hospitalized COVID-19 vs. non-hospitalized COVID-19	Mean_parahippocampal_surface_area	MR Egger	16	-0.7195	1.7113	0.6805	-4.0737	2.6347
Hospitalized COVID-19 vs. non-hospitalized COVID-19	Mean_parahippocampal_surface_area	Weighted median	16	-0.8908	1.0190	0.3820	-2.8880	1.1064
Hospitalized COVID-19 vs. non-hospitalized COVID-19	Mean_parahippocampal_surface_area	Inverse variance weighted	16	-1.0141	0.7488	0.1756	-2.4817	0.4535
Hospitalized COVID-19 vs. non-hospitalized COVID-19	Mean_parahippocampal_thickness	MR Egger	19	0.0056	0.0058	0.3497	-0.0058	0.0169
Hospitalized COVID-19 vs. non-hospitalized COVID-19	Mean_parahippocampal_thickness	Weighted median	19	0.0033	0.0036	0.3595	-0.0038	0.0104
Hospitalized COVID-19 vs. non-hospitalized COVID-19	Mean_parahippocampal_thickness	Inverse variance weighted	19	0.0029	0.0025	0.2543	-0.0021	0.0079
Hospitalized COVID-19 vs. non-hospitalized COVID-19	Mean_parsopercularis_surface_area	MR Egger	19	1.3111	4.2147	0.7595	-6.9497	9.5719
Hospitalized COVID-19 vs. non-hospitalized COVID-19	Mean_parsopercularis_surface_area	Weighted median	19	-1.2903	2.3646	0.5853	-5.9248	3.3442
Hospitalized COVID-19 vs. non-hospitalized COVID-19	Mean_parsopercularis_surface_area	Inverse variance weighted	19	-1.0473	1.8509	0.5715	-4.6750	2.5804
Hospitalized COVID-19 vs. non-hospitalized COVID-19	Mean_parsopercularis_thickness	MR Egger	19	0.0008	0.0020	0.6920	-0.0031	0.0046
Hospitalized COVID-19 vs. non-hospitalized COVID-19	Mean_parsopercularis_thickness	Weighted median	19	0.0004	0.0012	0.7679	-0.0021	0.0028

continued:

COVID-19 phenotype	Brain structure	Method	nsnp	β	se	p value	β_low95%CI	β_up95%CI
Hospitalized COVID-19 vs. non-hospitalized COVID-19	Mean_parsopercularis_thickness	Inverse variance weighted	19	-0.0008	0.0009	0.3451	-0.0026	0.0009
Hospitalized COVID-19 vs. non-hospitalized COVID-19	Mean_parsorbitalis_surface_area	MR Egger	19	-0.5031	1.6489	0.7640	-3.7349	2.7287
Hospitalized COVID-19 vs. non-hospitalized COVID-19	Mean_parsorbitalis_surface_area	Weighted median	19	-0.5337	0.7983	0.5038	-2.0983	1.0310
Hospitalized COVID-19 vs. non-hospitalized COVID-19	Mean_parsorbitalis_surface_area	Inverse variance weighted	19	0.1097	0.7192	0.8788	-1.2999	1.5193
Hospitalized COVID-19 vs. non-hospitalized COVID-19	Mean_parsorbitalis_thickness	MR Egger	19	0.0004	0.0034	0.8956	-0.0062	0.0071
Hospitalized COVID-19 vs. non-hospitalized COVID-19	Mean_parsorbitalis_thickness	Weighted median	19	0.0005	0.0020	0.8049	-0.0034	0.0044
Hospitalized COVID-19 vs. non-hospitalized COVID-19	Mean_parsorbitalis_thickness	Inverse variance weighted	19	-0.0017	0.0015	0.2543	-0.0046	0.0012
Hospitalized COVID-19 vs. non-hospitalized COVID-19	Mean_parstriangularis_surface_area	MR Egger	19	-0.4487	3.3600	0.8953	-7.0344	6.1369
Hospitalized COVID-19 vs. non-hospitalized COVID-19	Mean_parstriangularis_surface_area	Weighted median	19	-1.4507	2.1187	0.4935	-5.6034	2.7019
Hospitalized COVID-19 vs. non-hospitalized COVID-19	Mean_parstriangularis_surface_area	Inverse variance weighted	19	-1.6551	1.5020	0.2705	-4.5990	1.2889
Hospitalized COVID-19 vs. non-hospitalized COVID-19	Mean_parstriangularis_thickness	MR Egger	19	-0.0007	0.0021	0.7391	-0.0049	0.0035
Hospitalized COVID-19 vs. non-hospitalized COVID-19	Mean_parstriangularis_thickness	Weighted median	19	-0.0008	0.0013	0.5633	-0.0033	0.0018
Hospitalized COVID-19 vs. non-hospitalized COVID-19	Mean_parstriangularis_thickness	Inverse variance weighted	19	-0.0007	0.0009	0.4720	-0.0025	0.0012
Hospitalized COVID-19 vs. non-hospitalized COVID-19	Mean_pericalcarine_surface_area	MR Egger	19	3.0194	4.8604	0.5427	-6.5071	12.5458
Hospitalized COVID-19 vs. non-hospitalized COVID-19	Mean_pericalcarine_surface_area	Weighted median	19	2.2756	2.5643	0.3749	-2.7504	7.3016
Hospitalized COVID-19 vs. non-hospitalized COVID-19	Mean_pericalcarine_surface_area	Inverse variance weighted	19	1.2261	2.1201	0.5631	-2.9293	5.3814
Hospitalized COVID-19 vs. non-hospitalized COVID-19	Mean_pericalcarine_thickness	MR Egger	19	0.0012	0.0024	0.6329	-0.0036	0.0059
Hospitalized COVID-19 vs. non-hospitalized COVID-19	Mean_pericalcarine_thickness	Weighted median	19	-0.0011	0.0014	0.4584	-0.0038	0.0017
Hospitalized COVID-19 vs. non-hospitalized COVID-19	Mean_pericalcarine_thickness	Inverse variance weighted	19	-0.0001	0.0011	0.9618	-0.0021	0.0020
Hospitalized COVID-19 vs. non-hospitalized COVID-19	Mean_postcentral_surface_area	MR Egger	19	-1.7458	5.9250	0.7718	-13.3589	9.8672
Hospitalized COVID-19 vs. non-hospitalized COVID-19	Mean_postcentral_surface_area	Weighted median	19	-3.1953	3.4865	0.3594	-10.0289	3.6382
Hospitalized COVID-19 vs. non-hospitalized COVID-19	Mean_postcentral_surface_area	Inverse variance weighted	19	-3.6040	2.6361	0.1716	-8.7706	1.5627
Hospitalized COVID-19 vs. non-hospitalized COVID-19	Mean_postcentral_thickness	MR Egger	19	-0.0016	0.0019	0.3899	-0.0053	0.0020
Hospitalized COVID-19 vs. non-hospitalized COVID-19	Mean_postcentral_thickness	Weighted median	19	-0.0011	0.0011	0.3377	-0.0033	0.0011
Hospitalized COVID-19 vs. non-hospitalized COVID-19	Mean_postcentral_thickness	Inverse variance weighted	19	-0.0005	0.0008	0.5819	-0.0021	0.0012
Hospitalized COVID-19 vs. non-hospitalized COVID-19	Mean_posteriorcingulate_surface_area	MR Egger	19	0.9579	3.2734	0.7734	-5.4580	7.3737
Hospitalized COVID-19 vs. non-hospitalized COVID-19	Mean_posteriorcingulate_surface_area	Weighted median	19	-1.1780	1.6094	0.4642	-4.3324	1.9763
Hospitalized COVID-19 vs. non-hospitalized COVID-19	Mean_posteriorcingulate_surface_area	Inverse variance weighted	19	-1.3878	1.4438	0.3364	-4.2178	1.4421
Hospitalized COVID-19 vs. non-hospitalized COVID-19	Mean_posteriorcingulate_thickness	MR Egger	19	0.0013	0.0025	0.6158	-0.0036	0.0062
Hospitalized COVID-19 vs. non-hospitalized COVID-19	Mean_posteriorcingulate_thickness	Weighted median	19	0.0009	0.0016	0.5687	-0.0022	0.0039

continued:

COVID-19 phenotype	Brain structure	Method	nsnp	β	se	p value	β_low95%CI	β_up95%CI
Hospitalized COVID-19 vs. non-hospitalized COVID-19	Mean_posteriorcingulate_thickness	Inverse variance weighted	19	0.0012	0.0011	0.3004	-0.0010	0.0033
Hospitalized COVID-19 vs. non-hospitalized COVID-19	Mean_precentral_surface_area	MR Egger	19	1.7377	6.9336	0.8051	-11.8522	15.3277
Hospitalized COVID-19 vs. non-hospitalized COVID-19	Mean_precentral_surface_area	Weighted median	19	3.2561	4.3259	0.4516	-5.2225	11.7348
Hospitalized COVID-19 vs. non-hospitalized COVID-19	Mean_precentral_surface_area	Inverse variance weighted	19	4.6553	3.0898	0.1319	-1.4007	10.7113
Hospitalized COVID-19 vs. non-hospitalized COVID-19	Mean_precentral_thickness	MR Egger	19	0.0007	0.0023	0.7707	-0.0037	0.0051
Hospitalized COVID-19 vs. non-hospitalized COVID-19	Mean_precentral_thickness	Weighted median	19	0.0005	0.0013	0.7103	-0.0020	0.0030
Hospitalized COVID-19 vs. non-hospitalized COVID-19	Mean_precentral_thickness	Inverse variance weighted	19	0.0007	0.0010	0.4880	-0.0012	0.0025
Hospitalized COVID-19 vs. non-hospitalized COVID-19	Mean_precuneus_surface_area	MR Egger	19	-0.0312	6.4727	0.9962	-12.7178	12.6554
Hospitalized COVID-19 vs. non-hospitalized COVID-19	Mean_precuneus_surface_area	Weighted median	19	2.0398	3.7841	0.5898	-5.3770	9.4567
Hospitalized COVID-19 vs. non-hospitalized COVID-19	Mean_precuneus_surface_area	Inverse variance weighted	19	4.8783	2.8594	0.0880	-0.7261	10.4826
Hospitalized COVID-19 vs. non-hospitalized COVID-19	Mean_precuneus_thickness	MR Egger	20	-0.0034	0.0021	0.1203	-0.0074	0.0007
Hospitalized COVID-19 vs. non-hospitalized COVID-19	Mean_precuneus_thickness	Weighted median	20	-0.0006	0.0011	0.5524	-0.0027	0.0014
Hospitalized COVID-19 vs. non-hospitalized COVID-19	Mean_precuneus_thickness	Inverse variance weighted	20	-0.0015	0.0008	0.0822	-0.0031	0.0002
Hospitalized COVID-19 vs. non-hospitalized COVID-19	Mean_rostralanteriorcingulate_surface_area	MR Egger	19	2.8878	2.0099	0.1689	-1.0516	6.8273
Hospitalized COVID-19 vs. non-hospitalized COVID-19	Mean_rostralanteriorcingulate_surface_area	Weighted median	19	1.4228	1.2315	0.2479	-0.9908	3.8365
Hospitalized COVID-19 vs. non-hospitalized COVID-19	Mean_rostralanteriorcingulate_surface_area	Inverse variance weighted	19	1.0919	0.8950	0.2224	-0.6622	2.8460
Hospitalized COVID-19 vs. non-hospitalized COVID-19	Mean_rostralanteriorcingulate_thickness	MR Egger	19	-0.0046	0.0040	0.2592	-0.0124	0.0031
Hospitalized COVID-19 vs. non-hospitalized COVID-19	Mean_rostralanteriorcingulate_thickness	Weighted median	19	0.0016	0.0023	0.4895	-0.0029	0.0061
Hospitalized COVID-19 vs. non-hospitalized COVID-19	Mean_rostralanteriorcingulate_thickness	Inverse variance weighted	19	0.0006	0.0018	0.7295	-0.0029	0.0042
Hospitalized COVID-19 vs. non-hospitalized COVID-19	Mean_rostralmiddlefrontal_surface_area	MR Egger	19	1.9730	8.2910	0.8148	-14.2773	18.2232
Hospitalized COVID-19 vs. non-hospitalized COVID-19	Mean_rostralmiddlefrontal_surface_area	Weighted median	19	1.2841	5.0133	0.7978	-8.5419	11.1100
Hospitalized COVID-19 vs. non-hospitalized COVID-19	Mean_rostralmiddlefrontal_surface_area	Inverse variance weighted	19	1.2607	3.6906	0.7327	-5.9730	8.4943
Hospitalized COVID-19 vs. non-hospitalized COVID-19	Mean_rostralmiddlefrontal_thickness	MR Egger	20	-0.0017	0.0019	0.3739	-0.0054	0.0020
Hospitalized COVID-19 vs. non-hospitalized COVID-19	Mean_rostralmiddlefrontal_thickness	Weighted median	20	-0.0014	0.0011	0.1928	-0.0036	0.0007
Hospitalized COVID-19 vs. non-hospitalized COVID-19	Mean_rostralmiddlefrontal_thickness	Inverse variance weighted	20	-0.0022	0.0008	0.0032	-0.0037	-0.0008
Hospitalized COVID-19 vs. non-hospitalized COVID-19	Mean_superiorfrontal_surface_area	MR Egger	19	-4.9617	8.3358	0.5595	-21.2999	11.3766
Hospitalized COVID-19 vs. non-hospitalized COVID-19	Mean_superiorfrontal_surface_area	Weighted median	19	4.3687	5.2528	0.4056	-5.9268	14.6642
Hospitalized COVID-19 vs. non-hospitalized COVID-19	Mean_superiorfrontal_surface_area	Inverse variance weighted	19	1.9576	3.7142	0.5982	-5.3222	9.2375
Hospitalized COVID-19 vs. non-hospitalized COVID-19	Mean_superiorfrontal_thickness	MR Egger	19	0.0013	0.0019	0.4952	-0.0024	0.0050
Hospitalized COVID-19 vs. non-hospitalized COVID-19	Mean_superiorfrontal_thickness	Weighted median	19	-0.0006	0.0013	0.6584	-0.0030	0.0019

continued:

COVID-19 phenotype	Brain structure	Method	nsnp	β	se	p value	β_low95%CI	β_up95%CI
Hospitalized COVID-19 vs. non-hospitalized COVID-19	Mean_superiorfrontal_thickness	Inverse variance weighted	19	-0.0008	0.0008	0.3414	-0.0025	0.0009
Hospitalized COVID-19 vs. non-hospitalized COVID-19	Mean_superiorparietal_surface_area	MR Egger	19	-5.4099	8.4624	0.5312	-21.9963	11.1765
Hospitalized COVID-19 vs. non-hospitalized COVID-19	Mean_superiorparietal_surface_area	Weighted median	19	-5.3599	5.1276	0.2959	-15.4101	4.6902
Hospitalized COVID-19 vs. non-hospitalized COVID-19	Mean_superiorparietal_surface_area	Inverse variance weighted	19	-4.0362	3.7729	0.2847	-11.4310	3.3587
Hospitalized COVID-19 vs. non-hospitalized COVID-19	Mean_superiorparietal_thickness	MR Egger	19	-0.0001	0.0017	0.9506	-0.0034	0.0032
Hospitalized COVID-19 vs. non-hospitalized COVID-19	Mean_superiorparietal_thickness	Weighted median	19	0.0005	0.0010	0.6345	-0.0015	0.0025
Hospitalized COVID-19 vs. non-hospitalized COVID-19	Mean_superiorparietal_thickness	Inverse variance weighted	19	0.0000	0.0008	0.9726	-0.0015	0.0015
Hospitalized COVID-19 vs. non-hospitalized COVID-19	Mean_superiortemporal_surface_area	MR Egger	19	7.4607	6.8002	0.2879	-5.8676	20.7890
Hospitalized COVID-19 vs. non-hospitalized COVID-19	Mean_superiortemporal_surface_area	Weighted median	19	-4.1339	3.0008	0.1683	-10.0155	1.7477
Hospitalized COVID-19 vs. non-hospitalized COVID-19	Mean_superiortemporal_surface_area	Inverse variance weighted	19	-6.5261	3.3783	0.0534	-13.1475	0.0953
Hospitalized COVID-19 vs. non-hospitalized COVID-19	Mean_superiortemporal_thickness	MR Egger	19	-0.0030	0.0025	0.2508	-0.0079	0.0019
Hospitalized COVID-19 vs. non-hospitalized COVID-19	Mean_superiortemporal_thickness	Weighted median	19	0.0013	0.0014	0.3639	-0.0015	0.0041
Hospitalized COVID-19 vs. non-hospitalized COVID-19	Mean_superiortemporal_thickness	Inverse variance weighted	19	0.0014	0.0012	0.2541	-0.0010	0.0037
Hospitalized COVID-19 vs. non-hospitalized COVID-19	Mean_supramarginal_surface_area	MR Egger	19	-5.9193	6.7629	0.3936	-19.1746	7.3360
Hospitalized COVID-19 vs. non-hospitalized COVID-19	Mean_supramarginal_surface_area	Weighted median	19	3.3313	3.9958	0.4045	-4.5005	11.1632
Hospitalized COVID-19 vs. non-hospitalized COVID-19	Mean_supramarginal_surface_area	Inverse variance weighted	19	0.4459	3.0180	0.8825	-5.4694	6.3612
Hospitalized COVID-19 vs. non-hospitalized COVID-19	Mean_supramarginal_thickness	MR Egger	19	-0.0038	0.0019	0.0602	-0.0074	-0.0001
Hospitalized COVID-19 vs. non-hospitalized COVID-19	Mean_supramarginal_thickness	Weighted median	19	-0.0004	0.0010	0.6564	-0.0024	0.0015
Hospitalized COVID-19 vs. non-hospitalized COVID-19	Mean_supramarginal_thickness	Inverse variance weighted	19	-0.0005	0.0009	0.5990	-0.0022	0.0013
Hospitalized COVID-19 vs. non-hospitalized COVID-19	Mean_temporalpole_surface_area	MR Egger	19	-0.5640	0.9851	0.5744	-2.4948	1.3667
Hospitalized COVID-19 vs. non-hospitalized COVID-19	Mean_temporalpole_surface_area	Weighted median	19	-0.4124	0.6187	0.5050	-1.6251	0.8003
Hospitalized COVID-19 vs. non-hospitalized COVID-19	Mean_temporalpole_surface_area	Inverse variance weighted	19	0.0402	0.4399	0.9272	-0.8220	0.9024
Hospitalized COVID-19 vs. non-hospitalized COVID-19	Mean_temporalpole_thickness	MR Egger	19	-0.0025	0.0060	0.6855	-0.0143	0.0093
Hospitalized COVID-19 vs. non-hospitalized COVID-19	Mean_temporalpole_thickness	Weighted median	19	0.0041	0.0034	0.2174	-0.0024	0.0107
Hospitalized COVID-19 vs. non-hospitalized COVID-19	Mean_temporalpole_thickness	Inverse variance weighted	19	0.0054	0.0028	0.0500	0.0000	0.0109
Hospitalized COVID-19 vs. non-hospitalized COVID-19	Mean_transversetemporal_surface_area	MR Egger	19	1.3422	1.0559	0.2208	-0.7274	3.4119
Hospitalized COVID-19 vs. non-hospitalized COVID-19	Mean_transversetemporal_surface_area	Weighted median	19	-1.1594	0.6410	0.0705	-2.4157	0.0969
Hospitalized COVID-19 vs. non-hospitalized COVID-19	Mean_transversetemporal_surface_area	Inverse variance weighted	19	-1.4242	0.4708	0.0025	-2.3469	-0.5015
Hospitalized COVID-19 vs. non-hospitalized COVID-19	Mean_transversetemporal_thickness	MR Egger	19	-0.0084	0.0037	0.0368	-0.0157	-0.0011
Hospitalized COVID-19 vs. non-hospitalized COVID-19	Mean_transversetemporal_thickness	Weighted median	19	0.0026	0.0025	0.2944	-0.0023	0.0075

continued:

COVID-19 phenotype	Brain structure	Method	nsnp	β	se	p value	β_low95%CI	β_up95%CI
Hospitalized COVID-19 vs. non-hospitalized COVID-19	Mean_transversetemporal_thickness	Inverse variance weighted	19	0.0031	0.0018	0.0849	-0.0004	0.0067
Hospitalized COVID-19 vs. non-hospitalized COVID-19	Mean_Amygdala_volume	MR Egger	19	-8.4282	8.3108	0.3247	-24.7173	7.8609
Hospitalized COVID-19 vs. non-hospitalized COVID-19	Mean_Amygdala_volume	Weighted median	19	-3.4796	4.2083	0.4083	-11.7278	4.7686
Hospitalized COVID-19 vs. non-hospitalized COVID-19	Mean_Amygdala_volume	Inverse variance weighted	19	-0.6433	3.6536	0.8602	-7.8043	6.5177
Hospitalized COVID-19 vs. non-hospitalized COVID-19	Mean_Caudate_volume	MR Egger	19	3.4432	13.4452	0.8010	-22.9094	29.7958
Hospitalized COVID-19 vs. non-hospitalized COVID-19	Mean_Caudate_volume	Weighted median	19	10.8970	8.1527	0.1813	-5.0822	26.8763
Hospitalized COVID-19 vs. non-hospitalized COVID-19	Mean_Caudate_volume	Inverse variance weighted	19	8.3317	5.9831	0.1638	-3.3951	20.0585
Hospitalized COVID-19 vs. non-hospitalized COVID-19	Mean_Hippocampus_volume	MR Egger	19	-12.3921	14.0789	0.3910	-39.9867	15.2025
Hospitalized COVID-19 vs. non-hospitalized COVID-19	Mean_Hippocampus_volume	Weighted median	19	-3.6966	8.3263	0.6571	-20.0161	12.6228
Hospitalized COVID-19 vs. non-hospitalized COVID-19	Mean_Hippocampus_volume	Inverse variance weighted	19	1.3301	6.2649	0.8319	-10.9491	13.6093
Hospitalized COVID-19 vs. non-hospitalized COVID-19	Mean_Intracranial_volume	MR Egger	19	837.2817	4987.5243	0.8687	-8938.2660	10612.8293
Hospitalized COVID-19 vs. non-hospitalized COVID-19	Mean_Intracranial_volume	Weighted median	19	-2917.4251	3261.0512	0.3710	-9309.0855	3474.2353
Hospitalized COVID-19 vs. non-hospitalized COVID-19	Mean_Intracranial_volume	Inverse variance weighted	19	-1839.4095	2288.5751	0.4215	-6325.0166	2646.1977
Hospitalized COVID-19 vs. non-hospitalized COVID-19	Mean_Nucleus_accumbens_volume	MR Egger	19	-4.6806	3.0540	0.1438	-10.6665	1.3054
Hospitalized COVID-19 vs. non-hospitalized COVID-19	Mean_Nucleus_accumbens_volume	Weighted median	19	-0.1425	1.8357	0.9381	-3.7405	3.4555
Hospitalized COVID-19 vs. non-hospitalized COVID-19	Mean_Nucleus_accumbens_volume	Inverse variance weighted	19	-0.5870	1.3521	0.6642	-3.2370	2.0630
Hospitalized COVID-19 vs. non-hospitalized COVID-19	Mean_Pallidum_volume	MR Egger	18	2.2639	5.3620	0.6785	-8.2457	12.7734
Hospitalized COVID-19 vs. non-hospitalized COVID-19	Mean_Pallidum_volume	Weighted median	18	-0.0226	3.1651	0.9943	-6.2261	6.1810
Hospitalized COVID-19 vs. non-hospitalized COVID-19	Mean_Pallidum_volume	Inverse variance weighted	18	-0.0122	2.3638	0.9959	-4.6453	4.6209
Hospitalized COVID-19 vs. non-hospitalized COVID-19	Mean_Putamen_volume	MR Egger	18	-26.5331	16.8095	0.1340	-59.4798	6.4136
Hospitalized COVID-19 vs. non-hospitalized COVID-19	Mean_Putamen_volume	Weighted median	18	3.9669	10.5586	0.7071	-16.7280	24.6617
Hospitalized COVID-19 vs. non-hospitalized COVID-19	Mean_Putamen_volume	Inverse variance weighted	18	2.3941	7.4889	0.7492	-12.2841	17.0723
Hospitalized COVID-19 vs. non-hospitalized COVID-19	Mean_Thalamus_volume	MR Egger	19	-3.3598	17.1566	0.8471	-36.9867	30.2672
Hospitalized COVID-19 vs. non-hospitalized COVID-19	Mean_Thalamus_volume	Weighted median	19	-1.1425	10.6180	0.9143	-21.9538	19.6688
Hospitalized COVID-19 vs. non-hospitalized COVID-19	Mean_Thalamus_volume	Inverse variance weighted	19	2.4317	7.6416	0.7503	-12.5457	17.4092

Abbreviations: COVID-19, coronavirus disease 2019; CI, confidence interval; nSNPs, number of single-nucleotide polymorphisms; se, standard error

Table S5. MR analysis of the causal relationship between severe COVID-19 and brain structure

COVID-19 phenotype	Brain structure	Method	nsnp	β	se	p value	β_low95%CI	β_up95%CI
Severe COVID-19 vs. general population	Mean_bankssts_surface_area	MR Egger	35	1.8657	3.4955	0.5971	-4.9855	8.7169
Severe COVID-19 vs. general population	Mean_bankssts_surface_area	Inverse variance weighted	35	0.4263	1.0439	0.6830	-1.6197	2.4723
Severe COVID-19 vs. general population	Mean_bankssts_surface_area	Weighted median	35	0.2688	1.5712	0.8642	-2.8109	3.3484
Severe COVID-19 vs. general population	Mean_bankssts_thickness	MR Egger	35	-0.0058	0.0038	0.1355	-0.0132	0.0016
Severe COVID-19 vs. general population	Mean_bankssts_thickness	Weighted median	35	-0.0009	0.0016	0.5846	-0.0040	0.0023
Severe COVID-19 vs. general population	Mean_bankssts_thickness	Inverse variance weighted	35	-0.0003	0.0011	0.8257	-0.0025	0.0020
Severe COVID-19 vs. general population	Mean_caudalanteriorcingulate_surface_area	MR Egger	35	1.2328	3.0436	0.6881	-4.7327	7.1983
Severe COVID-19 vs. general population	Mean_caudalanteriorcingulate_surface_area	Weighted median	35	1.3617	1.3189	0.3019	-1.2233	3.9466
Severe COVID-19 vs. general population	Mean_caudalanteriorcingulate_surface_area	Inverse variance weighted	35	1.7611	0.9208	0.0558	-0.0437	3.5658
Severe COVID-19 vs. general population	Mean_caudalanteriorcingulate_thickness	MR Egger	35	0.0005	0.0062	0.9336	-0.0116	0.0126
Severe COVID-19 vs. general population	Mean_caudalanteriorcingulate_thickness	Weighted median	35	0.0016	0.0026	0.5367	-0.0035	0.0068
Severe COVID-19 vs. general population	Mean_caudalanteriorcingulate_thickness	Inverse variance weighted	39	0.0016	0.0019	0.3897	-0.0021	0.0053
Severe COVID-19 vs. general population	Mean_caudalmiddlefrontal_surface_area	MR Egger	35	5.3655	8.0346	0.5089	-10.3824	21.1133
Severe COVID-19 vs. general population	Mean_caudalmiddlefrontal_surface_area	Weighted median	35	-2.0602	3.4111	0.5459	-8.7459	4.6255
Severe COVID-19 vs. general population	Mean_caudalmiddlefrontal_surface_area	Inverse variance weighted	35	-0.4801	2.4293	0.8433	-5.2416	4.2813
Severe COVID-19 vs. general population	Mean_caudalmiddlefrontal_thickness	MR Egger	35	-0.0029	0.0030	0.3403	-0.0087	0.0030
Severe COVID-19 vs. general population	Mean_caudalmiddlefrontal_thickness	Weighted median	35	-0.0004	0.0012	0.7230	-0.0029	0.0020
Severe COVID-19 vs. general population	Mean_caudalmiddlefrontal_thickness	Inverse variance weighted	35	-0.0009	0.0009	0.3363	-0.0026	0.0009
Severe COVID-19 vs. general population	Mean_cuneus_surface_area	MR Egger	35	-8.7890	4.9754	0.0866	-18.5409	0.9628
Severe COVID-19 vs. general population	Mean_cuneus_surface_area	Weighted median	35	-2.2523	2.0424	0.2701	-6.2554	1.7508
Severe COVID-19 vs. general population	Mean_cuneus_surface_area	Inverse variance weighted	35	-1.1320	1.5394	0.4621	-4.1493	1.8853
Severe COVID-19 vs. general population	Mean_cuneus_thickness	MR Egger	39	-0.0002	0.0034	0.9496	-0.0070	0.0065
Severe COVID-19 vs. general population	Mean_cuneus_thickness	Weighted median	39	-0.0013	0.0015	0.3998	-0.0042	0.0017
Severe COVID-19 vs. general population	Mean_cuneus_thickness	Inverse variance weighted	39	-0.0024	0.0010	0.0168	-0.0043	-0.0004
Severe COVID-19 vs. general population	Mean_entorhinal_surface_area	MR Egger	35	-0.0287	2.4327	0.9907	-4.7968	4.7394
Severe COVID-19 vs. general population	Mean_entorhinal_surface_area	Weighted median	35	-0.5283	0.8907	0.5531	-2.2740	1.2174
Severe COVID-19 vs. general population	Mean_entorhinal_surface_area	Inverse variance weighted	35	0.4267	0.7259	0.5567	-0.9962	1.8495
Severe COVID-19 vs. general population	Mean_entorhinal_thickness	MR Egger	35	0.0022	0.0103	0.8301	-0.0180	0.0224
Severe COVID-19 vs. general population	Mean_entorhinal_thickness	Weighted median	35	0.0015	0.0044	0.7308	-0.0072	0.0102
Severe COVID-19 vs. general population	Mean_entorhinal_thickness	Inverse variance weighted	35	0.0000	0.0031	0.9922	-0.0061	0.0060

continued:

COVID-19 phenotype	Brain structure	Method	nsnp	β	se	p value	β_low95%CI	β_up95%CI
Severe COVID-19 vs. general population	Mean_frontalpole_surface_area	MR Egger	35	-0.4600	0.9062	0.6151	-2.2362	1.3163
Severe COVID-19 vs. general population	Mean_frontalpole_surface_area	Weighted median	35	0.1900	0.3848	0.6214	-0.5641	0.9442
Severe COVID-19 vs. general population	Mean_frontalpole_surface_area	Inverse variance weighted	35	-0.1296	0.2741	0.6363	-0.6669	0.4077
Severe COVID-19 vs. general population	Mean_frontalpole_thickness	MR Egger	35	-0.0030	0.0066	0.6513	-0.0161	0.0100
Severe COVID-19 vs. general population	Mean_frontalpole_thickness	Weighted median	35	-0.0012	0.0028	0.6637	-0.0068	0.0043
Severe COVID-19 vs. general population	Mean_frontalpole_thickness	Inverse variance weighted	35	-0.0008	0.0020	0.6857	-0.0048	0.0031
Severe COVID-19 vs. general population	Mean_full_surface_area	MR Egger	5	-1010.4102	1672.1044	0.5883	-4287.7348	2266.9144
Severe COVID-19 vs. general population	Mean_full_surface_area	Weighted median	5	-104.2564	504.4268	0.8363	-1092.9330	884.4202
Severe COVID-19 vs. general population	Mean_full_surface_area	Inverse variance weighted	5	83.7097	470.3527	0.8587	-838.1815	1005.6010
Severe COVID-19 vs. general population	Mean_full_thickness	MR Egger	9	-0.0096	0.0079	0.2651	-0.0251	0.0059
Severe COVID-19 vs. general population	Mean_full_thickness	Weighted median	9	-0.0016	0.0027	0.5458	-0.0068	0.0036
Severe COVID-19 vs. general population	Mean_full_thickness	Inverse variance weighted	9	-0.0041	0.0021	0.0556	-0.0083	0.0001
Severe COVID-19 vs. general population	Mean_fusiform_surface_area	MR Egger	35	-1.1831	8.1132	0.8850	-17.0850	14.7189
Severe COVID-19 vs. general population	Mean_fusiform_surface_area	Weighted median	35	0.6157	3.2792	0.8511	-5.8115	7.0429
Severe COVID-19 vs. general population	Mean_fusiform_surface_area	Inverse variance weighted	35	-1.8179	2.4177	0.4521	-6.5566	2.9208
Severe COVID-19 vs. general population	Mean_fusiform_thickness	MR Egger	35	0.0004	0.0034	0.9024	-0.0063	0.0071
Severe COVID-19 vs. general population	Mean_fusiform_thickness	Weighted median	35	0.0008	0.0014	0.5557	-0.0019	0.0036
Severe COVID-19 vs. general population	Mean_fusiform_thickness	Inverse variance weighted	35	0.0012	0.0010	0.2565	-0.0008	0.0032
Severe COVID-19 vs. general population	Mean_inferiorparietal_surface_area	MR Egger	35	-9.3607	14.0130	0.5088	-36.8263	18.1048
Severe COVID-19 vs. general population	Mean_inferiorparietal_surface_area	Weighted median	35	2.3531	5.7594	0.6829	-8.9353	13.6415
Severe COVID-19 vs. general population	Mean_inferiorparietal_surface_area	Inverse variance weighted	35	4.2985	4.2395	0.3106	-4.0110	12.6080
Severe COVID-19 vs. general population	Mean_inferiorparietal_thickness	MR Egger	35	-0.0028	0.0023	0.2443	-0.0074	0.0018
Severe COVID-19 vs. general population	Mean_inferiorparietal_thickness	Weighted median	35	-0.0014	0.0010	0.1525	-0.0034	0.0005
Severe COVID-19 vs. general population	Mean_inferiorparietal_thickness	Inverse variance weighted	35	-0.0010	0.0007	0.1602	-0.0024	0.0004
Severe COVID-19 vs. general population	Mean_inferiortemporal_surface_area	MR Egger	35	-1.6778	8.7230	0.8487	-18.7748	15.4192
Severe COVID-19 vs. general population	Mean_inferiortemporal_surface_area	Weighted median	35	-2.6666	3.6540	0.4655	-9.8285	4.4953
Severe COVID-19 vs. general population	Mean_inferiortemporal_surface_area	Inverse variance weighted	35	0.4149	2.6266	0.8745	-4.7333	5.5630
Severe COVID-19 vs. general population	Mean_inferiortemporal_thickness	MR Egger	35	0.0020	0.0038	0.5962	-0.0054	0.0095
Severe COVID-19 vs. general population	Mean_inferiortemporal_thickness	Weighted median	35	0.0028	0.0016	0.0767	-0.0003	0.0059
Severe COVID-19 vs. general population	Mean_inferiortemporal_thickness	Inverse variance weighted	35	0.0018	0.0011	0.1211	-0.0005	0.0040

continued:

COVID-19 phenotype	Brain structure	Method	nsnp	β	se	p value	β_low95%CI	β_up95%CI
Severe COVID-19 vs. general population	Mean_insula_surface_area	MR Egger	35	0.0670	6.3303	0.9916	-12.3404	12.4744
Severe COVID-19 vs. general population	Mean_insula_surface_area	Weighted median	35	2.3280	2.3553	0.3230	-2.2885	6.9444
Severe COVID-19 vs. general population	Mean_insula_surface_area	Inverse variance weighted	35	1.2425	1.8903	0.5110	-2.4625	4.9475
Severe COVID-19 vs. general population	Mean_insula_thickness	MR Egger	35	-0.0026	0.0038	0.5022	-0.0100	0.0049
Severe COVID-19 vs. general population	Mean_insula_thickness	Weighted median	35	0.0008	0.0017	0.6216	-0.0025	0.0042
Severe COVID-19 vs. general population	Mean_insula_thickness	Inverse variance weighted	35	-0.0006	0.0011	0.6026	-0.0028	0.0016
Severe COVID-19 vs. general population	Mean_isthmuscingulate_surface_area	MR Egger	35	-3.9185	3.7054	0.2980	-11.1810	3.3441
Severe COVID-19 vs. general population	Mean_isthmuscingulate_surface_area	Weighted median	35	0.0311	1.5334	0.9838	-2.9745	3.0366
Severe COVID-19 vs. general population	Mean_isthmuscingulate_surface_area	Inverse variance weighted	35	-0.6767	1.1180	0.5450	-2.8681	1.5146
Severe COVID-19 vs. general population	Mean_isthmuscingulate_thickness	MR Egger	35	-0.0158	0.0066	0.0230	-0.0288	-0.0028
Severe COVID-19 vs. general population	Mean_isthmuscingulate_thickness	Weighted median	35	-0.0018	0.0025	0.4737	-0.0067	0.0031
Severe COVID-19 vs. general population	Mean_isthmuscingulate_thickness	Inverse variance weighted	35	0.0000	0.0022	0.9925	-0.0042	0.0043
Severe COVID-19 vs. general population	Mean_lateraloccipital_surface_area	MR Egger	35	12.1847	12.7670	0.3468	-12.8385	37.2080
Severe COVID-19 vs. general population	Mean_lateraloccipital_surface_area	Weighted median	35	-3.7057	5.2481	0.4801	-13.9919	6.5805
Severe COVID-19 vs. general population	Mean_lateraloccipital_surface_area	Inverse variance weighted	35	-3.9981	3.9065	0.3061	-11.6548	3.6587
Severe COVID-19 vs. general population	Mean_lateraloccipital_thickness	MR Egger	35	0.0002	0.0031	0.9591	-0.0059	0.0062
Severe COVID-19 vs. general population	Mean_lateraloccipital_thickness	Weighted median	35	-0.0008	0.0013	0.5526	-0.0033	0.0017
Severe COVID-19 vs. general population	Mean_lateraloccipital_thickness	Inverse variance weighted	35	-0.0003	0.0009	0.7612	-0.0021	0.0015
Severe COVID-19 vs. general population	Mean_lateralorbitofrontal_surface_area	MR Egger	35	9.7312	6.8606	0.1654	-3.7155	23.1779
Severe COVID-19 vs. general population	Mean_lateralorbitofrontal_surface_area	Weighted median	35	0.2329	2.5701	0.9278	-4.8046	5.2703
Severe COVID-19 vs. general population	Mean_lateralorbitofrontal_surface_area	Inverse variance weighted	35	2.1463	2.0866	0.3037	-1.9435	6.2361
Severe COVID-19 vs. general population	Mean_lateralorbitofrontal_thickness	MR Egger	35	-0.0008	0.0037	0.8232	-0.0080	0.0064
Severe COVID-19 vs. general population	Mean_lateralorbitofrontal_thickness	Weighted median	35	0.0027	0.0015	0.0773	-0.0003	0.0057
Severe COVID-19 vs. general population	Mean_lateralorbitofrontal_thickness	Inverse variance weighted	35	0.0013	0.0011	0.2223	-0.0008	0.0035
Severe COVID-19 vs. general population	Mean_lingual_surface_area	MR Egger	35	-16.5401	12.7373	0.2031	-41.5053	8.4250
Severe COVID-19 vs. general population	Mean_lingual_surface_area	Weighted median	35	-5.6555	4.3964	0.1983	-14.2724	2.9615
Severe COVID-19 vs. general population	Mean_lingual_surface_area	Inverse variance weighted	35	-3.0411	3.8673	0.4317	-10.6211	4.5389
Severe COVID-19 vs. general population	Mean_lingual_thickness	MR Egger	35	-0.0014	0.0033	0.6758	-0.0080	0.0051
Severe COVID-19 vs. general population	Mean_lingual_thickness	Weighted median	35	0.0000	0.0013	0.9723	-0.0025	0.0026
Severe COVID-19 vs. general population	Mean_lingual_thickness	Inverse variance weighted	35	-0.0011	0.0010	0.2870	-0.0030	0.0009

continued:

COVID-19 phenotype	Brain structure	Method	nsnp	β	se	p value	β_low95%CI	β_up95%CI
Severe COVID-19 vs. general population	Mean_medialorbitofrontal_surface_area	MR Egger	35	-0.0644	4.1619	0.9877	-8.2217	8.0929
Severe COVID-19 vs. general population	Mean_medialorbitofrontal_surface_area	Weighted median	35	0.5483	1.8105	0.7620	-3.0002	4.0968
Severe COVID-19 vs. general population	Mean_medialorbitofrontal_surface_area	Inverse variance weighted	35	1.3899	1.2596	0.2698	-1.0789	3.8588
Severe COVID-19 vs. general population	Mean_medialorbitofrontal_thickness	MR Egger	35	-0.0027	0.0041	0.5045	-0.0107	0.0052
Severe COVID-19 vs. general population	Mean_medialorbitofrontal_thickness	Weighted median	35	0.0015	0.0017	0.3815	-0.0019	0.0049
Severe COVID-19 vs. general population	Mean_medialorbitofrontal_thickness	Inverse variance weighted	35	0.0001	0.0012	0.9120	-0.0023	0.0025
Severe COVID-19 vs. general population	Mean_middletemporal_surface_area	MR Egger	35	0.1106	7.4759	0.9883	-14.5422	14.7634
Severe COVID-19 vs. general population	Mean_middletemporal_surface_area	Weighted median	35	-0.1606	3.2577	0.9607	-6.5458	6.2246
Severe COVID-19 vs. general population	Mean_middletemporal_surface_area	Inverse variance weighted	35	1.4087	2.2626	0.5336	-3.0261	5.8435
Severe COVID-19 vs. general population	Mean_middletemporal_thickness	MR Egger	35	-0.0048	0.0034	0.1698	-0.0114	0.0019
Severe COVID-19 vs. general population	Mean_middletemporal_thickness	Weighted median	35	-0.0008	0.0014	0.5965	-0.0036	0.0021
Severe COVID-19 vs. general population	Mean_middletemporal_thickness	Inverse variance weighted	35	-0.0002	0.0010	0.8696	-0.0022	0.0019
Severe COVID-19 vs. general population	Mean_paracentral_surface_area	MR Egger	35	2.7839	4.5355	0.5436	-6.1057	11.6735
Severe COVID-19 vs. general population	Mean_paracentral_surface_area	Weighted median	35	-0.0337	1.8593	0.9855	-3.6780	3.6105
Severe COVID-19 vs. general population	Mean_paracentral_surface_area	Inverse variance weighted	35	-0.1147	1.3706	0.9333	-2.8011	2.5716
Severe COVID-19 vs. general population	Mean_paracentral_thickness	MR Egger	35	0.0038	0.0034	0.2740	-0.0029	0.0104
Severe COVID-19 vs. general population	Mean_paracentral_thickness	Weighted median	35	0.0012	0.0014	0.3921	-0.0016	0.0040
Severe COVID-19 vs. general population	Mean_paracentral_thickness	Inverse variance weighted	35	0.0007	0.0010	0.4964	-0.0013	0.0027
Severe COVID-19 vs. general population	Mean_parahippocampal_surface_area	MR Egger	35	-4.8443	2.4236	0.0539	-9.5946	-0.0940
Severe COVID-19 vs. general population	Mean_parahippocampal_surface_area	Weighted median	35	0.7114	1.0646	0.5040	-1.3753	2.7981
Severe COVID-19 vs. general population	Mean_parahippocampal_surface_area	Inverse variance weighted	35	0.7025	0.7822	0.3692	-0.8307	2.2357
Severe COVID-19 vs. general population	Mean_parahippocampal_thickness	MR Egger	35	-0.0006	0.0083	0.9419	-0.0169	0.0157
Severe COVID-19 vs. general population	Mean_parahippocampal_thickness	Weighted median	35	-0.0008	0.0037	0.8269	-0.0080	0.0064
Severe COVID-19 vs. general population	Mean_parahippocampal_thickness	Inverse variance weighted	35	-0.0024	0.0025	0.3392	-0.0073	0.0025
Severe COVID-19 vs. general population	Mean_parsopercularis_surface_area	MR Egger	35	7.9274	6.8976	0.2587	-5.5919	21.4467
Severe COVID-19 vs. general population	Mean_parsopercularis_surface_area	Weighted median	35	1.4398	2.3892	0.5467	-3.2430	6.1227
Severe COVID-19 vs. general population	Mean_parsopercularis_surface_area	Inverse variance weighted	35	0.7847	2.0917	0.7075	-3.3150	4.8844
Severe COVID-19 vs. general population	Mean_parsopercularis_thickness	MR Egger	35	0.0030	0.0033	0.3662	-0.0035	0.0095
Severe COVID-19 vs. general population	Mean_parsopercularis_thickness	Weighted median	35	-0.0009	0.0012	0.4794	-0.0033	0.0016
Severe COVID-19 vs. general population	Mean_parsopercularis_thickness	Inverse variance weighted	35	-0.0002	0.0010	0.8629	-0.0021	0.0018

continued:

COVID-19 phenotype	Brain structure	Method	nsnp	β	se	p value	β_low95%CI	β_up95%CI
Severe COVID-19 vs. general population	Mean_parsorbitalis_surface_area	MR Egger	35	2.3806	1.9045	0.2201	-1.3521	6.1134
Severe COVID-19 vs. general population	Mean_parsorbitalis_surface_area	Weighted median	35	-0.7487	0.8083	0.3543	-2.3329	0.8355
Severe COVID-19 vs. general population	Mean_parsorbitalis_surface_area	Inverse variance weighted	35	-0.4154	0.5875	0.4795	-1.5669	0.7360
Severe COVID-19 vs. general population	Mean_parsorbitalis_thickness	MR Egger	35	-0.0012	0.0046	0.7933	-0.0101	0.0077
Severe COVID-19 vs. general population	Mean_parsorbitalis_thickness	Weighted median	35	0.0029	0.0020	0.1323	-0.0009	0.0068
Severe COVID-19 vs. general population	Mean_parsorbitalis_thickness	Inverse variance weighted	35	0.0029	0.0014	0.0329	0.0002	0.0056
Severe COVID-19 vs. general population	Mean_parstriangularis_surface_area	MR Egger	35	-9.8810	4.9797	0.0556	-19.6412	-0.1207
Severe COVID-19 vs. general population	Mean_parstriangularis_surface_area	Weighted median	35	0.1880	2.1130	0.9291	-3.9535	4.3295
Severe COVID-19 vs. general population	Mean_parstriangularis_surface_area	Inverse variance weighted	35	-0.6730	1.5060	0.6550	-3.6248	2.2788
Severe COVID-19 vs. general population	Mean_parstriangularis_thickness	MR Egger	35	-0.0021	0.0031	0.5131	-0.0082	0.0041
Severe COVID-19 vs. general population	Mean_parstriangularis_thickness	Weighted median	35	0.0012	0.0013	0.3548	-0.0014	0.0038
Severe COVID-19 vs. general population	Mean_parstriangularis_thickness	Inverse variance weighted	35	0.0003	0.0009	0.7250	-0.0015	0.0022
Severe COVID-19 vs. general population	Mean_pericalcarine_surface_area	MR Egger	35	-10.9645	6.7022	0.1114	-24.1009	2.1718
Severe COVID-19 vs. general population	Mean_pericalcarine_surface_area	Weighted median	35	-1.6265	2.7418	0.5530	-7.0005	3.7475
Severe COVID-19 vs. general population	Mean_pericalcarine_surface_area	Inverse variance weighted	35	-1.9428	2.0568	0.3449	-5.9742	2.0885
Severe COVID-19 vs. general population	Mean_pericalcarine_thickness	MR Egger	35	0.0007	0.0032	0.8319	-0.0056	0.0070
Severe COVID-19 vs. general population	Mean_pericalcarine_thickness	Weighted median	35	-0.0027	0.0014	0.0466	-0.0054	0.0000
Severe COVID-19 vs. general population	Mean_pericalcarine_thickness	Inverse variance weighted	35	-0.0019	0.0010	0.0512	-0.0038	0.0000
Severe COVID-19 vs. general population	Mean_postcentral_surface_area	MR Egger	35	8.5167	9.8441	0.3932	-10.7779	27.8112
Severe COVID-19 vs. general population	Mean_postcentral_surface_area	Weighted median	35	6.2445	3.9772	0.1164	-1.5507	14.0398
Severe COVID-19 vs. general population	Mean_postcentral_surface_area	Inverse variance weighted	35	3.3504	2.9472	0.2556	-2.4261	9.1268
Severe COVID-19 vs. general population	Mean_postcentral_thickness	MR Egger	35	0.0013	0.0030	0.6749	-0.0046	0.0072
Severe COVID-19 vs. general population	Mean_postcentral_thickness	Weighted median	35	0.0000	0.0012	1.0000	-0.0023	0.0023
Severe COVID-19 vs. general population	Mean_postcentral_thickness	Inverse variance weighted	35	0.0000	0.0009	0.9990	-0.0018	0.0018
Severe COVID-19 vs. general population	Mean_posteriorcingulate_surface_area	MR Egger	35	2.7663	3.6511	0.4540	-4.3898	9.9224
Severe COVID-19 vs. general population	Mean_posteriorcingulate_surface_area	Weighted median	35	3.0710	1.5582	0.0487	0.0168	6.1251
Severe COVID-19 vs. general population	Mean_posteriorcingulate_surface_area	Inverse variance weighted	35	1.7789	1.0889	0.1024	-0.3555	3.9132
Severe COVID-19 vs. general population	Mean_posteriorcingulate_thickness	MR Egger	35	-0.0012	0.0040	0.7660	-0.0091	0.0067
Severe COVID-19 vs. general population	Mean_posteriorcingulate_thickness	Weighted median	35	0.0012	0.0016	0.4570	-0.0020	0.0044
Severe COVID-19 vs. general population	Mean_posteriorcingulate_thickness	Inverse variance weighted	35	0.0018	0.0012	0.1382	-0.0006	0.0042

continued:

COVID-19 phenotype	Brain structure	Method	nsnp	β	se	p value	β_low95%CI	β_up95%CI
Severe COVID-19 vs. general population	Mean_precentral_surface_area	MR Egger	35	-7.5193	11.6768	0.5241	-30.4059	15.3673
Severe COVID-19 vs. general population	Mean_precentral_surface_area	Weighted median	35	-5.0681	4.7799	0.2890	-14.4366	4.3004
Severe COVID-19 vs. general population	Mean_precentral_surface_area	Inverse variance weighted	35	0.8955	3.5110	0.7987	-5.9860	7.7769
Severe COVID-19 vs. general population	Mean_precentral_thickness	MR Egger	35	0.0035	0.0029	0.2348	-0.0022	0.0092
Severe COVID-19 vs. general population	Mean_precentral_thickness	Weighted median	35	-0.0008	0.0012	0.4878	-0.0032	0.0015
Severe COVID-19 vs. general population	Mean_precentral_thickness	Inverse variance weighted	35	-0.0006	0.0009	0.4798	-0.0024	0.0011
Severe COVID-19 vs. general population	Mean_precuneus_surface_area	MR Egger	35	4.1681	8.7128	0.6355	-12.9090	21.2453
Severe COVID-19 vs. general population	Mean_precuneus_surface_area	Weighted median	35	-4.8312	3.6865	0.1900	-12.0568	2.3944
Severe COVID-19 vs. general population	Mean_precuneus_surface_area	Inverse variance weighted	35	-3.2275	2.6362	0.2208	-8.3945	1.9395
Severe COVID-19 vs. general population	Mean_precuneus_thickness	MR Egger	35	0.0036	0.0027	0.1937	-0.0017	0.0089
Severe COVID-19 vs. general population	Mean_precuneus_thickness	Weighted median	35	-0.0005	0.0011	0.6322	-0.0026	0.0016
Severe COVID-19 vs. general population	Mean_precuneus_thickness	Inverse variance weighted	35	-0.0007	0.0008	0.3984	-0.0024	0.0009
Severe COVID-19 vs. general population	Mean_rostralanteriorcingulate_surface_area	MR Egger	35	-0.0287	2.9500	0.9923	-5.8108	5.7533
Severe COVID-19 vs. general population	Mean_rostralanteriorcingulate_surface_area	Weighted median	35	1.7853	1.2532	0.1543	-0.6709	4.2416
Severe COVID-19 vs. general population	Mean_rostralanteriorcingulate_surface_area	Inverse variance weighted	35	1.9634	0.8933	0.0280	0.2126	3.7142
Severe COVID-19 vs. general population	Mean_rostralanteriorcingulate_thickness	MR Egger	35	0.0078	0.0055	0.1635	-0.0029	0.0186
Severe COVID-19 vs. general population	Mean_rostralanteriorcingulate_thickness	Weighted median	35	0.0000	0.0023	0.9910	-0.0044	0.0045
Severe COVID-19 vs. general population	Mean_rostralanteriorcingulate_thickness	Inverse variance weighted	35	-0.0005	0.0017	0.7696	-0.0038	0.0028
Severe COVID-19 vs. general population	Mean_rostralmiddlefrontal_surface_area	MR Egger	35	-12.8470	13.2598	0.3397	-38.8362	13.1422
Severe COVID-19 vs. general population	Mean_rostralmiddlefrontal_surface_area	Weighted median	35	-4.8492	5.5217	0.3798	-15.6717	5.9734
Severe COVID-19 vs. general population	Mean_rostralmiddlefrontal_surface_area	Inverse variance weighted	35	-3.9685	3.9813	0.3189	-11.7719	3.8350
Severe COVID-19 vs. general population	Mean_rostralmiddlefrontal_thickness	MR Egger	35	-0.0038	0.0028	0.1904	-0.0093	0.0018
Severe COVID-19 vs. general population	Mean_rostralmiddlefrontal_thickness	Weighted median	35	0.0000	0.0012	1.0000	-0.0023	0.0023
Severe COVID-19 vs. general population	Mean_rostralmiddlefrontal_thickness	Inverse variance weighted	35	0.0002	0.0009	0.8252	-0.0015	0.0019
Severe COVID-19 vs. general population	Mean_superiorfrontal_surface_area	MR Egger	35	-10.0125	13.0178	0.4473	-35.5275	15.5024
Severe COVID-19 vs. general population	Mean_superiorfrontal_surface_area	Weighted median	35	3.3652	5.1326	0.5120	-6.6947	13.4251
Severe COVID-19 vs. general population	Mean_superiorfrontal_surface_area	Inverse variance weighted	35	-0.2359	3.9168	0.9520	-7.9128	7.4410
Severe COVID-19 vs. general population	Mean_superiorfrontal_thickness	MR Egger	35	-0.0044	0.0031	0.1677	-0.0106	0.0017
Severe COVID-19 vs. general population	Mean_superiorfrontal_thickness	Weighted median	35	-0.0022	0.0012	0.0586	-0.0045	0.0001
Severe COVID-19 vs. general population	Mean_superiorfrontal_thickness	Inverse variance weighted	35	-0.0017	0.0009	0.0750	-0.0035	0.0002

continued:

COVID-19 phenotype	Brain structure	Method	nsnp	β	se	p value	β_low95%CI	β_up95%CI
Severe COVID-19 vs. general population	Mean_superiorparietal_surface_area	MR Egger	35	6.4846	12.4687	0.6065	-17.9541	30.9233
Severe COVID-19 vs. general population	Mean_superiorparietal_surface_area	Weighted median	35	-0.7262	5.0312	0.8852	-10.5873	9.1348
Severe COVID-19 vs. general population	Mean_superiorparietal_surface_area	Inverse variance weighted	35	-2.8691	3.7705	0.4467	-10.2593	4.5212
Severe COVID-19 vs. general population	Mean_superiorparietal_thickness	MR Egger	35	0.0043	0.0027	0.1207	-0.0010	0.0096
Severe COVID-19 vs. general population	Mean_superiorparietal_thickness	Weighted median	35	-0.0010	0.0011	0.3673	-0.0032	0.0012
Severe COVID-19 vs. general population	Mean_superiorparietal_thickness	Inverse variance weighted	35	-0.0011	0.0009	0.2100	-0.0028	0.0006
Severe COVID-19 vs. general population	Mean_superiortemporal_surface_area	MR Egger	35	17.3036	7.7187	0.0318	2.1750	32.4322
Severe COVID-19 vs. general population	Mean_superiortemporal_surface_area	Weighted median	35	3.8152	3.1462	0.2253	-2.3513	9.9817
Severe COVID-19 vs. general population	Mean_superiortemporal_surface_area	Inverse variance weighted	35	1.8476	2.4481	0.4504	-2.9507	6.6460
Severe COVID-19 vs. general population	Mean_superiortemporal_thickness	MR Egger	35	0.0044	0.0033	0.1977	-0.0022	0.0109
Severe COVID-19 vs. general population	Mean_superiortemporal_thickness	Weighted median	35	0.0013	0.0014	0.3377	-0.0014	0.0041
Severe COVID-19 vs. general population	Mean_superiortemporal_thickness	Inverse variance weighted	35	0.0010	0.0010	0.3292	-0.0010	0.0030
Severe COVID-19 vs. general population	Mean_supramarginal_surface_area	MR Egger	35	26.3357	10.8832	0.0212	5.0046	47.6667
Severe COVID-19 vs. general population	Mean_supramarginal_surface_area	Weighted median	35	4.8220	4.4262	0.2760	-3.8533	13.4973
Severe COVID-19 vs. general population	Mean_supramarginal_surface_area	Inverse variance weighted	35	2.1139	3.5018	0.5461	-4.7496	8.9774
Severe COVID-19 vs. general population	Mean_supramarginal_thickness	MR Egger	35	-0.0032	0.0025	0.2029	-0.0080	0.0016
Severe COVID-19 vs. general population	Mean_supramarginal_thickness	Weighted median	35	-0.0008	0.0010	0.4288	-0.0028	0.0012
Severe COVID-19 vs. general population	Mean_supramarginal_thickness	Inverse variance weighted	35	-0.0012	0.0007	0.1040	-0.0027	0.0002
Severe COVID-19 vs. general population	Mean_temporalpole_surface_area	MR Egger	35	-0.1674	1.5164	0.9128	-3.1396	2.8048
Severe COVID-19 vs. general population	Mean_temporalpole_surface_area	Weighted median	35	0.1641	0.6350	0.7960	-1.0804	1.4087
Severe COVID-19 vs. general population	Mean_temporalpole_surface_area	Inverse variance weighted	35	0.0005	0.4526	0.9991	-0.8866	0.8876
Severe COVID-19 vs. general population	Mean_temporalpole_thickness	MR Egger	35	0.0109	0.0083	0.2001	-0.0054	0.0272
Severe COVID-19 vs. general population	Mean_temporalpole_thickness	Weighted median	35	0.0070	0.0036	0.0494	0.0000	0.0140
Severe COVID-19 vs. general population	Mean_temporalpole_thickness	Inverse variance weighted	35	0.0048	0.0025	0.0560	-0.0001	0.0098
Severe COVID-19 vs. general population	Mean_transversetemporal_surface_area	MR Egger	35	0.4448	1.5532	0.7764	-2.5995	3.4890
Severe COVID-19 vs. general population	Mean_transversetemporal_surface_area	Weighted median	35	0.5026	0.6647	0.4496	-0.8003	1.8054
Severe COVID-19 vs. general population	Mean_transversetemporal_surface_area	Inverse variance weighted	35	0.4973	0.4698	0.2898	-0.4234	1.4181
Severe COVID-19 vs. general population	Mean_transversetemporal_thickness	MR Egger	35	0.0083	0.0064	0.2004	-0.0042	0.0208
Severe COVID-19 vs. general population	Mean_transversetemporal_thickness	Weighted median	35	0.0004	0.0026	0.8887	-0.0047	0.0055
Severe COVID-19 vs. general population	Mean_transversetemporal_thickness	Inverse variance weighted	35	-0.0002	0.0020	0.9157	-0.0040	0.0036

continued:

COVID-19 phenotype	Brain structure	Method	nsnp	β	se	p value	β_low95%CI	β_up95%CI
Severe COVID-19 vs. general population	Mean_Amygdala_volume	MR Egger	36	11.7901	6.7981	0.0919	-1.5341	25.1143
Severe COVID-19 vs. general population	Mean_Amygdala_volume	Weighted median	36	4.3316	3.9030	0.2671	-3.3183	11.9815
Severe COVID-19 vs. general population	Mean_Amygdala_volume	Inverse variance weighted	36	1.8452	2.6353	0.4838	-3.3199	7.0104
Severe COVID-19 vs. general population	Mean_Caudate_volume	MR Egger	36	0.0414	16.3281	0.9980	-31.9616	32.0445
Severe COVID-19 vs. general population	Mean_Caudate_volume	Weighted median	36	10.4333	8.3626	0.2122	-5.9573	26.8240
Severe COVID-19 vs. general population	Mean_Caudate_volume	Inverse variance weighted	36	7.5096	6.2672	0.2308	-4.7742	19.7933
Severe COVID-19 vs. general population	Mean_Hippocampus_volume	MR Egger	36	-1.0955	13.5433	0.9360	-27.6403	25.4493
Severe COVID-19 vs. general population	Mean_Hippocampus_volume	Weighted median	36	-15.8282	7.8174	0.0429	-31.1503	-0.5062
Severe COVID-19 vs. general population	Mean_Hippocampus_volume	Inverse variance weighted	36	-15.9127	5.2520	0.0024	-26.2067	-5.6188
Severe COVID-19 vs. general population	Mean_Intracranial_volume	MR Egger	37	-491.5907	5421.0553	0.9283	-11116.8592	10133.6778
Severe COVID-19 vs. general population	Mean_Intracranial_volume	Weighted median	37	-5645.3364	3128.7042	0.0712	-11777.5965	486.9238
Severe COVID-19 vs. general population	Mean_Intracranial_volume	Inverse variance weighted	37	-4078.2338	2130.6894	0.0556	-8254.3850	97.9173
Severe COVID-19 vs. general population	Mean_Nucleus_accumbens_volume	MR Egger	36	-0.0164	3.1668	0.9959	-6.2233	6.1905
Severe COVID-19 vs. general population	Mean_Nucleus_accumbens_volume	Weighted median	36	-0.4427	1.8913	0.8150	-4.1497	3.2644
Severe COVID-19 vs. general population	Mean_Nucleus_accumbens_volume	Inverse variance weighted	36	-1.1716	1.2286	0.3403	-3.5797	1.2365
Severe COVID-19 vs. general population	Mean_Pallidum_volume	MR Egger	36	0.2068	5.5201	0.9703	-10.6126	11.0262
Severe COVID-19 vs. general population	Mean_Pallidum_volume	Weighted median	36	0.4992	3.3739	0.8824	-6.1136	7.1120
Severe COVID-19 vs. general population	Mean_Pallidum_volume	Inverse variance weighted	36	0.9168	2.1381	0.6681	-3.2737	5.1074
Severe COVID-19 vs. general population	Mean_Putamen_volume	MR Egger	36	10.8351	17.2271	0.5336	-22.9301	44.6003
Severe COVID-19 vs. general population	Mean_Putamen_volume	Weighted median	36	-1.9250	9.7458	0.8434	-21.0267	17.1767
Severe COVID-19 vs. general population	Mean_Putamen_volume	Inverse variance weighted	36	-5.5525	6.6832	0.4061	-18.6516	7.5466
Severe COVID-19 vs. general population	Mean_Thalamus_volume	MR Egger	36	-6.1526	18.0255	0.7350	-41.4826	29.1773
Severe COVID-19 vs. general population	Mean_Thalamus_volume	Weighted median	36	-0.5059	10.3840	0.9611	-20.8585	19.8468
Severe COVID-19 vs. general population	Mean_Thalamus_volume	Inverse variance weighted	36	-5.8383	6.9904	0.4036	-19.5395	7.8629

Abbreviations: COVID-19, coronavirus disease 2019; CI, confidence interval; nSNPs, number of single-nucleotide polymorphisms; se, standard error

Table S6. MR analysis of the causal relationship between severe COVID-19 with respiratory failure and brain structure

COVID-19 phenotype	Brain structure	Method	nsnp	β	se	p value	β_low95%CI	β_up95%CI
Severe COVID-19 with respiratory failure vs. general population	Mean_bankssts_surface_area	MR Egger	14	3.9406	2.0421	0.0776	-0.0619	7.9431
Severe COVID-19 with respiratory failure vs. general population	Mean_bankssts_surface_area	Weighted median	14	1.0223	1.0750	0.3416	-1.0847	3.1292
Severe COVID-19 with respiratory failure vs. general population	Mean_bankssts_surface_area	Inverse variance weighted	14	0.1563	0.9311	0.8667	-1.6687	1.9813
Severe COVID-19 with respiratory failure vs. general population	Mean_bankssts_thickness	MR Egger	14	-0.0021	0.0027	0.4442	-0.0073	0.0031
Severe COVID-19 with respiratory failure vs. general population	Mean_bankssts_thickness	Weighted median	14	-0.0001	0.0012	0.9074	-0.0025	0.0022
Severe COVID-19 with respiratory failure vs. general population	Mean_bankssts_thickness	Inverse variance weighted	14	0.0010	0.0011	0.3845	-0.0012	0.0032
Severe COVID-19 with respiratory failure vs. general population	Mean_caudalanteriorcingulate_surface_area	MR Egger	14	-0.2476	1.6637	0.8841	-3.5084	3.0131
Severe COVID-19 with respiratory failure vs. general population	Mean_caudalanteriorcingulate_surface_area	Weighted median	14	0.6633	0.9342	0.4777	-1.1678	2.4944
Severe COVID-19 with respiratory failure vs. general population	Mean_caudalanteriorcingulate_surface_area	Inverse variance weighted	14	0.3394	0.6731	0.6141	-0.9799	1.6586
Severe COVID-19 with respiratory failure vs. general population	Mean_caudalanteriorcingulate_thickness	MR Egger	14	-0.0013	0.0034	0.6995	-0.0080	0.0053
Severe COVID-19 with respiratory failure vs. general population	Mean_caudalanteriorcingulate_thickness	Weighted median	14	-0.0021	0.0018	0.2329	-0.0057	0.0014
Severe COVID-19 with respiratory failure vs. general population	Mean_caudalanteriorcingulate_thickness	Inverse variance weighted	14	-0.0023	0.0014	0.0920	-0.0050	0.0004
Severe COVID-19 with respiratory failure vs. general population	Mean_caudalmiddlefrontal_surface_area	MR Egger	14	-0.1992	5.5113	0.9718	-11.0013	10.6030
Severe COVID-19 with respiratory failure vs. general population	Mean_caudalmiddlefrontal_surface_area	Weighted median	14	-3.7399	2.7288	0.1705	-9.0882	1.6085
Severe COVID-19 with respiratory failure vs. general population	Mean_caudalmiddlefrontal_surface_area	Inverse variance weighted	14	-0.8598	2.1696	0.6919	-5.1123	3.3926
Severe COVID-19 with respiratory failure vs. general population	Mean_caudalmiddlefrontal_thickness	MR Egger	14	-0.0007	0.0019	0.7077	-0.0044	0.0030
Severe COVID-19 with respiratory failure vs. general population	Mean_caudalmiddlefrontal_thickness	Weighted median	14	-0.0015	0.0009	0.1154	-0.0033	0.0004
Severe COVID-19 with respiratory failure vs. general population	Mean_caudalmiddlefrontal_thickness	Inverse variance weighted	14	-0.0017	0.0008	0.0244	-0.0032	-0.0002
Severe COVID-19 with respiratory failure vs. general population	Mean_cuneus_surface_area	MR Egger	14	-0.5848	2.4916	0.8184	-5.4683	4.2987
Severe COVID-19 with respiratory failure vs. general population	Mean_cuneus_surface_area	Weighted median	14	-2.0134	1.3480	0.1353	-4.6555	0.6286
Severe COVID-19 with respiratory failure vs. general population	Mean_cuneus_surface_area	Inverse variance weighted	14	-1.7843	1.0202	0.0803	-3.7839	0.2153
Severe COVID-19 with respiratory failure vs. general population	Mean_cuneus_thickness	MR Egger	14	-0.0003	0.0023	0.9084	-0.0048	0.0042
Severe COVID-19 with respiratory failure vs. general population	Mean_cuneus_thickness	Weighted median	14	-0.0006	0.0010	0.5719	-0.0026	0.0014
Severe COVID-19 with respiratory failure vs. general population	Mean_cuneus_thickness	Inverse variance weighted	14	0.0003	0.0009	0.7651	-0.0015	0.0020
Severe COVID-19 with respiratory failure vs. general population	Mean_entorhinal_surface_area	MR Egger	14	-0.2563	1.3070	0.8478	-2.8181	2.3054
Severe COVID-19 with respiratory failure vs. general population	Mean_entorhinal_surface_area	Weighted median	14	0.8029	0.6431	0.2119	-0.4577	2.0634
Severe COVID-19 with respiratory failure vs. general population	Mean_entorhinal_surface_area	Inverse variance weighted	14	0.2635	0.5203	0.6126	-0.7563	1.2833
Severe COVID-19 with respiratory failure vs. general population	Mean_entorhinal_thickness	MR Egger	14	-0.0003	0.0052	0.9612	-0.0105	0.0100
Severe COVID-19 with respiratory failure vs. general population	Mean_entorhinal_thickness	Weighted median	14	0.0006	0.0027	0.8259	-0.0048	0.0060
Severe COVID-19 with respiratory failure vs. general population	Mean_entorhinal_thickness	Inverse variance weighted	14	-0.0004	0.0021	0.8583	-0.0046	0.0038

continued:

COVID-19 phenotype	Brain structure	Method	nsnp	β	se	p value	β_low95%CI	β_up95%CI
Severe COVID-19 with respiratory failure vs. general population	Mean_frontalpole_surface_area	MR Egger	14	0.6074	0.5906	0.3240	-0.5502	1.7651
Severe COVID-19 with respiratory failure vs. general population	Mean_frontalpole_surface_area	Weighted median	14	0.0701	0.2974	0.8137	-0.5128	0.6529
Severe COVID-19 with respiratory failure vs. general population	Mean_frontalpole_surface_area	Inverse variance weighted	14	0.1794	0.2381	0.4512	-0.2873	0.6461
Severe COVID-19 with respiratory failure vs. general population	Mean_frontalpole_thickness	MR Egger	14	-0.0024	0.0036	0.5177	-0.0094	0.0046
Severe COVID-19 with respiratory failure vs. general population	Mean_frontalpole_thickness	Weighted median	14	-0.0010	0.0020	0.6161	-0.0049	0.0029
Severe COVID-19 with respiratory failure vs. general population	Mean_frontalpole_thickness	Inverse variance weighted	14	-0.0015	0.0015	0.3110	-0.0044	0.0014
Severe COVID-19 with respiratory failure vs. general population	Mean_full_surface_area	Inverse variance weighted	2	337.1134	231.4339	0.1452	-116.4971	790.7239
Severe COVID-19 with respiratory failure vs. general population	Mean_full_thickness	MR Egger	4	0.0011	0.0035	0.7794	-0.0058	0.0081
Severe COVID-19 with respiratory failure vs. general population	Mean_full_thickness	Weighted median	4	0.0001	0.0015	0.9413	-0.0028	0.0030
Severe COVID-19 with respiratory failure vs. general population	Mean_full_thickness	Inverse variance weighted	4	0.0010	0.0014	0.4896	-0.0018	0.0037
Severe COVID-19 with respiratory failure vs. general population	Mean_fusiform_surface_area	MR Egger	14	-2.7559	4.1233	0.5165	-10.8375	5.3257
Severe COVID-19 with respiratory failure vs. general population	Mean_fusiform_surface_area	Weighted median	14	1.9040	2.3013	0.4080	-2.6065	6.4145
Severe COVID-19 with respiratory failure vs. general population	Mean_fusiform_surface_area	Inverse variance weighted	14	0.0588	1.6888	0.9722	-3.2513	3.3689
Severe COVID-19 with respiratory failure vs. general population	Mean_fusiform_thickness	MR Egger	14	-0.0010	0.0019	0.6109	-0.0048	0.0028
Severe COVID-19 with respiratory failure vs. general population	Mean_fusiform_thickness	Weighted median	14	-0.0004	0.0010	0.6675	-0.0023	0.0015
Severe COVID-19 with respiratory failure vs. general population	Mean_fusiform_thickness	Inverse variance weighted	14	-0.0008	0.0008	0.2648	-0.0023	0.0006
Severe COVID-19 with respiratory failure vs. general population	Mean_inferiorparietal_surface_area	MR Egger	14	7.0077	7.8679	0.3906	-8.4134	22.4287
Severe COVID-19 with respiratory failure vs. general population	Mean_inferiorparietal_surface_area	Weighted median	14	2.3479	4.0107	0.5583	-5.5131	10.2089
Severe COVID-19 with respiratory failure vs. general population	Mean_inferiorparietal_surface_area	Inverse variance weighted	14	0.2526	3.2096	0.9373	-6.0381	6.5434
Severe COVID-19 with respiratory failure vs. general population	Mean_inferiorparietal_thickness	MR Egger	14	-0.0010	0.0013	0.4555	-0.0034	0.0015
Severe COVID-19 with respiratory failure vs. general population	Mean_inferiorparietal_thickness	Weighted median	14	0.0003	0.0007	0.6239	-0.0010	0.0017
Severe COVID-19 with respiratory failure vs. general population	Mean_inferiorparietal_thickness	Inverse variance weighted	14	-0.0003	0.0005	0.5976	-0.0013	0.0007
Severe COVID-19 with respiratory failure vs. general population	Mean_inferiortemporal_surface_area	MR Egger	14	-4.8056	4.6754	0.3243	-13.9693	4.3582
Severe COVID-19 with respiratory failure vs. general population	Mean_inferiortemporal_surface_area	Weighted median	14	-2.7754	2.6245	0.2903	-7.9194	2.3685
Severe COVID-19 with respiratory failure vs. general population	Mean_inferiortemporal_surface_area	Inverse variance weighted	14	-2.5420	1.9165	0.1847	-6.2982	1.2143
Severe COVID-19 with respiratory failure vs. general population	Mean_inferiortemporal_thickness	MR Egger	14	-0.0046	0.0019	0.0351	-0.0084	-0.0008
Severe COVID-19 with respiratory failure vs. general population	Mean_inferiortemporal_thickness	Weighted median	14	-0.0007	0.0010	0.4826	-0.0028	0.0013
Severe COVID-19 with respiratory failure vs. general population	Mean_inferiortemporal_thickness	Inverse variance weighted	14	-0.0015	0.0008	0.0510	-0.0031	0.0000
Severe COVID-19 with respiratory failure vs. general population	Mean_insula_surface_area	MR Egger	14	2.3517	2.7952	0.4166	-3.1270	7.8304
Severe COVID-19 with respiratory failure vs. general population	Mean_insula_surface_area	Weighted median	14	0.7878	1.5734	0.6166	-2.2961	3.8718

continued:

COVID-19 phenotype	Brain structure	Method	nsnp	β	se	p value	β_low95%CI	β_up95%CI
Severe COVID-19 with respiratory failure vs. general population	Mean_insula_surface_area	Inverse variance weighted	14	0.8180	1.1462	0.4754	-1.4286	3.0646
Severe COVID-19 with respiratory failure vs. general population	Mean_insula_thickness	MR Egger	14	0.0010	0.0029	0.7260	-0.0046	0.0067
Severe COVID-19 with respiratory failure vs. general population	Mean_insula_thickness	Weighted median	14	-0.0018	0.0012	0.1337	-0.0042	0.0006
Severe COVID-19 with respiratory failure vs. general population	Mean_insula_thickness	Inverse variance weighted	14	0.0002	0.0011	0.8730	-0.0020	0.0024
Severe COVID-19 with respiratory failure vs. general population	Mean_isthmuscingulate_surface_area	MR Egger	14	1.2462	1.8893	0.5220	-2.4569	4.9494
Severe COVID-19 with respiratory failure vs. general population	Mean_isthmuscingulate_surface_area	Weighted median	14	1.2540	1.0720	0.2421	-0.8471	3.3551
Severe COVID-19 with respiratory failure vs. general population	Mean_isthmuscingulate_surface_area	Inverse variance weighted	14	0.5822	0.7730	0.4514	-0.9330	2.0973
Severe COVID-19 with respiratory failure vs. general population	Mean_isthmuscingulate_thickness	MR Egger	14	0.0010	0.0032	0.7676	-0.0053	0.0072
Severe COVID-19 with respiratory failure vs. general population	Mean_isthmuscingulate_thickness	Weighted median	14	-0.0025	0.0017	0.1453	-0.0059	0.0009
Severe COVID-19 with respiratory failure vs. general population	Mean_isthmuscingulate_thickness	Inverse variance weighted	14	-0.0016	0.0013	0.2199	-0.0041	0.0009
Severe COVID-19 with respiratory failure vs. general population	Mean_lateraloccipital_surface_area	MR Egger	14	-7.9367	6.1949	0.2243	-20.0786	4.2052
Severe COVID-19 with respiratory failure vs. general population	Mean_lateraloccipital_surface_area	Weighted median	14	0.3536	3.3338	0.9155	-6.1806	6.8877
Severe COVID-19 with respiratory failure vs. general population	Mean_lateraloccipital_surface_area	Inverse variance weighted	14	0.8391	2.5404	0.7412	-4.1401	5.8182
Severe COVID-19 with respiratory failure vs. general population	Mean_lateraloccipital_thickness	MR Egger	14	-0.0005	0.0015	0.7683	-0.0034	0.0025
Severe COVID-19 with respiratory failure vs. general population	Mean_lateraloccipital_thickness	Weighted median	14	-0.0001	0.0008	0.9391	-0.0017	0.0016
Severe COVID-19 with respiratory failure vs. general population	Mean_lateraloccipital_thickness	Inverse variance weighted	14	0.0001	0.0006	0.9108	-0.0011	0.0013
Severe COVID-19 with respiratory failure vs. general population	Mean_lateralorbitofrontal_surface_area	MR Egger	14	1.2957	3.4851	0.7165	-5.5352	8.1266
Severe COVID-19 with respiratory failure vs. general population	Mean_lateralorbitofrontal_surface_area	Weighted median	14	0.5137	1.8011	0.7755	-3.0164	4.0438
Severe COVID-19 with respiratory failure vs. general population	Mean_lateralorbitofrontal_surface_area	Inverse variance weighted	14	-1.4114	1.4149	0.3185	-4.1846	1.3618
Severe COVID-19 with respiratory failure vs. general population	Mean_lateralorbitofrontal_thickness	MR Egger	14	-0.0011	0.0019	0.5702	-0.0049	0.0026
Severe COVID-19 with respiratory failure vs. general population	Mean_lateralorbitofrontal_thickness	Weighted median	14	-0.0001	0.0011	0.9138	-0.0023	0.0020
Severe COVID-19 with respiratory failure vs. general population	Mean_lateralorbitofrontal_thickness	Inverse variance weighted	14	0.0000	0.0008	0.9837	-0.0015	0.0016
Severe COVID-19 with respiratory failure vs. general population	Mean_lingual_surface_area	MR Egger	14	-6.6680	4.9924	0.2065	-16.4531	3.1171
Severe COVID-19 with respiratory failure vs. general population	Mean_lingual_surface_area	Weighted median	14	-2.5377	2.6287	0.3344	-7.6899	2.6145
Severe COVID-19 with respiratory failure vs. general population	Mean_lingual_surface_area	Inverse variance weighted	14	-3.6393	2.0452	0.0752	-7.6478	0.3692
Severe COVID-19 with respiratory failure vs. general population	Mean_lingual_thickness	MR Egger	14	0.0033	0.0016	0.0610	0.0002	0.0064
Severe COVID-19 with respiratory failure vs. general population	Mean_lingual_thickness	Weighted median	14	0.0006	0.0009	0.5313	-0.0012	0.0024
Severe COVID-19 with respiratory failure vs. general population	Mean_lingual_thickness	Inverse variance weighted	14	0.0009	0.0007	0.1857	-0.0004	0.0022
Severe COVID-19 with respiratory failure vs. general population	Mean_medialorbitofrontal_surface_area	MR Egger	14	-2.1363	2.2464	0.3604	-6.5392	2.2666
Severe COVID-19 with respiratory failure vs. general population	Mean_medialorbitofrontal_surface_area	Weighted median	14	-1.2077	1.2564	0.3364	-3.6703	1.2549

continued:

COVID-19 phenotype	Brain structure	Method	nsnp	β	se	p value	β_low95%CI	β_up95%CI
Severe COVID-19 with respiratory failure vs. general population	Mean_medialorbitofrontal_surface_area	Inverse variance weighted	14	-0.5007	0.9203	0.5864	-2.3044	1.3030
Severe COVID-19 with respiratory failure vs. general population	Mean_medialorbitofrontal_thickness	MR Egger	14	-0.0028	0.0023	0.2623	-0.0073	0.0018
Severe COVID-19 with respiratory failure vs. general population	Mean_medialorbitofrontal_thickness	Weighted median	14	0.0001	0.0013	0.9314	-0.0024	0.0026
Severe COVID-19 with respiratory failure vs. general population	Mean_medialorbitofrontal_thickness	Inverse variance weighted	14	-0.0007	0.0010	0.4791	-0.0025	0.0012
Severe COVID-19 with respiratory failure vs. general population	Mean_middletemporal_surface_area	MR Egger	14	4.8905	4.0272	0.2480	-3.0029	12.7838
Severe COVID-19 with respiratory failure vs. general population	Mean_middletemporal_surface_area	Weighted median	14	-0.6633	2.1616	0.7589	-4.9001	3.5735
Severe COVID-19 with respiratory failure vs. general population	Mean_middletemporal_surface_area	Inverse variance weighted	14	-0.8909	1.6508	0.5894	-4.1264	2.3446
Severe COVID-19 with respiratory failure vs. general population	Mean_middletemporal_thickness	MR Egger	14	-0.0012	0.0018	0.5188	-0.0047	0.0023
Severe COVID-19 with respiratory failure vs. general population	Mean_middletemporal_thickness	Weighted median	14	-0.0014	0.0010	0.1635	-0.0033	0.0006
Severe COVID-19 with respiratory failure vs. general population	Mean_middletemporal_thickness	Inverse variance weighted	14	-0.0006	0.0007	0.3886	-0.0021	0.0008
Severe COVID-19 with respiratory failure vs. general population	Mean_paracentral_surface_area	MR Egger	14	5.2495	2.8111	0.0865	-0.2603	10.7594
Severe COVID-19 with respiratory failure vs. general population	Mean_paracentral_surface_area	Weighted median	14	1.8378	1.4790	0.2140	-1.0612	4.7367
Severe COVID-19 with respiratory failure vs. general population	Mean_paracentral_surface_area	Inverse variance weighted	14	2.0977	1.1732	0.0738	-0.2017	4.3971
Severe COVID-19 with respiratory failure vs. general population	Mean_paracentral_thickness	MR Egger	14	-0.0005	0.0024	0.8221	-0.0052	0.0041
Severe COVID-19 with respiratory failure vs. general population	Mean_paracentral_thickness	Weighted median	14	0.0000	0.0011	1.0000	-0.0022	0.0022
Severe COVID-19 with respiratory failure vs. general population	Mean_paracentral_thickness	Inverse variance weighted	14	-0.0005	0.0009	0.6182	-0.0023	0.0014
Severe COVID-19 with respiratory failure vs. general population	Mean_parahippocampal_surface_area	MR Egger	14	-1.8453	1.2705	0.1720	-4.3354	0.6448
Severe COVID-19 with respiratory failure vs. general population	Mean_parahippocampal_surface_area	Weighted median	14	-0.4868	0.7234	0.5010	-1.9046	0.9311
Severe COVID-19 with respiratory failure vs. general population	Mean_parahippocampal_surface_area	Inverse variance weighted	14	-0.6891	0.5193	0.1845	-1.7069	0.3287
Severe COVID-19 with respiratory failure vs. general population	Mean_parahippocampal_thickness	MR Egger	14	0.0051	0.0055	0.3714	-0.0057	0.0160
Severe COVID-19 with respiratory failure vs. general population	Mean_parahippocampal_thickness	Weighted median	14	0.0015	0.0027	0.5813	-0.0038	0.0069
Severe COVID-19 with respiratory failure vs. general population	Mean_parahippocampal_thickness	Inverse variance weighted	14	0.0017	0.0022	0.4495	-0.0027	0.0060
Severe COVID-19 with respiratory failure vs. general population	Mean_parsopercularis_surface_area	MR Egger	14	0.5334	2.8934	0.8568	-5.1377	6.2045
Severe COVID-19 with respiratory failure vs. general population	Mean_parsopercularis_surface_area	Weighted median	14	0.1551	1.5919	0.9224	-2.9650	3.2752
Severe COVID-19 with respiratory failure vs. general population	Mean_parsopercularis_surface_area	Inverse variance weighted	14	0.9131	1.1855	0.4412	-1.4105	3.2367
Severe COVID-19 with respiratory failure vs. general population	Mean_parsopercularis_thickness	MR Egger	14	-0.0013	0.0021	0.5504	-0.0054	0.0028
Severe COVID-19 with respiratory failure vs. general population	Mean_parsopercularis_thickness	Weighted median	14	-0.0011	0.0010	0.2478	-0.0030	0.0008
Severe COVID-19 with respiratory failure vs. general population	Mean_parsopercularis_thickness	Inverse variance weighted	14	-0.0012	0.0008	0.1311	-0.0028	0.0004
Severe COVID-19 with respiratory failure vs. general population	Mean_parsorbitalis_surface_area	MR Egger	14	-0.2203	1.0836	0.8423	-2.3441	1.9035
Severe COVID-19 with respiratory failure vs. general population	Mean_parsorbitalis_surface_area	Weighted median	14	0.3019	0.5781	0.6015	-0.8311	1.4349

continued:

COVID-19 phenotype	Brain structure	Method	nsnp	β	se	p value	β_low95%CI	β_up95%CI
Severe COVID-19 with respiratory failure vs. general population	Mean_parsorbitalis_surface_area	Inverse variance weighted	14	-0.2815	0.4266	0.5094	-1.1176	0.5546
Severe COVID-19 with respiratory failure vs. general population	Mean_parsorbitalis_thickness	MR Egger	14	0.0005	0.0025	0.8541	-0.0044	0.0053
Severe COVID-19 with respiratory failure vs. general population	Mean_parsorbitalis_thickness	Weighted median	14	-0.0004	0.0015	0.8087	-0.0032	0.0025
Severe COVID-19 with respiratory failure vs. general population	Mean_parsorbitalis_thickness	Inverse variance weighted	14	-0.0013	0.0010	0.2045	-0.0032	0.0007
Severe COVID-19 with respiratory failure vs. general population	Mean_parstriangularis_surface_area	MR Egger	14	-5.9435	3.4171	0.1075	-12.6409	0.7539
Severe COVID-19 with respiratory failure vs. general population	Mean_parstriangularis_surface_area	Weighted median	14	-0.1310	1.6991	0.9386	-3.4612	3.1992
Severe COVID-19 with respiratory failure vs. general population	Mean_parstriangularis_surface_area	Inverse variance weighted	14	0.8991	1.5932	0.5725	-2.2237	4.0218
Severe COVID-19 with respiratory failure vs. general population	Mean_parstriangularis_thickness	MR Egger	14	0.0014	0.0017	0.4136	-0.0019	0.0048
Severe COVID-19 with respiratory failure vs. general population	Mean_parstriangularis_thickness	Weighted median	14	-0.0005	0.0009	0.5764	-0.0023	0.0013
Severe COVID-19 with respiratory failure vs. general population	Mean_parstriangularis_thickness	Inverse variance weighted	14	-0.0008	0.0007	0.2595	-0.0021	0.0006
Severe COVID-19 with respiratory failure vs. general population	Mean_pericalcarine_surface_area	MR Egger	14	-6.2882	3.3065	0.0815	-12.7689	0.1924
Severe COVID-19 with respiratory failure vs. general population	Mean_pericalcarine_surface_area	Weighted median	14	-2.3106	1.8425	0.2098	-5.9219	1.3006
Severe COVID-19 with respiratory failure vs. general population	Mean_pericalcarine_surface_area	Inverse variance weighted	14	-2.6628	1.3540	0.0492	-5.3167	-0.0089
Severe COVID-19 with respiratory failure vs. general population	Mean_pericalcarine_thickness	MR Egger	14	0.0034	0.0018	0.0791	-0.0001	0.0068
Severe COVID-19 with respiratory failure vs. general population	Mean_pericalcarine_thickness	Weighted median	14	0.0008	0.0010	0.4358	-0.0012	0.0027
Severe COVID-19 with respiratory failure vs. general population	Mean_pericalcarine_thickness	Inverse variance weighted	14	0.0013	0.0007	0.0741	-0.0001	0.0027
Severe COVID-19 with respiratory failure vs. general population	Mean_postcentral_surface_area	MR Egger	14	-8.3642	4.6927	0.1000	-17.5619	0.8335
Severe COVID-19 with respiratory failure vs. general population	Mean_postcentral_surface_area	Weighted median	14	-0.6151	2.7443	0.8227	-5.9940	4.7638
Severe COVID-19 with respiratory failure vs. general population	Mean_postcentral_surface_area	Inverse variance weighted	14	-0.5276	1.9189	0.7834	-4.2886	3.2335
Severe COVID-19 with respiratory failure vs. general population	Mean_postcentral_thickness	MR Egger	14	0.0001	0.0015	0.9426	-0.0028	0.0030
Severe COVID-19 with respiratory failure vs. general population	Mean_postcentral_thickness	Weighted median	14	0.0006	0.0008	0.4631	-0.0010	0.0022
Severe COVID-19 with respiratory failure vs. general population	Mean_postcentral_thickness	Inverse variance weighted	14	0.0000	0.0006	0.9819	-0.0012	0.0012
Severe COVID-19 with respiratory failure vs. general population	Mean_posteriorcingulate_surface_area	MR Egger	14	-1.3648	1.8895	0.4840	-5.0682	2.3387
Severe COVID-19 with respiratory failure vs. general population	Mean_posteriorcingulate_surface_area	Weighted median	14	0.1041	1.0535	0.9213	-1.9608	2.1690
Severe COVID-19 with respiratory failure vs. general population	Mean_posteriorcingulate_surface_area	Inverse variance weighted	14	0.0209	0.7730	0.9784	-1.4942	1.5360
Severe COVID-19 with respiratory failure vs. general population	Mean_posteriorcingulate_thickness	MR Egger	14	0.0029	0.0020	0.1837	-0.0011	0.0069
Severe COVID-19 with respiratory failure vs. general population	Mean_posteriorcingulate_thickness	Weighted median	14	0.0002	0.0011	0.8586	-0.0020	0.0024
Severe COVID-19 with respiratory failure vs. general population	Mean_posteriorcingulate_thickness	Inverse variance weighted	14	-0.0004	0.0009	0.6731	-0.0021	0.0014
Severe COVID-19 with respiratory failure vs. general population	Mean_precentral_surface_area	MR Egger	14	6.2100	5.5034	0.2812	-4.5766	16.9967
Severe COVID-19 with respiratory failure vs. general population	Mean_precentral_surface_area	Weighted median	14	1.7732	3.1239	0.5703	-4.3497	7.8961

continued:

COVID-19 phenotype	Brain structure	Method	nsnp	β	se	p value	β_low95%CI	β_up95%CI
Severe COVID-19 with respiratory failure vs. general population	Mean_precentral_surface_area	Inverse variance weighted	14	1.3071	2.2534	0.5619	-3.1095	5.7238
Severe COVID-19 with respiratory failure vs. general population	Mean_precentral_thickness	MR Egger	14	0.0008	0.0016	0.6251	-0.0023	0.0039
Severe COVID-19 with respiratory failure vs. general population	Mean_precentral_thickness	Weighted median	14	-0.0005	0.0009	0.5482	-0.0023	0.0012
Severe COVID-19 with respiratory failure vs. general population	Mean_precentral_thickness	Inverse variance weighted	14	-0.0006	0.0006	0.3880	-0.0018	0.0007
Severe COVID-19 with respiratory failure vs. general population	Mean_precuneus_surface_area	MR Egger	14	4.3850	4.7092	0.3701	-4.8450	13.6151
Severe COVID-19 with respiratory failure vs. general population	Mean_precuneus_surface_area	Weighted median	14	3.8425	2.6373	0.1451	-1.3265	9.0115
Severe COVID-19 with respiratory failure vs. general population	Mean_precuneus_surface_area	Inverse variance weighted	14	0.0144	1.9269	0.9941	-3.7624	3.7911
Severe COVID-19 with respiratory failure vs. general population	Mean_precuneus_thickness	MR Egger	14	-0.0019	0.0014	0.1811	-0.0046	0.0007
Severe COVID-19 with respiratory failure vs. general population	Mean_precuneus_thickness	Weighted median	14	-0.0001	0.0008	0.8776	-0.0016	0.0014
Severe COVID-19 with respiratory failure vs. general population	Mean_precuneus_thickness	Inverse variance weighted	14	-0.0003	0.0006	0.6302	-0.0014	0.0008
Severe COVID-19 with respiratory failure vs. general population	Mean_rostralanteriorcingulate_surface_area	MR Egger	14	1.1903	1.5978	0.4707	-1.9415	4.3220
Severe COVID-19 with respiratory failure vs. general population	Mean_rostralanteriorcingulate_surface_area	Weighted median	14	1.1906	0.8821	0.1771	-0.5384	2.9195
Severe COVID-19 with respiratory failure vs. general population	Mean_rostralanteriorcingulate_surface_area	Inverse variance weighted	14	1.0319	0.6534	0.1143	-0.2489	2.3126
Severe COVID-19 with respiratory failure vs. general population	Mean_rostralanteriorcingulate_thickness	MR Egger	14	-0.0006	0.0030	0.8379	-0.0065	0.0053
Severe COVID-19 with respiratory failure vs. general population	Mean_rostralanteriorcingulate_thickness	Weighted median	14	-0.0002	0.0017	0.8984	-0.0035	0.0030
Severe COVID-19 with respiratory failure vs. general population	Mean_rostralanteriorcingulate_thickness	Inverse variance weighted	14	-0.0009	0.0012	0.4855	-0.0033	0.0016
Severe COVID-19 with respiratory failure vs. general population	Mean_rostralmiddlefrontal_surface_area	MR Egger	14	-5.0805	6.6570	0.4601	-18.1283	7.9672
Severe COVID-19 with respiratory failure vs. general population	Mean_rostralmiddlefrontal_surface_area	Weighted median	14	0.4386	3.7808	0.9076	-6.9718	7.8491
Severe COVID-19 with respiratory failure vs. general population	Mean_rostralmiddlefrontal_surface_area	Inverse variance weighted	14	-0.4310	2.7044	0.8734	-5.7316	4.8697
Severe COVID-19 with respiratory failure vs. general population	Mean_rostralmiddlefrontal_thickness	MR Egger	14	-0.0014	0.0014	0.3328	-0.0042	0.0013
Severe COVID-19 with respiratory failure vs. general population	Mean_rostralmiddlefrontal_thickness	Weighted median	14	-0.0012	0.0008	0.1057	-0.0027	0.0003
Severe COVID-19 with respiratory failure vs. general population	Mean_rostralmiddlefrontal_thickness	Inverse variance weighted	14	-0.0014	0.0006	0.0190	-0.0025	-0.0002
Severe COVID-19 with respiratory failure vs. general population	Mean_superiorfrontal_surface_area	MR Egger	14	14.5410	8.4714	0.1117	-2.0628	31.1449
Severe COVID-19 with respiratory failure vs. general population	Mean_superiorfrontal_surface_area	Weighted median	14	1.3357	3.8991	0.7319	-6.3065	8.9779
Severe COVID-19 with respiratory failure vs. general population	Mean_superiorfrontal_surface_area	Inverse variance weighted	14	3.8637	3.5882	0.2816	-3.1691	10.8964
Severe COVID-19 with respiratory failure vs. general population	Mean_superiorfrontal_thickness	MR Egger	14	-0.0008	0.0015	0.6066	-0.0037	0.0021
Severe COVID-19 with respiratory failure vs. general population	Mean_superiorfrontal_thickness	Weighted median	14	-0.0015	0.0008	0.0670	-0.0032	0.0001
Severe COVID-19 with respiratory failure vs. general population	Mean_superiorfrontal_thickness	Inverse variance weighted	14	-0.0010	0.0006	0.1030	-0.0022	0.0002
Severe COVID-19 with respiratory failure vs. general population	Mean_superiorparietal_surface_area	MR Egger	14	-9.1645	6.7283	0.1982	-22.3520	4.0230
Severe COVID-19 with respiratory failure vs. general population	Mean_superiorparietal_surface_area	Weighted median	14	-5.4977	3.6999	0.1373	-12.7495	1.7541

continued:

COVID-19 phenotype	Brain structure	Method	nsnp	β	se	p value	β_low95%CI	β_up95%CI
Severe COVID-19 with respiratory failure vs. general population	Mean_superiorparietal_surface_area	Inverse variance weighted	14	-5.6310	2.7532	0.0408	-11.0273	-0.2348
Severe COVID-19 with respiratory failure vs. general population	Mean_superiorparietal_thickness	MR Egger	14	-0.0010	0.0013	0.4500	-0.0036	0.0016
Severe COVID-19 with respiratory failure vs. general population	Mean_superiorparietal_thickness	Weighted median	14	-0.0008	0.0007	0.2577	-0.0022	0.0006
Severe COVID-19 with respiratory failure vs. general population	Mean_superiorparietal_thickness	Inverse variance weighted	14	0.0003	0.0005	0.6007	-0.0008	0.0013
Severe COVID-19 with respiratory failure vs. general population	Mean_superiortemporal_surface_area	MR Egger	14	5.4744	3.9378	0.1897	-2.2437	13.1924
Severe COVID-19 with respiratory failure vs. general population	Mean_superiortemporal_surface_area	Weighted median	14	2.3401	2.2849	0.3058	-2.1384	6.8185
Severe COVID-19 with respiratory failure vs. general population	Mean_superiortemporal_surface_area	Inverse variance weighted	14	1.8176	1.6172	0.2610	-1.3521	4.9874
Severe COVID-19 with respiratory failure vs. general population	Mean_superiortemporal_thickness	MR Egger	14	0.0019	0.0017	0.4048	-0.0015	0.0053
Severe COVID-19 with respiratory failure vs. general population	Mean_superiortemporal_thickness	Weighted median	14	0.0020	0.0015	0.0849	-0.0009	0.0049
Severe COVID-19 with respiratory failure vs. general population	Mean_superiortemporal_thickness	Inverse variance weighted	14	0.0022	0.0008	0.0675	0.0007	0.0037
Severe COVID-19 with respiratory failure vs. general population	Mean_supramarginal_surface_area	MR Egger	14	0.5115	5.3826	0.9259	-10.0383	11.0614
Severe COVID-19 with respiratory failure vs. general population	Mean_supramarginal_surface_area	Weighted median	14	2.8860	2.8276	0.3074	-2.6560	8.4281
Severe COVID-19 with respiratory failure vs. general population	Mean_supramarginal_surface_area	Inverse variance weighted	14	2.8022	2.2070	0.2042	-1.5236	7.1281
Severe COVID-19 with respiratory failure vs. general population	Mean_supramarginal_thickness	MR Egger	14	-0.0007	0.0014	0.5956	-0.0034	0.0019
Severe COVID-19 with respiratory failure vs. general population	Mean_supramarginal_thickness	Weighted median	14	-0.0001	0.0007	0.8836	-0.0016	0.0014
Severe COVID-19 with respiratory failure vs. general population	Mean_supramarginal_thickness	Inverse variance weighted	14	0.0001	0.0005	0.8555	-0.0010	0.0012
Severe COVID-19 with respiratory failure vs. general population	Mean_temporalpole_surface_area	MR Egger	14	-0.8429	0.7847	0.3039	-2.3809	0.6951
Severe COVID-19 with respiratory failure vs. general population	Mean_temporalpole_surface_area	Weighted median	14	0.0672	0.4219	0.8735	-0.7598	0.8941
Severe COVID-19 with respiratory failure vs. general population	Mean_temporalpole_surface_area	Inverse variance weighted	14	0.2125	0.3217	0.5088	-0.4179	0.8430
Severe COVID-19 with respiratory failure vs. general population	Mean_temporalpole_thickness	MR Egger	14	0.0068	0.0052	0.2171	-0.0034	0.0170
Severe COVID-19 with respiratory failure vs. general population	Mean_temporalpole_thickness	Weighted median	14	0.0018	0.0026	0.4794	-0.0032	0.0068
Severe COVID-19 with respiratory failure vs. general population	Mean_temporalpole_thickness	Inverse variance weighted	14	0.0016	0.0021	0.4525	-0.0026	0.0058
Severe COVID-19 with respiratory failure vs. general population	Mean_transversetemporal_surface_area	MR Egger	14	-0.1088	0.9332	0.9091	-1.9378	1.7202
Severe COVID-19 with respiratory failure vs. general population	Mean_transversetemporal_surface_area	Weighted median	14	-0.0743	0.5001	0.8818	-1.0545	0.9058
Severe COVID-19 with respiratory failure vs. general population	Mean_transversetemporal_surface_area	Inverse variance weighted	14	0.2702	0.3698	0.4651	-0.4547	0.9950
Severe COVID-19 with respiratory failure vs. general population	Mean_transversetemporal_thickness	MR Egger	14	0.0001	0.0032	0.9865	-0.0062	0.0063
Severe COVID-19 with respiratory failure vs. general population	Mean_transversetemporal_thickness	Weighted median	14	0.0010	0.0016	0.5301	-0.0022	0.0042
Severe COVID-19 with respiratory failure vs. general population	Mean_transversetemporal_thickness	Inverse variance weighted	14	0.0008	0.0013	0.5077	-0.0016	0.0033
Severe COVID-19 with respiratory failure vs. general population	Mean_Amygdala_volume	MR Egger	13	8.6498	5.7082	0.1579	-2.5383	19.8380
Severe COVID-19 with respiratory failure vs. general population	Mean_Amygdala_volume	Weighted median	13	4.0757	3.0547	0.1821	-1.9116	10.0630

continued:

COVID-19 phenotype	Brain structure	Method	nsnp	β	se	p value	β_low95%CI	β_up95%CI
Severe COVID-19 with respiratory failure vs. general population	Mean_Amygdala_volume	Inverse variance weighted	13	3.8575	2.2975	0.0932	-0.6456	8.3606
Severe COVID-19 with respiratory failure vs. general population	Mean_Caudate_volume	MR Egger	13	9.4734	10.7709	0.3979	-11.6377	30.5844
Severe COVID-19 with respiratory failure vs. general population	Mean_Caudate_volume	Weighted median	13	5.3520	5.8615	0.3612	-6.1364	16.8405
Severe COVID-19 with respiratory failure vs. general population	Mean_Caudate_volume	Inverse variance weighted	13	5.1994	4.3682	0.2339	-3.3623	13.7610
Severe COVID-19 with respiratory failure vs. general population	Mean_Hippocampus_volume	MR Egger	13	12.5663	13.3016	0.3651	-13.5049	38.6374
Severe COVID-19 with respiratory failure vs. general population	Mean_Hippocampus_volume	Weighted median	13	3.5554	5.6992	0.5327	-7.6151	14.7259
Severe COVID-19 with respiratory failure vs. general population	Mean_Hippocampus_volume	Inverse variance weighted	13	4.5058	5.2705	0.3926	-5.8244	14.8360
Severe COVID-19 with respiratory failure vs. general population	Mean_Intracranial_volume	MR Egger	14	-5411.7467	4070.7259	0.2084	-13390.3695	2566.8761
Severe COVID-19 with respiratory failure vs. general population	Mean_Intracranial_volume	Weighted median	14	-1374.1739	2269.8505	0.5449	-5823.0808	3074.7330
Severe COVID-19 with respiratory failure vs. general population	Mean_Intracranial_volume	Inverse variance weighted	14	-992.2501	1684.9083	0.5559	-4294.6703	2310.1701
Severe COVID-19 with respiratory failure vs. general population	Mean_Nucleus_accumbens_volume	MR Egger	13	2.0594	2.3059	0.3903	-2.4602	6.5790
Severe COVID-19 with respiratory failure vs. general population	Mean_Nucleus_accumbens_volume	Weighted median	13	2.1985	1.5929	0.1838	-0.9235	5.3205
Severe COVID-19 with respiratory failure vs. general population	Mean_Nucleus_accumbens_volume	Inverse variance weighted	13	2.5214	0.9385	0.0539	0.6820	4.3609
Severe COVID-19 with respiratory failure vs. general population	Mean_Pallidum_volume	MR Egger	13	3.0003	4.2355	0.4935	-5.3014	11.3019
Severe COVID-19 with respiratory failure vs. general population	Mean_Pallidum_volume	Weighted median	13	1.3235	2.2581	0.5578	-3.1023	5.7494
Severe COVID-19 with respiratory failure vs. general population	Mean_Pallidum_volume	Inverse variance weighted	13	1.3898	1.7146	0.4176	-1.9707	4.7504
Severe COVID-19 with respiratory failure vs. general population	Mean_Putamen_volume	MR Egger	13	5.7765	16.1839	0.7279	-25.9438	37.4969
Severe COVID-19 with respiratory failure vs. general population	Mean_Putamen_volume	Weighted median	13	4.9503	7.3640	0.5014	-9.4831	19.3837
Severe COVID-19 with respiratory failure vs. general population	Mean_Putamen_volume	Inverse variance weighted	13	7.6786	6.2754	0.2211	-4.6211	19.9784
Severe COVID-19 with respiratory failure vs. general population	Mean_Thalamus_volume	MR Egger	13	20.8519	13.7319	0.1571	-6.0626	47.7664
Severe COVID-19 with respiratory failure vs. general population	Mean_Thalamus_volume	Weighted median	13	6.4233	7.4896	0.3911	-8.2564	21.1030
Severe COVID-19 with respiratory failure vs. general population	Mean_Thalamus_volume	Inverse variance weighted	13	6.3854	5.5837	0.2528	-4.5586	17.3293

Abbreviations: COVID-19, coronavirus disease 2019; CI, confidence interval; nSNPs, number of single-nucleotide polymorphisms; se, standard error

Table S7. MR analysis of the causal relationship between critical COVID-19 and brain structure

COVID-19 phenotype	Brain structure	Method	nsnp	β	se	p value	β_low95%CI	β_up95%CI
Critical COVID-19 vs. general population	Mean_bankssts_surface_area	MR Egger	93	0.1232	0.2979	0.6802	-0.4606	0.7070
Critical COVID-19 vs. general population	Mean_bankssts_surface_area	Inverse variance weighted	93	-0.2268	0.0907	0.0124	-0.4045	-0.0490
Critical COVID-19 vs. general population	Mean_bankssts_surface_area	Weighted median	93	-0.1941	0.1283	0.1302	-0.4456	0.0573
Critical COVID-19 vs. general population	Mean_bankssts_thickness	MR Egger	93	-0.0001	0.0003	0.7547	-0.0008	0.0005
Critical COVID-19 vs. general population	Mean_bankssts_thickness	Weighted median	93	-0.0001	0.0001	0.5931	-0.0003	0.0002
Critical COVID-19 vs. general population	Mean_bankssts_thickness	Inverse variance weighted	93	-0.0001	0.0001	0.3662	-0.0003	0.0001
Critical COVID-19 vs. general population	Mean_caudalanteriorcingulate_surface_area	MR Egger	93	0.2641	0.2703	0.3310	-0.2656	0.7939
Critical COVID-19 vs. general population	Mean_caudalanteriorcingulate_surface_area	Weighted median	93	0.0427	0.1132	0.7064	-0.1792	0.2645
Critical COVID-19 vs. general population	Mean_caudalanteriorcingulate_surface_area	Inverse variance weighted	93	-0.0272	0.0821	0.7403	-0.1882	0.1338
Critical COVID-19 vs. general population	Mean_caudalanteriorcingulate_thickness	MR Egger	93	0.0016	0.0005	0.0044	0.0005	0.0026
Critical COVID-19 vs. general population	Mean_caudalanteriorcingulate_thickness	Weighted median	93	0.0001	0.0002	0.8062	-0.0004	0.0005
Critical COVID-19 vs. general population	Mean_caudalanteriorcingulate_thickness	Inverse variance weighted	93	0.0001	0.0002	0.5680	-0.0002	0.0004
Critical COVID-19 vs. general population	Mean_caudalmiddlefrontal_surface_area	MR Egger	93	-0.2121	0.8084	0.7936	-1.7965	1.3723
Critical COVID-19 vs. general population	Mean_caudalmiddlefrontal_surface_area	Weighted median	93	0.1630	0.3199	0.6103	-0.4640	0.7901
Critical COVID-19 vs. general population	Mean_caudalmiddlefrontal_surface_area	Inverse variance weighted	93	0.0718	0.2443	0.7688	-0.4071	0.5507
Critical COVID-19 vs. general population	Mean_caudalmiddlefrontal_thickness	MR Egger	93	-0.0004	0.0002	0.1245	-0.0009	0.0001
Critical COVID-19 vs. general population	Mean_caudalmiddlefrontal_thickness	Weighted median	93	0.0000	0.0001	0.7375	-0.0002	0.0002
Critical COVID-19 vs. general population	Mean_caudalmiddlefrontal_thickness	Inverse variance weighted	93	0.0000	0.0001	0.5655	-0.0001	0.0002
Critical COVID-19 vs. general population	Mean_cuneus_surface_area	MR Egger	93	-0.5156	0.4099	0.2116	-1.3190	0.2877
Critical COVID-19 vs. general population	Mean_cuneus_surface_area	Weighted median	93	0.0596	0.1780	0.7380	-0.2894	0.4085
Critical COVID-19 vs. general population	Mean_cuneus_surface_area	Inverse variance weighted	93	-0.0592	0.1246	0.6347	-0.3034	0.1850
Critical COVID-19 vs. general population	Mean_cuneus_thickness	MR Egger	93	0.0003	0.0003	0.3567	-0.0003	0.0010
Critical COVID-19 vs. general population	Mean_cuneus_thickness	Weighted median	93	0.0001	0.0001	0.3813	-0.0001	0.0004
Critical COVID-19 vs. general population	Mean_cuneus_thickness	Inverse variance weighted	93	-0.0001	0.0001	0.5960	-0.0003	0.0001
Critical COVID-19 vs. general population	Mean_entorhinal_surface_area	MR Egger	93	0.2934	0.1619	0.0732	-0.0239	0.6106
Critical COVID-19 vs. general population	Mean_entorhinal_surface_area	Weighted median	93	0.0987	0.0721	0.1714	-0.0427	0.2401
Critical COVID-19 vs. general population	Mean_entorhinal_surface_area	Inverse variance weighted	93	0.0357	0.0497	0.4731	-0.0617	0.1331
Critical COVID-19 vs. general population	Mean_entorhinal_thickness	MR Egger	93	-0.0003	0.0008	0.6952	-0.0020	0.0013
Critical COVID-19 vs. general population	Mean_entorhinal_thickness	Weighted median	93	0.0000	0.0004	0.9186	-0.0008	0.0007
Critical COVID-19 vs. general population	Mean_entorhinal_thickness	Inverse variance weighted	93	0.0000	0.0003	0.9652	-0.0005	0.0005

continued:

COVID-19 phenotype	Brain structure	Method	nsnp	β	se	p value	β_low95%CI	β_up95%CI
Critical COVID-19 vs. general population	Mean_frontalpole_surface_area	MR Egger	93	0.0227	0.0864	0.7929	-0.1466	0.1920
Critical COVID-19 vs. general population	Mean_frontalpole_surface_area	Weighted median	93	-0.0167	0.0340	0.6236	-0.0834	0.0500
Critical COVID-19 vs. general population	Mean_frontalpole_surface_area	Inverse variance weighted	93	-0.0236	0.0261	0.3659	-0.0749	0.0276
Critical COVID-19 vs. general population	Mean_frontalpole_thickness	MR Egger	93	0.0005	0.0005	0.3279	-0.0005	0.0016
Critical COVID-19 vs. general population	Mean_frontalpole_thickness	Weighted median	93	0.0003	0.0003	0.2188	-0.0002	0.0008
Critical COVID-19 vs. general population	Mean_frontalpole_thickness	Inverse variance weighted	93	0.0003	0.0002	0.0785	0.0000	0.0006
Critical COVID-19 vs. general population	Mean_full_surface_area	MR Egger	9	-174.1018	203.8708	0.4214	-573.6886	225.4849
Critical COVID-19 vs. general population	Mean_full_surface_area	Weighted median	9	-102.9221	62.0763	0.0973	-224.5917	18.7475
Critical COVID-19 vs. general population	Mean_full_surface_area	Inverse variance weighted	9	-36.6132	59.1534	0.5359	-152.5538	79.3274
Critical COVID-19 vs. general population	Mean_full_thickness	MR Egger	17	0.0005	0.0008	0.5433	-0.0011	0.0021
Critical COVID-19 vs. general population	Mean_full_thickness	Weighted median	17	0.0002	0.0003	0.4978	-0.0004	0.0008
Critical COVID-19 vs. general population	Mean_full_thickness	Inverse variance weighted	17	0.0001	0.0003	0.5705	-0.0004	0.0007
Critical COVID-19 vs. general population	Mean_fusiform_surface_area	MR Egger	93	-0.2389	0.6549	0.7161	-1.5225	1.0446
Critical COVID-19 vs. general population	Mean_fusiform_surface_area	Weighted median	93	-0.3252	0.3007	0.2794	-0.9146	0.2641
Critical COVID-19 vs. general population	Mean_fusiform_surface_area	Inverse variance weighted	93	-0.2019	0.1977	0.3072	-0.5893	0.1856
Critical COVID-19 vs. general population	Mean_fusiform_thickness	MR Egger	93	-0.0002	0.0003	0.5519	-0.0007	0.0004
Critical COVID-19 vs. general population	Mean_fusiform_thickness	Weighted median	93	0.0000	0.0001	1.0000	-0.0002	0.0002
Critical COVID-19 vs. general population	Mean_fusiform_thickness	Inverse variance weighted	93	0.0000	0.0001	0.6748	-0.0002	0.0001
Critical COVID-19 vs. general population	Mean_inferiorparietal_surface_area	MR Egger	93	1.2164	1.2403	0.3293	-1.2145	3.6474
Critical COVID-19 vs. general population	Mean_inferiorparietal_surface_area	Weighted median	93	-0.5631	0.5281	0.2863	-1.5982	0.4719
Critical COVID-19 vs. general population	Mean_inferiorparietal_surface_area	Inverse variance weighted	93	-0.4613	0.3785	0.2229	-1.2031	0.2806
Critical COVID-19 vs. general population	Mean_inferiorparietal_thickness	MR Egger	93	0.0000	0.0002	0.8810	-0.0004	0.0003
Critical COVID-19 vs. general population	Mean_inferiorparietal_thickness	Weighted median	93	0.0001	0.0001	0.1161	0.0000	0.0003
Critical COVID-19 vs. general population	Mean_inferiorparietal_thickness	Inverse variance weighted	93	0.0001	0.0001	0.1552	0.0000	0.0002
Critical COVID-19 vs. general population	Mean_inferiortemporal_surface_area	MR Egger	93	0.9485	0.7550	0.2122	-0.5312	2.4283
Critical COVID-19 vs. general population	Mean_inferiortemporal_surface_area	Weighted median	93	-0.1255	0.3182	0.6933	-0.7493	0.4983
Critical COVID-19 vs. general population	Mean_inferiortemporal_surface_area	Inverse variance weighted	93	0.1552	0.2295	0.4989	-0.2946	0.6050
Critical COVID-19 vs. general population	Mean_inferiortemporal_thickness	MR Egger	93	0.0001	0.0003	0.8010	-0.0005	0.0006
Critical COVID-19 vs. general population	Mean_inferiortemporal_thickness	Weighted median	93	0.0000	0.0001	0.8031	-0.0002	0.0003
Critical COVID-19 vs. general population	Mean_inferiortemporal_thickness	Inverse variance weighted	93	0.0000	0.0001	0.6916	-0.0002	0.0001

continued:

COVID-19 phenotype	Brain structure	Method	nsnp	β	se	p value	β_low95%CI	β_up95%CI
Critical COVID-19 vs. general population	Mean_insula_surface_area	MR Egger	92	0.3427	0.4452	0.4435	-0.5299	1.2154
Critical COVID-19 vs. general population	Mean_insula_surface_area	Weighted median	92	0.3838	0.2056	0.0619	-0.0191	0.7867
Critical COVID-19 vs. general population	Mean_insula_surface_area	Inverse variance weighted	92	0.3832	0.1347	0.0044	0.1192	0.6472
Critical COVID-19 vs. general population	Mean_insula_thickness	MR Egger	93	0.0003	0.0004	0.4842	-0.0004	0.0009
Critical COVID-19 vs. general population	Mean_insula_thickness	Weighted median	93	-0.0001	0.0001	0.4350	-0.0004	0.0002
Critical COVID-19 vs. general population	Mean_insula_thickness	Inverse variance weighted	93	-0.0001	0.0001	0.1984	-0.0003	0.0001
Critical COVID-19 vs. general population	Mean_isthmuscingulate_surface_area	MR Egger	93	-0.6599	0.3150	0.0390	-1.2774	-0.0424
Critical COVID-19 vs. general population	Mean_isthmuscingulate_surface_area	Weighted median	93	-0.1818	0.1318	0.1678	-0.4401	0.0765
Critical COVID-19 vs. general population	Mean_isthmuscingulate_surface_area	Inverse variance weighted	93	-0.0354	0.0973	0.7162	-0.2262	0.1554
Critical COVID-19 vs. general population	Mean_isthmuscingulate_thickness	MR Egger	93	0.0007	0.0005	0.1694	-0.0003	0.0016
Critical COVID-19 vs. general population	Mean_isthmuscingulate_thickness	Weighted median	93	0.0001	0.0002	0.5481	-0.0003	0.0005
Critical COVID-19 vs. general population	Mean_isthmuscingulate_thickness	Inverse variance weighted	93	-0.0001	0.0002	0.4185	-0.0004	0.0002
Critical COVID-19 vs. general population	Mean_lateraloccipital_surface_area	MR Egger	93	0.2361	0.9469	0.8037	-1.6198	2.0920
Critical COVID-19 vs. general population	Mean_lateraloccipital_surface_area	Weighted median	93	0.2882	0.4221	0.4948	-0.5391	1.1154
Critical COVID-19 vs. general population	Mean_lateraloccipital_surface_area	Inverse variance weighted	93	0.3208	0.2874	0.2644	-0.2426	0.8842
Critical COVID-19 vs. general population	Mean_lateraloccipital_thickness	MR Egger	93	0.0000	0.0003	0.8846	-0.0005	0.0005
Critical COVID-19 vs. general population	Mean_lateraloccipital_thickness	Weighted median	93	0.0000	0.0001	1.0000	-0.0002	0.0002
Critical COVID-19 vs. general population	Mean_lateraloccipital_thickness	Inverse variance weighted	93	0.0000	0.0001	0.8363	-0.0001	0.0002
Critical COVID-19 vs. general population	Mean_lateralorbitofrontal_surface_area	MR Egger	93	1.0776	0.4608	0.0215	0.1745	1.9808
Critical COVID-19 vs. general population	Mean_lateralorbitofrontal_surface_area	Weighted median	93	0.1416	0.2073	0.4944	-0.2647	0.5479
Critical COVID-19 vs. general population	Mean_lateralorbitofrontal_surface_area	Inverse variance weighted	93	0.0598	0.1400	0.6691	-0.2146	0.3342
Critical COVID-19 vs. general population	Mean_lateralorbitofrontal_thickness	MR Egger	93	-0.0002	0.0003	0.6040	-0.0008	0.0004
Critical COVID-19 vs. general population	Mean_lateralorbitofrontal_thickness	Weighted median	93	0.0000	0.0001	0.8540	-0.0003	0.0003
Critical COVID-19 vs. general population	Mean_lateralorbitofrontal_thickness	Inverse variance weighted	93	0.0000	0.0001	0.8572	-0.0002	0.0002
Critical COVID-19 vs. general population	Mean_lingual_surface_area	MR Egger	93	-0.7620	0.7696	0.3247	-2.2703	0.7464
Critical COVID-19 vs. general population	Mean_lingual_surface_area	Weighted median	93	-0.3916	0.3421	0.2524	-1.0622	0.2790
Critical COVID-19 vs. general population	Mean_lingual_surface_area	Inverse variance weighted	93	-0.2534	0.2329	0.2766	-0.7099	0.2031
Critical COVID-19 vs. general population	Mean_lingual_thickness	MR Egger	93	-0.0002	0.0002	0.3261	-0.0007	0.0002
Critical COVID-19 vs. general population	Mean_lingual_thickness	Weighted median	93	-0.0001	0.0001	0.3540	-0.0003	0.0001
Critical COVID-19 vs. general population	Mean_lingual_thickness	Inverse variance weighted	93	-0.0001	0.0001	0.0781	-0.0003	0.0000

continued:

COVID-19 phenotype	Brain structure	Method	nsnp	β	se	p value	β_low95%CI	β_up95%CI
Critical COVID-19 vs. general population	Mean_medialorbitofrontal_surface_area	MR Egger	93	0.2852	0.3422	0.4068	-0.3855	0.9558
Critical COVID-19 vs. general population	Mean_medialorbitofrontal_surface_area	Weighted median	93	0.0978	0.1559	0.5302	-0.2076	0.4033
Critical COVID-19 vs. general population	Mean_medialorbitofrontal_surface_area	Inverse variance weighted	93	0.0478	0.1039	0.6456	-0.1559	0.2514
Critical COVID-19 vs. general population	Mean_medialorbitofrontal_thickness	MR Egger	93	0.0003	0.0003	0.3498	-0.0003	0.0010
Critical COVID-19 vs. general population	Mean_medialorbitofrontal_thickness	Weighted median	93	0.0001	0.0002	0.6957	-0.0002	0.0004
Critical COVID-19 vs. general population	Mean_medialorbitofrontal_thickness	Inverse variance weighted	93	0.0000	0.0001	0.9832	-0.0002	0.0002
Critical COVID-19 vs. general population	Mean_middletemporal_surface_area	MR Egger	92	-1.3503	0.7596	0.0788	-2.8391	0.1385
Critical COVID-19 vs. general population	Mean_middletemporal_surface_area	Weighted median	92	-0.7946	0.2807	0.0047	-1.3448	-0.2443
Critical COVID-19 vs. general population	Mean_middletemporal_surface_area	Inverse variance weighted	92	-0.5261	0.2312	0.0229	-0.9793	-0.0729
Critical COVID-19 vs. general population	Mean_middletemporal_thickness	MR Egger	92	-0.0003	0.0003	0.3223	-0.0008	0.0003
Critical COVID-19 vs. general population	Mean_middletemporal_thickness	Weighted median	92	0.0000	0.0001	0.9766	-0.0002	0.0002
Critical COVID-19 vs. general population	Mean_middletemporal_thickness	Inverse variance weighted	92	-0.0002	0.0001	0.0462	-0.0003	0.0000
Critical COVID-19 vs. general population	Mean_paracentral_surface_area	MR Egger	93	-0.0730	0.4380	0.8681	-0.9315	0.7856
Critical COVID-19 vs. general population	Mean_paracentral_surface_area	Weighted median	93	0.0257	0.1681	0.8787	-0.3039	0.3552
Critical COVID-19 vs. general population	Mean_paracentral_surface_area	Inverse variance weighted	93	0.0943	0.1322	0.4760	-0.1649	0.3535
Critical COVID-19 vs. general population	Mean_paracentral_thickness	MR Egger	93	0.0000	0.0003	0.9767	-0.0006	0.0006
Critical COVID-19 vs. general population	Mean_paracentral_thickness	Weighted median	93	0.0000	0.0001	0.8218	-0.0003	0.0002
Critical COVID-19 vs. general population	Mean_paracentral_thickness	Inverse variance weighted	93	0.0000	0.0001	0.9307	-0.0002	0.0002
Critical COVID-19 vs. general population	Mean_parahippocampal_surface_area	MR Egger	92	-0.3252	0.2233	0.1486	-0.7628	0.1123
Critical COVID-19 vs. general population	Mean_parahippocampal_surface_area	Weighted median	92	-0.1447	0.0958	0.1311	-0.3325	0.0432
Critical COVID-19 vs. general population	Mean_parahippocampal_surface_area	Inverse variance weighted	92	-0.1473	0.0678	0.0297	-0.2802	-0.0145
Critical COVID-19 vs. general population	Mean_parahippocampal_thickness	MR Egger	93	0.0010	0.0007	0.1993	-0.0005	0.0024
Critical COVID-19 vs. general population	Mean_parahippocampal_thickness	Weighted median	93	0.0004	0.0004	0.2244	-0.0003	0.0012
Critical COVID-19 vs. general population	Mean_parahippocampal_thickness	Inverse variance weighted	93	0.0005	0.0002	0.0533	0.0000	0.0009
Critical COVID-19 vs. general population	Mean_parsopercularis_surface_area	MR Egger	93	0.5197	0.4562	0.2577	-0.3745	1.4139
Critical COVID-19 vs. general population	Mean_parsopercularis_surface_area	Weighted median	93	0.0095	0.2072	0.9633	-0.3965	0.4156
Critical COVID-19 vs. general population	Mean_parsopercularis_surface_area	Inverse variance weighted	93	-0.0002	0.1389	0.9990	-0.2725	0.2721
Critical COVID-19 vs. general population	Mean_parsopercularis_thickness	MR Egger	93	0.0000	0.0002	0.9225	-0.0004	0.0005
Critical COVID-19 vs. general population	Mean_parsopercularis_thickness	Weighted median	93	0.0000	0.0001	1.0000	-0.0002	0.0002
Critical COVID-19 vs. general population	Mean_parsopercularis_thickness	Inverse variance weighted	93	0.0000	0.0001	0.6364	-0.0001	0.0002

continued:

COVID-19 phenotype	Brain structure	Method	nsnp	β	se	p value	β_low95%CI	β_up95%CI
Critical COVID-19 vs. general population	Mean_parsorbitalis_surface_area	MR Egger	93	0.1955	0.1574	0.2174	-0.1130	0.5040
Critical COVID-19 vs. general population	Mean_parsorbitalis_surface_area	Weighted median	93	-0.1078	0.0684	0.1152	-0.2420	0.0263
Critical COVID-19 vs. general population	Mean_parsorbitalis_surface_area	Inverse variance weighted	93	-0.0819	0.0484	0.0908	-0.1768	0.0130
Critical COVID-19 vs. general population	Mean_parsorbitalis_thickness	MR Egger	93	-0.0003	0.0004	0.4470	-0.0010	0.0004
Critical COVID-19 vs. general population	Mean_parsorbitalis_thickness	Weighted median	93	0.0001	0.0002	0.5305	-0.0002	0.0004
Critical COVID-19 vs. general population	Mean_parsorbitalis_thickness	Inverse variance weighted	93	-0.0001	0.0001	0.4717	-0.0003	0.0001
Critical COVID-19 vs. general population	Mean_parstriangularis_surface_area	MR Egger	93	0.5094	0.4356	0.2454	-0.3445	1.3632
Critical COVID-19 vs. general population	Mean_parstriangularis_surface_area	Weighted median	93	0.1897	0.1877	0.3121	-0.1782	0.5576
Critical COVID-19 vs. general population	Mean_parstriangularis_surface_area	Inverse variance weighted	93	0.1602	0.1322	0.2256	-0.0989	0.4192
Critical COVID-19 vs. general population	Mean_parstriangularis_thickness	MR Egger	93	0.0001	0.0003	0.5786	-0.0004	0.0007
Critical COVID-19 vs. general population	Mean_parstriangularis_thickness	Weighted median	93	-0.0001	0.0001	0.6295	-0.0003	0.0002
Critical COVID-19 vs. general population	Mean_parstriangularis_thickness	Inverse variance weighted	93	0.0000	0.0001	0.5815	-0.0002	0.0001
Critical COVID-19 vs. general population	Mean_pericalcarine_surface_area	MR Egger	93	-1.1900	0.6423	0.0672	-2.4489	0.0690
Critical COVID-19 vs. general population	Mean_pericalcarine_surface_area	Weighted median	93	-0.2155	0.2436	0.3764	-0.6930	0.2620
Critical COVID-19 vs. general population	Mean_pericalcarine_surface_area	Inverse variance weighted	93	-0.0700	0.1974	0.7227	-0.4570	0.3169
Critical COVID-19 vs. general population	Mean_pericalcarine_thickness	MR Egger	93	0.0001	0.0003	0.6885	-0.0005	0.0007
Critical COVID-19 vs. general population	Mean_pericalcarine_thickness	Weighted median	93	0.0000	0.0001	1.0000	-0.0002	0.0002
Critical COVID-19 vs. general population	Mean_pericalcarine_thickness	Inverse variance weighted	93	-0.0001	0.0001	0.4944	-0.0002	0.0001
Critical COVID-19 vs. general population	Mean_postcentral_surface_area	MR Egger	93	0.1330	0.7594	0.8613	-1.3554	1.6215
Critical COVID-19 vs. general population	Mean_postcentral_surface_area	Weighted median	93	0.0382	0.3430	0.9114	-0.6341	0.7104
Critical COVID-19 vs. general population	Mean_postcentral_surface_area	Inverse variance weighted	93	-0.2833	0.2294	0.2168	-0.7330	0.1663
Critical COVID-19 vs. general population	Mean_postcentral_thickness	MR Egger	93	0.0000	0.0002	0.9458	-0.0004	0.0005
Critical COVID-19 vs. general population	Mean_postcentral_thickness	Weighted median	93	-0.0001	0.0001	0.4606	-0.0003	0.0001
Critical COVID-19 vs. general population	Mean_postcentral_thickness	Inverse variance weighted	93	0.0000	0.0001	0.8721	-0.0001	0.0001
Critical COVID-19 vs. general population	Mean_posteriorcingulate_surface_area	MR Egger	93	-0.1127	0.3096	0.7168	-0.7194	0.4941
Critical COVID-19 vs. general population	Mean_posteriorcingulate_surface_area	Weighted median	93	-0.0462	0.1336	0.7293	-0.3080	0.2156
Critical COVID-19 vs. general population	Mean_posteriorcingulate_surface_area	Inverse variance weighted	93	-0.0772	0.0934	0.4085	-0.2603	0.1059
Critical COVID-19 vs. general population	Mean_posteriorcingulate_thickness	MR Egger	93	0.0003	0.0003	0.2515	-0.0002	0.0009
Critical COVID-19 vs. general population	Mean_posteriorcingulate_thickness	Weighted median	93	0.0001	0.0001	0.4028	-0.0002	0.0004
Critical COVID-19 vs. general population	Mean_posteriorcingulate_thickness	Inverse variance weighted	93	0.0000	0.0001	0.9916	-0.0002	0.0002

continued:

COVID-19 phenotype	Brain structure	Method	nsnp	β	se	p value	β_low95%CI	β_up95%CI
Critical COVID-19 vs. general population	Mean_precentral_surface_area	MR Egger	93	-0.8720	0.9186	0.3450	-2.6724	0.9284
Critical COVID-19 vs. general population	Mean_precentral_surface_area	Weighted median	93	-0.4163	0.4037	0.3024	-1.2076	0.3750
Critical COVID-19 vs. general population	Mean_precentral_surface_area	Inverse variance weighted	93	-0.4032	0.2777	0.1466	-0.9476	0.1412
Critical COVID-19 vs. general population	Mean_precentral_thickness	MR Egger	93	-0.0004	0.0003	0.1479	-0.0009	0.0001
Critical COVID-19 vs. general population	Mean_precentral_thickness	Weighted median	93	0.0000	0.0001	0.9925	-0.0002	0.0002
Critical COVID-19 vs. general population	Mean_precentral_thickness	Inverse variance weighted	93	0.0000	0.0001	0.7389	-0.0001	0.0002
Critical COVID-19 vs. general population	Mean_precuneus_surface_area	MR Egger	93	-0.5359	0.7953	0.5021	-2.0948	1.0229
Critical COVID-19 vs. general population	Mean_precuneus_surface_area	Weighted median	93	0.1170	0.3537	0.7408	-0.5763	0.8103
Critical COVID-19 vs. general population	Mean_precuneus_surface_area	Inverse variance weighted	93	-0.0839	0.2405	0.7274	-0.5552	0.3875
Critical COVID-19 vs. general population	Mean_precuneus_thickness	MR Egger	93	0.0000	0.0002	0.8218	-0.0005	0.0004
Critical COVID-19 vs. general population	Mean_precuneus_thickness	Weighted median	93	0.0000	0.0001	1.0000	-0.0002	0.0002
Critical COVID-19 vs. general population	Mean_precuneus_thickness	Inverse variance weighted	93	0.0000	0.0001	0.4909	-0.0002	0.0001
Critical COVID-19 vs. general population	Mean_rostralanteriorcingulate_surface_area	MR Egger	93	0.6125	0.2599	0.0206	0.1031	1.1220
Critical COVID-19 vs. general population	Mean_rostralanteriorcingulate_surface_area	Weighted median	93	0.0724	0.1057	0.4936	-0.1348	0.2796
Critical COVID-19 vs. general population	Mean_rostralanteriorcingulate_surface_area	Inverse variance weighted	93	0.0796	0.0804	0.3225	-0.0781	0.2373
Critical COVID-19 vs. general population	Mean_rostralanteriorcingulate_thickness	MR Egger	93	0.0006	0.0005	0.2240	-0.0003	0.0015
Critical COVID-19 vs. general population	Mean_rostralanteriorcingulate_thickness	Weighted median	93	-0.0001	0.0002	0.5912	-0.0005	0.0003
Critical COVID-19 vs. general population	Mean_rostralanteriorcingulate_thickness	Inverse variance weighted	93	0.0000	0.0001	0.9911	-0.0003	0.0003
Critical COVID-19 vs. general population	Mean_rostralmiddlefrontal_surface_area	MR Egger	93	-0.0832	1.0878	0.9392	-2.2154	2.0489
Critical COVID-19 vs. general population	Mean_rostralmiddlefrontal_surface_area	Weighted median	93	0.1069	0.4744	0.8217	-0.8229	1.0367
Critical COVID-19 vs. general population	Mean_rostralmiddlefrontal_surface_area	Inverse variance weighted	93	0.2414	0.3289	0.4630	-0.4033	0.8861
Critical COVID-19 vs. general population	Mean_rostralmiddlefrontal_thickness	MR Egger	93	-0.0001	0.0002	0.7930	-0.0005	0.0004
Critical COVID-19 vs. general population	Mean_rostralmiddlefrontal_thickness	Weighted median	93	0.0001	0.0001	0.1492	0.0000	0.0003
Critical COVID-19 vs. general population	Mean_rostralmiddlefrontal_thickness	Inverse variance weighted	93	0.0001	0.0001	0.2007	0.0000	0.0002
Critical COVID-19 vs. general population	Mean_superiorfrontal_surface_area	MR Egger	93	0.8805	1.1391	0.4416	-1.3522	3.1131
Critical COVID-19 vs. general population	Mean_superiorfrontal_surface_area	Weighted median	93	0.2586	0.4988	0.6042	-0.7191	1.2362
Critical COVID-19 vs. general population	Mean_superiorfrontal_surface_area	Inverse variance weighted	93	0.5117	0.3439	0.1368	-0.1624	1.1858
Critical COVID-19 vs. general population	Mean_superiorfrontal_thickness	MR Egger	93	-0.0005	0.0002	0.0466	-0.0009	0.0000
Critical COVID-19 vs. general population	Mean_superiorfrontal_thickness	Weighted median	93	-0.0001	0.0001	0.3375	-0.0003	0.0001
Critical COVID-19 vs. general population	Mean_superiorfrontal_thickness	Inverse variance weighted	93	-0.0001	0.0001	0.4526	-0.0002	0.0001

continued:

COVID-19 phenotype	Brain structure	Method	nsnp	β	se	p value	β_low95%CI	β_up95%CI
Critical COVID-19 vs. general population	Mean_superiorparietal_surface_area	MR Egger	93	0.1522	1.2666	0.9046	-2.3304	2.6348
Critical COVID-19 vs. general population	Mean_superiorparietal_surface_area	Weighted median	93	0.3618	0.4782	0.4493	-0.5755	1.2991
Critical COVID-19 vs. general population	Mean_superiorparietal_surface_area	Inverse variance weighted	93	-0.1066	0.3823	0.7803	-0.8559	0.6427
Critical COVID-19 vs. general population	Mean_superiorparietal_thickness	MR Egger	93	0.0000	0.0002	0.8924	-0.0004	0.0005
Critical COVID-19 vs. general population	Mean_superiorparietal_thickness	Weighted median	93	0.0000	0.0001	0.8951	-0.0002	0.0002
Critical COVID-19 vs. general population	Mean_superiorparietal_thickness	Inverse variance weighted	93	0.0000	0.0001	0.9690	-0.0001	0.0001
Critical COVID-19 vs. general population	Mean_superiortemporal_surface_area	MR Egger	93	-0.2993	0.6280	0.6348	-1.5302	0.9317
Critical COVID-19 vs. general population	Mean_superiortemporal_surface_area	Weighted median	93	-0.2464	0.2795	0.3781	-0.7943	0.3015
Critical COVID-19 vs. general population	Mean_superiortemporal_surface_area	Inverse variance weighted	93	-0.0953	0.1898	0.6158	-0.4674	0.2768
Critical COVID-19 vs. general population	Mean_superiortemporal_thickness	MR Egger	93	0.0002	0.0003	0.5680	-0.0004	0.0007
Critical COVID-19 vs. general population	Mean_superiortemporal_thickness	Weighted median	93	0.0001	0.0001	0.5582	-0.0002	0.0003
Critical COVID-19 vs. general population	Mean_superiortemporal_thickness	Inverse variance weighted	93	0.0000	0.0001	0.5782	-0.0001	0.0002
Critical COVID-19 vs. general population	Mean_supramarginal_surface_area	MR Egger	93	0.9895	0.8713	0.2591	-0.7182	2.6973
Critical COVID-19 vs. general population	Mean_supramarginal_surface_area	Weighted median	93	0.2886	0.3763	0.4431	-0.4490	1.0262
Critical COVID-19 vs. general population	Mean_supramarginal_surface_area	Inverse variance weighted	93	0.2217	0.2643	0.4016	-0.2963	0.7397
Critical COVID-19 vs. general population	Mean_supramarginal_thickness	MR Egger	93	0.0000	0.0002	0.8169	-0.0003	0.0004
Critical COVID-19 vs. general population	Mean_supramarginal_thickness	Weighted median	93	0.0001	0.0001	0.5338	-0.0001	0.0002
Critical COVID-19 vs. general population	Mean_supramarginal_thickness	Inverse variance weighted	93	0.0000	0.0001	0.4028	-0.0001	0.0002
Critical COVID-19 vs. general population	Mean_temporalpole_surface_area	MR Egger	93	0.0434	0.1319	0.7431	-0.2152	0.3020
Critical COVID-19 vs. general population	Mean_temporalpole_surface_area	Weighted median	93	-0.0012	0.0588	0.9842	-0.1163	0.1140
Critical COVID-19 vs. general population	Mean_temporalpole_surface_area	Inverse variance weighted	93	0.0065	0.0399	0.8710	-0.0717	0.0847
Critical COVID-19 vs. general population	Mean_temporalpole_thickness	MR Egger	93	-0.0003	0.0007	0.7147	-0.0016	0.0011
Critical COVID-19 vs. general population	Mean_temporalpole_thickness	Weighted median	93	-0.0001	0.0003	0.7677	-0.0007	0.0005
Critical COVID-19 vs. general population	Mean_temporalpole_thickness	Inverse variance weighted	93	-0.0002	0.0002	0.3004	-0.0006	0.0002
Critical COVID-19 vs. general population	Mean_transversetemporal_surface_area	MR Egger	93	0.1259	0.1281	0.3281	-0.1251	0.3770
Critical COVID-19 vs. general population	Mean_transversetemporal_surface_area	Weighted median	93	0.0390	0.0585	0.5046	-0.0756	0.1537
Critical COVID-19 vs. general population	Mean_transversetemporal_surface_area	Inverse variance weighted	93	0.0098	0.0389	0.8005	-0.0664	0.0860
Critical COVID-19 vs. general population	Mean_transversetemporal_thickness	MR Egger	93	-0.0002	0.0005	0.6220	-0.0011	0.0007
Critical COVID-19 vs. general population	Mean_transversetemporal_thickness	Weighted median	93	0.0000	0.0002	0.8142	-0.0003	0.0004
Critical COVID-19 vs. general population	Mean_transversetemporal_thickness	Inverse variance weighted	93	-0.0001	0.0001	0.6368	-0.0003	0.0002

continued:

COVID-19 phenotype	Brain structure	Method	nsnp	β	se	p value	β_low95%CI	β_up95%CI
Critical COVID-19 vs. general population	Mean_Amygdala_volume	MR Egger	95	-1.0675	0.7749	0.1717	-2.5864	0.4514
Critical COVID-19 vs. general population	Mean_Amygdala_volume	Weighted median	95	-0.1487	0.3552	0.6755	-0.8448	0.5474
Critical COVID-19 vs. general population	Mean_Amygdala_volume	Inverse variance weighted	95	-0.0965	0.2345	0.6805	-0.5561	0.3630
Critical COVID-19 vs. general population	Mean_Caudate_volume	MR Egger	95	2.8493	1.8069	0.1182	-0.6922	6.3909
Critical COVID-19 vs. general population	Mean_Caudate_volume	Weighted median	95	-0.6711	0.7393	0.3640	-2.1202	0.7779
Critical COVID-19 vs. general population	Mean_Caudate_volume	Inverse variance weighted	95	-0.6548	0.5567	0.2395	-1.7458	0.4363
Critical COVID-19 vs. general population	Mean_Hippocampus_volume	MR Egger	95	-0.8550	1.5434	0.5809	-3.8800	2.1699
Critical COVID-19 vs. general population	Mean_Hippocampus_volume	Weighted median	95	0.0681	0.7329	0.9259	-1.3683	1.5046
Critical COVID-19 vs. general population	Mean_Hippocampus_volume	Inverse variance weighted	95	0.0326	0.4672	0.9444	-0.8831	0.9483
Critical COVID-19 vs. general population	Mean_Intracranial_volume	MR Egger	95	-265.4060	679.5921	0.6970	-1597.4065	1066.5945
Critical COVID-19 vs. general population	Mean_Intracranial_volume	Weighted median	95	-197.0670	286.6750	0.4918	-758.9499	364.8160
Critical COVID-19 vs. general population	Mean_Intracranial_volume	Inverse variance weighted	95	-180.5426	204.7139	0.3778	-581.7818	220.6966
Critical COVID-19 vs. general population	Mean_Nucleus_accumbens_volume	MR Egger	95	0.4968	0.3632	0.1747	-0.2152	1.2087
Critical COVID-19 vs. general population	Mean_Nucleus_accumbens_volume	Weighted median	95	0.0835	0.1638	0.6101	-0.2375	0.4046
Critical COVID-19 vs. general population	Mean_Nucleus_accumbens_volume	Inverse variance weighted	95	-0.0909	0.1110	0.4129	-0.3084	0.1266
Critical COVID-19 vs. general population	Mean_Pallidum_volume	MR Egger	95	-0.3249	0.6942	0.6409	-1.6854	1.0357
Critical COVID-19 vs. general population	Mean_Pallidum_volume	Weighted median	95	-0.1740	0.2927	0.5523	-0.7477	0.3997
Critical COVID-19 vs. general population	Mean_Pallidum_volume	Inverse variance weighted	95	-0.0166	0.2095	0.9368	-0.4273	0.3941
Critical COVID-19 vs. general population	Mean_Putamen_volume	MR Egger	95	1.7077	1.9745	0.3893	-2.1622	5.5777
Critical COVID-19 vs. general population	Mean_Putamen_volume	Weighted median	95	0.3391	0.8979	0.7057	-1.4209	2.0990
Critical COVID-19 vs. general population	Mean_Putamen_volume	Inverse variance weighted	95	-0.2248	0.5987	0.7073	-1.3983	0.9486
Critical COVID-19 vs. general population	Mean_Thalamus_volume	MR Egger	95	-2.3663	2.0532	0.2521	-6.3906	1.6579
Critical COVID-19 vs. general population	Mean_Thalamus_volume	Weighted median	95	0.1231	0.9584	0.8978	-1.7553	2.0015
Critical COVID-19 vs. general population	Mean_Thalamus_volume	Inverse variance weighted	95	-0.0729	0.6228	0.9068	-1.2935	1.1477

Abbreviations: COVID-19, coronavirus disease 2019; CI, confidence interval; nSNPs, number of single-nucleotide polymorphisms; se, standard error

Table S8. Characteristics of selected SNPs for COVID-19 phenotypes

SNP	COVID-19 phenotypes	Chr	Pos.	Effect allele	Other allele	EAF	β	se	p value	R^2	F statistic
rs10911262	COVID-19 vs. general population	1	183114956	G	A	0.5394	-0.0688	0.0154	8.47E-06	0.0024	3060.9842
rs10915271	COVID-19 vs. general population	1	29754510	G	A	0.3998	0.0699	0.0155	6.61E-06	0.0023	3054.4209
rs1185700	COVID-19 vs. general population	1	156450719	A	G	0.2734	-0.0886	0.0185	1.77E-06	0.0031	4062.9526
rs142845637	COVID-19 vs. general population	1	81539862	T	C	0.0335	0.2096	0.0467	7.22E-06	0.0028	3709.5925
rs6756041	COVID-19 vs. general population	2	166368788	T	C	0.4667	-0.0687	0.0145	2.09E-06	0.0024	3060.2972
rs72840835	COVID-19 vs. general population	2	119176557	T	C	0.1184	-0.1180	0.0258	4.75E-06	0.0029	3788.2707
rs34326463	COVID-19 vs. general population	3	45899651	G	A	0.0833	0.2772	0.0258	7.37E-27	0.0117	15414.8796
rs73062389	COVID-19 vs. general population	3	45835417	A	G	0.0566	0.2071	0.0340	1.09E-09	0.0046	5974.6736
rs7671686	COVID-19 vs. general population	4	66216523	T	C	0.5801	-0.0702	0.0154	5.39E-06	0.0024	3122.8395
rs75309214	COVID-19 vs. general population	5	163139778	G	A	0.0516	0.1802	0.0396	5.23E-06	0.0032	4139.2415
rs111837807	COVID-19 vs. general population	6	31121232	C	T	0.0921	0.1319	0.0256	2.55E-07	0.0029	3789.2039
rs114371775	COVID-19 vs. general population	6	43199730	C	T	0.1171	0.1196	0.0258	3.62E-06	0.0030	3852.2400
rs806841	COVID-19 vs. general population	6	81028433	G	A	0.8914	-0.1271	0.0286	8.65E-06	0.0031	4078.2140
rs10087754	COVID-19 vs. general population	8	122832148	A	T	0.5804	-0.0671	0.0147	4.70E-06	0.0022	2856.9427
rs62517724	COVID-19 vs. general population	8	79553287	T	A	0.1170	0.1158	0.0256	6.36E-06	0.0028	3607.3212
rs116865546	COVID-19 vs. general population	9	16874940	A	T	0.0180	0.2573	0.0558	4.08E-06	0.0023	3054.0178
rs2210421	COVID-19 vs. general population	9	1460365	C	A	0.4345	-0.0760	0.0169	7.05E-06	0.0028	3698.0409
rs505922	COVID-19 vs. general population	9	136149229	C	T	0.3435	0.0920	0.0151	1.06E-09	0.0038	4977.2861
rs4766664	COVID-19 vs. general population	12	113362997	G	T	0.6727	0.0725	0.0152	1.76E-06	0.0023	3014.0585
rs2761929	COVID-19 vs. general population	13	30401179	A	C	0.0811	-0.1806	0.0394	4.45E-06	0.0049	6344.2920
rs75933622	COVID-19 vs. general population	13	61941816	A	G	0.0399	-0.2211	0.0489	6.06E-06	0.0037	4880.6421
rs117169628	COVID-19 vs. general population	16	89262657	A	G	0.1513	0.0940	0.0201	3.04E-06	0.0023	2955.0845
rs116698469	COVID-19 vs. general population	18	60001743	G	A	0.0262	0.2199	0.0490	7.06E-06	0.0025	3218.5611
rs8096771	COVID-19 vs. general population	18	20925377	C	T	0.6743	0.0865	0.0172	5.05E-07	0.0033	4279.8841
rs2277732	COVID-19 vs. general population	19	4723670	A	C	0.3165	0.0965	0.0168	9.73E-09	0.0040	5255.5262
rs7257133	COVID-19 vs. general population	19	48239435	G	A	0.5064	-0.0684	0.0153	7.88E-06	0.0023	3047.7787
rs117710549	COVID-19 vs. general population	20	59177969	T	C	0.0242	0.2385	0.0524	5.26E-06	0.0027	3503.1009
rs157807	COVID-19 vs. general population	20	456473	G	A	0.3591	0.0858	0.0161	9.41E-08	0.0034	4411.7254
rs12482060	COVID-19 vs. general population	21	34611571	G	C	0.3380	0.0925	0.0161	9.03E-09	0.0038	4996.3038
rs7287341	COVID-19 vs. general population	22	19266865	G	A	0.1876	0.0882	0.0196	6.69E-06	0.0024	3087.9623
rs73174327	COVID-19 vs. general population	22	44089340	A	G	0.0301	0.2558	0.0535	1.72E-06	0.0038	4975.1128

continued:

SNP	COVID-19 phenotypes	Chr	Pos.	Effect allele	Other allele	EAF	β	se	p value	R^2	F statistic
rs4656878	Hospitalized COVID-19 vs. general population	1	160078149	A	G	0.4704	0.0948	0.0205	3.91E-06	0.0045	4084.9320
rs62170270	Hospitalized COVID-19 vs. general population	2	170705789	G	A	0.1993	0.1104	0.0247	7.66E-06	0.0039	3547.7994
rs78679624	Hospitalized COVID-19 vs. general population	2	238931484	T	G	0.1026	0.1827	0.0401	5.25E-06	0.0061	5616.8886
rs13097837	Hospitalized COVID-19 vs. general population	3	34737266	T	C	0.2006	0.0976	0.0215	5.65E-06	0.0031	2784.9504
rs10000811	Hospitalized COVID-19 vs. general population	4	136526689	C	T	0.4000	-0.0790	0.0160	7.64E-07	0.0030	2730.6299
rs116335024	Hospitalized COVID-19 vs. general population	4	185078909	T	C	0.0791	0.3459	0.0732	2.29E-06	0.0174	16111.0861
rs4130109	Hospitalized COVID-19 vs. general population	4	101217080	A	T	0.8003	-0.1090	0.0221	8.48E-07	0.0038	3461.3651
rs72691535	Hospitalized COVID-19 vs. general population	4	184489167	A	C	0.1115	0.1735	0.0339	3.05E-07	0.0060	5447.8926
rs72793594	Hospitalized COVID-19 vs. general population	5	117808035	T	C	0.1645	-0.1333	0.0297	7.01E-06	0.0049	4457.7860
rs75309214	Hospitalized COVID-19 vs. general population	5	163139778	G	A	0.0990	0.2024	0.0428	2.22E-06	0.0073	6682.6390
rs3997868	Hospitalized COVID-19 vs. general population	6	32578590	G	A	0.2434	-0.0870	0.0196	8.59E-06	0.0028	2541.9048
rs9462875	Hospitalized COVID-19 vs. general population	6	43168117	G	A	0.2352	0.0963	0.0194	6.58E-07	0.0033	3039.8362
rs10242392	Hospitalized COVID-19 vs. general population	7	62156283	G	T	0.8349	-0.1589	0.0359	9.52E-06	0.0070	6366.5469
rs529565	Hospitalized COVID-19 vs. general population	9	136149500	C	T	0.3500	0.0716	0.0161	8.97E-06	0.0023	2122.6027
rs4746645	Hospitalized COVID-19 vs. general population	10	68626963	C	T	0.4590	0.0799	0.0180	8.82E-06	0.0032	2888.9931
rs10161245	Hospitalized COVID-19 vs. general population	12	19805407	T	C	0.6405	-0.0831	0.0183	5.37E-06	0.0032	2901.7345
rs149487444	Hospitalized COVID-19 vs. general population	12	63734394	C	T	0.1296	0.1403	0.0306	4.64E-06	0.0044	4051.3419
rs1334373	Hospitalized COVID-19 vs. general population	13	82079715	T	C	0.1835	0.0998	0.0223	7.99E-06	0.0030	2717.3985
rs147942333	Hospitalized COVID-19 vs. general population	13	90335005	A	T	0.0754	0.4154	0.0801	2.17E-07	0.0241	22388.9336
rs17798800	Hospitalized COVID-19 vs. general population	13	34950527	T	C	0.2375	0.0991	0.0222	8.40E-06	0.0036	3243.9380
rs75933622	Hospitalized COVID-19 vs. general population	13	61941816	A	G	0.0894	-0.2689	0.0572	2.57E-06	0.0118	10815.1590
rs78101397	Hospitalized COVID-19 vs. general population	16	9734777	C	A	0.0249	0.2463	0.0513	1.55E-06	0.0029	2684.8237
rs436772	Hospitalized COVID-19 vs. general population	17	32206180	T	C	0.8542	0.1513	0.0320	2.25E-06	0.0057	5210.5937
rs9304296	Hospitalized COVID-19 vs. general population	18	41430061	G	A	0.6498	0.0753	0.0167	6.27E-06	0.0026	2350.3059
rs144495330	Hospitalized COVID-19 vs. general population	19	36230870	G	A	0.0805	0.2974	0.0634	2.68E-06	0.0131	12059.3194
rs77619227	Hospitalized COVID-19 vs. general population	21	36364903	T	G	0.0863	0.2826	0.0529	9.40E-08	0.0126	11583.2316
rs112317747	Hospitalized COVID-19 vs. non-hospitalized COVID-19	1	239197542	C	T	0.0335	0.5855	0.1312	8.10E-06	0.0222	186.4066
rs2224986	Hospitalized COVID-19 vs. non-hospitalized COVID-19	1	152684866	T	C	0.1809	-0.3107	0.0696	8.07E-06	0.0286	241.9150
rs56229346	Hospitalized COVID-19 vs. non-hospitalized COVID-19	1	186849313	A	C	0.1150	0.9491	0.2058	4.01E-06	0.1833	1844.6735
rs2034831	Hospitalized COVID-19 vs. non-hospitalized COVID-19	2	182353446	C	A	0.1543	0.3780	0.0814	3.39E-06	0.0373	318.3357
rs77695931	Hospitalized COVID-19 vs. non-hospitalized COVID-19	2	207133409	A	G	0.1248	0.6195	0.1394	8.84E-06	0.0838	751.8197
rs13062942	Hospitalized COVID-19 vs. non-hospitalized COVID-19	3	62936766	A	G	0.5946	0.2585	0.0562	4.14E-06	0.0322	273.6107

continued:

SNP	COVID-19 phenotypes	Chr	Pos.	Effect allele	Other allele	EAF	β	se	p value	R^2	F statistic
rs13079478	Hospitalized COVID-19 vs. non-hospitalized COVID-19	3	46007823	T	G	0.1857	0.3490	0.0629	2.84E-08	0.0368	314.2796
rs76488148	Hospitalized COVID-19 vs. non-hospitalized COVID-19	3	148718087	T	G	0.1631	0.3745	0.0832	6.82E-06	0.0383	327.0459
rs114776680	Hospitalized COVID-19 vs. non-hospitalized COVID-19	5	124682392	A	G	0.1216	0.6880	0.1546	8.63E-06	0.1011	924.3658
rs4478338	Hospitalized COVID-19 vs. non-hospitalized COVID-19	5	169590905	G	T	0.1921	-0.3253	0.0696	3.00E-06	0.0328	278.9734
rs6967210	Hospitalized COVID-19 vs. non-hospitalized COVID-19	7	152960930	C	T	0.0628	0.4224	0.0938	6.63E-06	0.0210	176.3061
rs79385393	Hospitalized COVID-19 vs. non-hospitalized COVID-19	7	24226722	A	C	0.1207	1.1016	0.2200	5.50E-07	0.2576	2850.9481
rs139368606	Hospitalized COVID-19 vs. non-hospitalized COVID-19	9	100859508	G	T	0.1149	0.9251	0.2068	7.70E-06	0.1741	1731.8100
rs71480372	Hospitalized COVID-19 vs. non-hospitalized COVID-19	9	27121456	A	T	0.6479	0.2650	0.0561	2.35E-06	0.0320	271.9256
rs75256341	Hospitalized COVID-19 vs. non-hospitalized COVID-19	11	120540021	A	C	0.1224	0.8328	0.1559	9.20E-08	0.1490	1438.5898
rs2649134	Hospitalized COVID-19 vs. non-hospitalized COVID-19	13	63178476	C	T	0.8136	-0.7950	0.1760	6.27E-06	0.1917	1948.6531
rs77055952	Hospitalized COVID-19 vs. non-hospitalized COVID-19	15	45858905	G	A	0.1364	0.4404	0.0970	5.60E-06	0.0457	393.4385
rs9890316	Hospitalized COVID-19 vs. non-hospitalized COVID-19	17	80443309	A	G	0.4048	-0.2702	0.0605	7.96E-06	0.0352	299.5744
rs142257532	Hospitalized COVID-19 vs. non-hospitalized COVID-19	18	30006171	C	T	0.1301	0.5595	0.1232	5.54E-06	0.0709	626.7205
rs76253189	Hospitalized COVID-19 vs. non-hospitalized COVID-19	20	60473717	G	C	0.1367	0.4955	0.1110	8.04E-06	0.0579	505.4380
rs10864082	Severe COVID-19 vs. general population	1	214111247	G	A	0.9481	-0.2478	0.0489	4.14E-07	0.0060	6440.1812
rs1933129	Severe COVID-19 vs. general population	1	237277611	A	T	0.0458	-0.2866	0.0585	9.51E-07	0.0072	7662.8232
rs4076440	Severe COVID-19 vs. general population	1	9690476	G	A	0.1006	0.2004	0.0416	1.47E-06	0.0073	7757.3504
rs67579710	Severe COVID-19 vs. general population	1	155173527	A	G	0.0728	-0.2110	0.0420	5.10E-07	0.0060	6404.7654
rs76659818	Severe COVID-19 vs. general population	1	188500701	T	C	0.0169	0.5672	0.1121	4.17E-07	0.0107	11439.8245
rs113488799	Severe COVID-19 vs. general population	2	229773795	C	T	0.3245	0.1756	0.0372	2.31E-06	0.0135	14518.1776
rs55917944	Severe COVID-19 vs. general population	2	66815140	G	T	0.1707	-0.2551	0.0568	7.06E-06	0.0184	19887.9120
rs73980984	Severe COVID-19 vs. general population	2	188814713	G	T	0.1673	0.2058	0.0459	7.31E-06	0.0118	12652.7662
rs7572273	Severe COVID-19 vs. general population	2	144174295	A	G	0.9161	-0.2408	0.0464	2.07E-07	0.0089	9529.9612
rs35081325	Severe COVID-19 vs. general population	3	45889921	T	A	0.0800	0.6327	0.0444	5.29E-46	0.0589	66315.1068
rs73172611	Severe COVID-19 vs. general population	3	117476177	A	G	0.4106	0.1500	0.0333	6.59E-06	0.0109	11658.5637
rs73212329	Severe COVID-19 vs. general population	3	197122814	T	C	0.0950	-0.2560	0.0489	1.60E-07	0.0113	12069.2158
rs6867867	Severe COVID-19 vs. general population	5	54092505	C	T	0.9425	-0.2525	0.0553	5.00E-06	0.0069	7372.7629
rs111837807	Severe COVID-19 vs. general population	6	31121232	C	T	0.1300	0.2936	0.0410	7.93E-13	0.0195	21074.5979
rs58944290	Severe COVID-19 vs. general population	7	139657886	G	A	0.0916	-0.2160	0.0468	4.00E-06	0.0078	8282.4699
rs622568	Severe COVID-19 vs. general population	7	54647894	C	A	0.1728	0.2113	0.0352	1.95E-09	0.0128	13698.9086
rs10109219	Severe COVID-19 vs. general population	8	59917788	A	G	0.0542	-0.2162	0.0473	4.80E-06	0.0048	5100.6742
rs117184073	Severe COVID-19 vs. general population	8	22044324	C	G	0.0095	-0.4650	0.0993	2.85E-06	0.0041	4344.8093

continued:

SNP	COVID-19 phenotypes	Chr	Pos.	Effect allele	Other allele	EAF	β	se	p value	R^2	F statistic
rs11777988	Severe COVID-19 vs. general population	8	101370866	A	G	0.1254	0.2063	0.0447	3.84E-06	0.0093	9986.6180
rs4366152	Severe COVID-19 vs. general population	9	117564875	C	T	0.6998	0.1364	0.0275	6.99E-07	0.0078	8342.1299
rs11019591	Severe COVID-19 vs. general population	11	88104725	T	C	0.3890	-0.1271	0.0267	2.00E-06	0.0077	8193.4418
rs11223261	Severe COVID-19 vs. general population	11	132755195	G	C	0.0288	0.2969	0.0652	5.30E-06	0.0049	5241.7226
rs78123456	Severe COVID-19 vs. general population	11	28447962	C	T	0.0262	0.3162	0.0702	6.69E-06	0.0051	5435.2304
rs78594643	Severe COVID-19 vs. general population	11	22828273	T	C	0.0210	-0.4626	0.1019	5.58E-06	0.0088	9412.7171
rs10735079	Severe COVID-19 vs. general population	12	113380008	A	G	0.6961	0.2005	0.0272	1.55E-13	0.0170	18325.7230
rs10746119	Severe COVID-19 vs. general population	12	108588927	G	A	0.8065	-0.1487	0.0335	8.81E-06	0.0069	7362.5289
rs10860891	Severe COVID-19 vs. general population	12	103014757	A	C	0.8666	-0.2211	0.0383	7.85E-09	0.0113	12109.3624
rs2467456	Severe COVID-19 vs. general population	12	23015226	C	T	0.6715	0.1612	0.0364	9.50E-06	0.0115	12289.6679
rs12876544	Severe COVID-19 vs. general population	13	22392842	C	T	0.4948	0.1602	0.0344	3.14E-06	0.0128	13763.1792
rs9577175	Severe COVID-19 vs. general population	13	113543355	T	C	0.2460	0.2034	0.0401	4.05E-07	0.0153	16513.4370
rs11634857	Severe COVID-19 vs. general population	15	79766794	A	G	0.2181	-0.1486	0.0313	1.98E-06	0.0075	8041.8801
rs2580926	Severe COVID-19 vs. general population	15	49533127	T	C	0.3397	-0.1261	0.0267	2.43E-06	0.0071	7611.8149
rs1007721	Severe COVID-19 vs. general population	16	54550376	A	G	0.6872	-0.1300	0.0288	6.19E-06	0.0073	7749.0121
rs4528629	Severe COVID-19 vs. general population	17	71768878	C	T	0.6499	-0.1223	0.0260	2.67E-06	0.0068	7255.7732
rs77534576	Severe COVID-19 vs. general population	17	47940666	T	C	0.0410	0.4006	0.0724	3.09E-08	0.0126	13530.7512
rs2042908	Severe COVID-19 vs. general population	19	53626513	A	C	0.6051	-0.1193	0.0256	3.19E-06	0.0068	7258.0166
rs2109069	Severe COVID-19 vs. general population	19	4719443	A	G	0.3310	0.2615	0.0274	1.46E-21	0.0303	33079.7880
rs2112663	Severe COVID-19 vs. general population	19	50852276	G	A	0.4181	-0.1193	0.0266	7.53E-06	0.0069	7391.9585
rs13050728	Severe COVID-19 vs. general population	21	34615210	C	T	0.6374	-0.1923	0.0274	2.31E-12	0.0171	18414.8624
rs114173811	Severe COVID-19 with respiratory failure vs. general population	1	161223416	T	C	0.0309	1.1809	0.2669	9.70E-06	0.0835	345.1991
rs75558547	Severe COVID-19 with respiratory failure vs. general population	1	88993151	T	C	0.0497	-0.7576	0.1596	2.06E-06	0.0542	217.1426
rs35344266	Severe COVID-19 with respiratory failure vs. general population	2	130468648	T	A	0.6027	0.3945	0.0744	1.13E-07	0.0745	305.0650
rs35731912	Severe COVID-19 with respiratory failure vs. general population	3	45889949	T	C	0.0769	0.7287	0.1103	3.98E-11	0.0754	308.8539
rs9882319	Severe COVID-19 with respiratory failure vs. general population	3	8253480	G	A	0.4597	-0.3039	0.0676	6.94E-06	0.0459	182.1406
rs73434199	Severe COVID-19 with respiratory failure vs. general population	6	44518128	A	G	0.1205	0.4490	0.0948	2.17E-06	0.0427	169.0915
rs2440652	Severe COVID-19 with respiratory failure vs. general population	8	80644873	T	G	0.3944	-0.2984	0.0659	5.88E-06	0.0425	168.2820
rs76670680	Severe COVID-19 with respiratory failure vs. general population	8	22050435	T	C	0.0162	-0.8321	0.1844	6.43E-06	0.0221	85.4880
rs657152	Severe COVID-19 with respiratory failure vs. general population	9	136139265	A	C	0.3604	0.3314	0.0661	5.35E-07	0.0506	202.0244
rs3934992	Severe COVID-19 with respiratory failure vs. general population	11	771716	C	A	0.5319	0.3259	0.0663	8.73E-07	0.0529	211.5322
rs78243302	Severe COVID-19 with respiratory failure vs. general population	11	87561583	A	G	0.0230	0.8433	0.1889	8.04E-06	0.0320	125.0644

continued:

SNP	COVID-19 phenotypes	Chr	Pos.	Effect allele	Other allele	EAF	β	se	p value	R^2	F statistic
rs9598460	Severe COVID-19 with respiratory failure vs. general population	13	63042159	T	G	0.1028	0.5010	0.1107	5.98E-06	0.0463	183.9020
rs2279379	Severe COVID-19 with respiratory failure vs. general population	15	74421010	G	C	0.7558	0.3371	0.0746	6.25E-06	0.0419	165.8519
rs79300914	Severe COVID-19 with respiratory failure vs. general population	16	82391520	C	T	0.9639	-0.8966	0.1867	1.57E-06	0.0559	224.4810
rs12610495	Severe COVID-19 with respiratory failure vs. general population	19	4717672	G	A	0.7018	0.3159	0.0704	7.16E-06	0.0418	165.1163
rs134130	Severe COVID-19 with respiratory failure vs. general population	22	28128191	C	T	0.5333	-0.2876	0.0648	9.14E-06	0.0412	162.6624
rs10915756	Critical COVID-19 vs. general population	1	225177169	T	A	0.8794	1.1706	0.1638	8.96E-13	0.2907	446.6547
rs112115136	Critical COVID-19 vs. general population	1	11037434	A	G	0.2287	-0.7924	0.1560	3.76E-07	0.2215	310.1915
rs116776358	Critical COVID-19 vs. general population	1	179337119	T	C	0.0891	0.8825	0.1714	2.62E-07	0.1264	157.7469
rs2072941	Critical COVID-19 vs. general population	1	209811595	C	T	0.5176	1.1306	0.2280	7.07E-07	0.6384	1924.1317
rs2772337	Critical COVID-19 vs. general population	1	90559504	T	C	0.7406	-1.1468	0.2118	6.18E-08	0.5053	1113.5631
rs4646999	Critical COVID-19 vs. general population	1	59250452	G	A	0.3564	0.6933	0.1542	6.91E-06	0.2205	308.3631
rs6673075	Critical COVID-19 vs. general population	1	116777027	A	T	0.1264	-1.5833	0.3397	3.15E-06	0.5536	1351.8960
rs6704072	Critical COVID-19 vs. general population	1	189322373	G	A	0.9341	-0.7094	0.1560	5.43E-06	0.0620	71.9884
rs821549	Critical COVID-19 vs. general population	1	155709395	T	C	0.0511	-1.1255	0.2105	9.01E-08	0.1228	152.6527
rs12617081	Critical COVID-19 vs. general population	2	209819732	A	T	0.2665	-0.7871	0.1556	4.25E-07	0.2422	348.3740
rs1473370	Critical COVID-19 vs. general population	2	237129238	C	A	0.6653	-1.4197	0.2779	3.24E-07	0.8976	9554.8140
rs3791238	Critical COVID-19 vs. general population	2	138760816	A	T	0.2045	0.7025	0.1580	8.78E-06	0.1606	208.5011
rs4547559	Critical COVID-19 vs. general population	2	54632770	T	C	0.6465	-0.9934	0.2139	3.43E-06	0.4511	895.7476
rs4954167	Critical COVID-19 vs. general population	2	135460254	A	T	0.1434	1.5213	0.2000	2.79E-14	0.5685	1436.2859
rs78904011	Critical COVID-19 vs. general population	2	125183636	C	A	0.9440	1.0155	0.2255	6.69E-06	0.1090	133.3900
rs13314348	Critical COVID-19 vs. general population	3	102014659	T	C	0.0654	0.9587	0.1666	8.67E-09	0.1123	137.9555
rs1471696	Critical COVID-19 vs. general population	3	176027253	T	C	0.7217	-1.1432	0.1762	8.75E-11	0.5249	1204.4276
rs34834532	Critical COVID-19 vs. general population	3	2394048	A	G	0.2867	-1.3275	0.2795	2.04E-06	0.7207	2813.0375
rs34885404	Critical COVID-19 vs. general population	3	32648031	G	T	0.7613	-0.9592	0.1768	5.79E-08	0.3344	547.6036
rs4524322	Critical COVID-19 vs. general population	3	144029682	T	G	0.7647	-0.8427	0.1563	7.02E-08	0.2555	374.1678
rs7646439	Critical COVID-19 vs. general population	3	112580237	C	T	0.3972	-0.8616	0.1600	7.27E-08	0.3555	601.1759
rs10939740	Critical COVID-19 vs. general population	4	17617490	C	T	0.3879	-0.7634	0.1564	1.05E-06	0.2767	417.0546
rs2667365	Critical COVID-19 vs. general population	4	140781918	C	T	0.5299	-1.3092	0.2876	5.32E-06	0.8539	6371.2335
rs34779602	Critical COVID-19 vs. general population	4	137611100	C	T	0.8744	1.7544	0.2135	2.09E-16	0.6761	2275.0096
rs356224	Critical COVID-19 vs. general population	4	90643623	G	A	0.2925	-1.0975	0.1813	1.41E-09	0.4985	1083.4524
rs3796964	Critical COVID-19 vs. general population	4	104568816	T	C	0.3312	-0.8204	0.1834	7.67E-06	0.2982	463.0549
rs7660379	Critical COVID-19 vs. general population	4	63703335	A	T	0.2755	-0.9073	0.1699	9.30E-08	0.3286	533.5755

continued:

SNP	COVID-19 phenotypes	Chr	Pos.	Effect allele	Other allele	EAF	β	se	p value	R^2	F statistic
rs9994283	Critical COVID-19 vs. general population	4	114690756	C	T	0.6773	-0.7483	0.1564	1.72E-06	0.2448	353.3238
rs10050803	Critical COVID-19 vs. general population	5	60038225	A	G	0.4335	-0.9496	0.1735	4.40E-08	0.4429	866.4961
rs10074958	Critical COVID-19 vs. general population	5	230828	T	C	0.1353	0.7503	0.1627	3.97E-06	0.1317	165.3807
rs11241420	Critical COVID-19 vs. general population	5	116661975	G	A	0.9439	1.4156	0.1951	3.99E-13	0.2122	293.6467
rs2049494	Critical COVID-19 vs. general population	5	75135156	A	G	0.4122	0.7687	0.1708	6.73E-06	0.2864	437.3880
rs411330	Critical COVID-19 vs. general population	5	84760115	C	T	0.7383	1.3094	0.2761	2.12E-06	0.6625	2139.5159
rs6890693	Critical COVID-19 vs. general population	5	64706374	C	T	0.4516	-1.1100	0.2018	3.78E-08	0.6103	1707.1773
rs6895966	Critical COVID-19 vs. general population	5	66969398	T	C	0.4541	-1.3569	0.2352	7.94E-09	0.9128	11410.5552
rs962478	Critical COVID-19 vs. general population	5	89240065	G	A	0.6690	-1.3443	0.2764	1.15E-06	0.8003	4368.4040
rs12205816	Critical COVID-19 vs. general population	6	113076261	T	G	0.1242	1.7141	0.2054	7.10E-17	0.6392	1930.8418
rs1407532	Critical COVID-19 vs. general population	6	131609166	A	G	0.4952	-1.3089	0.2864	4.88E-06	0.8565	6506.7491
rs192741965	Critical COVID-19 vs. general population	6	153573327	T	A	0.9591	1.0290	0.2164	1.98E-06	0.0831	98.7503
rs7775024	Critical COVID-19 vs. general population	6	163769152	A	G	0.2298	1.0217	0.1820	1.98E-08	0.3695	638.8832
rs9375687	Critical COVID-19 vs. general population	6	130288255	A	G	0.1019	0.7680	0.1703	6.46E-06	0.1080	131.9178
rs2189718	Critical COVID-19 vs. general population	7	15023681	C	G	0.0809	0.8099	0.1648	8.97E-07	0.0975	117.8084
rs28419654	Critical COVID-19 vs. general population	7	9821037	T	C	0.0376	1.0968	0.1744	3.23E-10	0.0871	103.9474
rs34762928	Critical COVID-19 vs. general population	7	139113168	A	T	0.2369	-0.9445	0.2095	6.51E-06	0.3226	518.9974
rs622568	Critical COVID-19 vs. general population	7	54647894	C	A	0.8302	1.7245	0.1848	1.06E-20	0.8384	5655.0780
rs73719044	Critical COVID-19 vs. general population	7	124400676	C	A	0.9203	0.7727	0.1716	6.74E-06	0.0876	104.6350
rs1460236	Critical COVID-19 vs. general population	8	105899131	C	T	0.3006	-1.1214	0.1739	1.13E-10	0.5287	1222.8575
rs1550893	Critical COVID-19 vs. general population	8	3198374	T	C	0.7319	-1.1889	0.2482	1.67E-06	0.5547	1357.6604
rs28647270	Critical COVID-19 vs. general population	8	139550487	G	A	0.8325	-1.2385	0.2410	2.76E-07	0.4278	814.7947
rs72621436	Critical COVID-19 vs. general population	8	59575432	T	C	0.0515	-1.8505	0.3289	1.83E-08	0.3346	548.0003
rs10733439	Critical COVID-19 vs. general population	9	2962418	T	G	0.6585	-0.9889	0.2195	6.60E-06	0.4398	855.7263
rs11140408	Critical COVID-19 vs. general population	9	86778912	A	T	0.3603	-0.6955	0.1561	8.43E-06	0.2230	312.7469
rs224020	Critical COVID-19 vs. general population	9	20315337	T	G	0.3701	-1.4427	0.2889	5.92E-07	0.9704	35728.4382
rs2456260	Critical COVID-19 vs. general population	9	76300081	A	C	0.3379	-1.1436	0.2410	2.08E-06	0.5852	1537.8453
rs7862050	Critical COVID-19 vs. general population	9	14072641	A	C	0.0954	-1.1818	0.1745	1.27E-11	0.2411	346.2113
rs10881686	Critical COVID-19 vs. general population	10	91604911	A	G	0.0991	1.0408	0.1744	2.42E-09	0.1934	261.4073
rs12255165	Critical COVID-19 vs. general population	10	58334722	A	G	0.2907	-0.7416	0.1505	8.40E-07	0.2268	319.6992
rs1832311	Critical COVID-19 vs. general population	10	24269640	T	G	0.4927	-1.3821	0.2966	3.18E-06	0.9549	23076.7010
rs1999775	Critical COVID-19 vs. general population	10	45081343	T	C	0.4765	1.1476	0.2410	1.92E-06	0.6570	2087.7728

continued:

SNP	COVID-19 phenotypes	Chr	Pos.	Effect allele	Other allele	EAF	β	se	p value	R^2	F statistic
rs60083472	Critical COVID-19 vs. general population	10	13715607	C	G	0.0310	1.3151	0.2182	1.67E-09	0.1039	126.3964
rs7087055	Critical COVID-19 vs. general population	10	129738826	T	A	0.7996	1.1483	0.1786	1.28E-10	0.4226	797.7869
rs12786242	Critical COVID-19 vs. general population	11	41225074	A	G	0.5595	1.3473	0.2962	5.39E-06	0.8948	9272.1159
rs2555161	Critical COVID-19 vs. general population	11	6515853	T	C	0.5335	-0.6727	0.1517	9.19E-06	0.2252	316.8501
rs331537	Critical COVID-19 vs. general population	11	4471276	A	G	0.0553	2.3530	0.2790	3.37E-17	0.5785	1495.8565
rs3862764	Critical COVID-19 vs. general population	11	82027118	G	T	0.3619	-1.0917	0.2313	2.36E-06	0.5504	1334.5112
rs577975	Critical COVID-19 vs. general population	11	116722910	C	T	0.9417	-0.6940	0.1538	6.45E-06	0.0529	60.8662
rs7108161	Critical COVID-19 vs. general population	11	89363775	C	T	0.2052	-1.2415	0.2315	8.25E-08	0.5027	1101.9471
rs79580195	Critical COVID-19 vs. general population	11	26745600	A	C	0.0493	1.0098	0.1918	1.40E-07	0.0956	115.1943
rs80201639	Critical COVID-19 vs. general population	11	24792395	G	A	0.9697	0.8601	0.1772	1.21E-06	0.0435	49.5349
rs391612	Critical COVID-19 vs. general population	13	33101731	C	T	0.2498	-0.9684	0.1565	6.04E-10	0.3515	590.7131
rs57327694	Critical COVID-19 vs. general population	13	47706865	G	A	0.9748	0.8978	0.2032	9.95E-06	0.0396	44.9492
rs63732460	Critical COVID-19 vs. general population	13	74746460	A	C	0.1099	-1.0061	0.1614	4.60E-10	0.1980	269.1402
rs7981549	Critical COVID-19 vs. general population	13	89217200	A	G	0.1326	1.0687	0.2258	2.21E-06	0.2627	388.4111
rs115833473	Critical COVID-19 vs. general population	14	29017947	T	A	0.9294	1.5739	0.2186	5.96E-13	0.3251	525.0384
rs12896933	Critical COVID-19 vs. general population	14	42825018	A	C	0.0854	0.9321	0.1922	1.23E-06	0.1357	171.1659
rs2182565	Critical COVID-19 vs. general population	14	94130273	A	G	0.0359	1.2475	0.2139	5.51E-09	0.1077	131.6050
rs2295133	Critical COVID-19 vs. general population	14	97594218	T	C	0.2033	-1.5142	0.2903	1.84E-07	0.7427	3146.2449
rs55777651	Critical COVID-19 vs. general population	14	66631086	G	C	0.9015	1.2521	0.1728	4.30E-13	0.2784	420.5496
rs4775563	Critical COVID-19 vs. general population	15	63202022	G	A	0.0324	-1.0162	0.2050	7.14E-07	0.0647	75.4586
rs111067724	Critical COVID-19 vs. general population	16	48965783	A	C	0.4134	-0.8924	0.1558	1.02E-08	0.3863	686.0099
rs17707300	Critical COVID-19 vs. general population	16	28593347	C	T	0.6230	0.9634	0.1921	5.31E-07	0.4360	842.4805
rs2407618	Critical COVID-19 vs. general population	16	60951773	T	C	0.2377	-0.7810	0.1688	3.71E-06	0.2210	309.2677
rs4781374	Critical COVID-19 vs. general population	16	13092100	G	A	0.7489	1.1513	0.2144	7.94E-08	0.4985	1083.3911
rs4796791	Critical COVID-19 vs. general population	17	40530763	C	T	0.3630	1.4256	0.3028	2.50E-06	0.9399	17051.7015
rs8081219	Critical COVID-19 vs. general population	17	30261133	T	G	0.1486	1.0435	0.1751	2.56E-09	0.2755	414.5242
rs9903754	Critical COVID-19 vs. general population	17	29634468	A	G	0.3112	-1.4657	0.3157	3.45E-06	0.9210	12702.7817
rs7240102	Critical COVID-19 vs. general population	18	52252173	A	G	0.7486	-0.7995	0.1766	5.97E-06	0.2406	345.3511
rs8092105	Critical COVID-19 vs. general population	18	19515041	G	C	0.8549	-0.7333	0.1657	9.63E-06	0.1334	167.8034
rs10405309	Critical COVID-19 vs. general population	19	22647583	T	G	0.1033	0.9326	0.1951	1.76E-06	0.1611	209.3880
rs2010370	Critical COVID-19 vs. general population	19	29010095	A	G	0.1553	1.0467	0.1750	2.22E-09	0.2874	439.6503
rs7254081	Critical COVID-19 vs. general population	19	6028227	A	C	0.2787	-0.8624	0.1785	1.35E-06	0.2990	464.9154

continued:

SNP	COVID-19 phenotypes	Chr	Pos.	Effect allele	Other allele	EAF	β	se	p value	R^2	F statistic
rs74172478	Critical COVID-19 vs. general population	19	45070068	A	C	0.1630	1.6981	0.2301	1.59E-13	0.7868	4021.9088
rs139285	Critical COVID-19 vs. general population	22	39495780	C	T	0.5440	-1.2338	0.2657	3.44E-06	0.7552	3362.8424
rs2349623	Critical COVID-19 vs. general population	22	44943401	A	G	0.6330	1.2068	0.2076	6.16E-09	0.6766	2280.9455
rs4499536	Critical COVID-19 vs. general population	22	45177112	G	A	0.1155	-0.9437	0.1860	3.89E-07	0.1820	242.4663

Abbreviations: Chr, chromosome; EAF, Effect allele frequency; Pos, position; se, standard error; SNP, single-nucleotide polymorphism. The threshold was set at $p < 1\times10^{-5}$.
